# Recommendations for reproducibility of cerebrospinal fluid extracellular vesicle studies

**DOI:** 10.1002/jev2.12397

**Published:** 2023-12-29

**Authors:** Ursula S. Sandau, Setty M. Magaña, Júlia Costa, John P. Nolan, Tsuneya Ikezu, Laura J. Vella, Hannah K. Jackson, Lissette Retana Moreira, Paola Loreto Palacio, Andrew F. Hill, Joseph F. Quinn, Kendall R. Van Keuren‐Jensen, Trevor J. McFarland, Joanna Palade, Eric A. Sribnick, Huaqi Su, Kostas Vekrellis, Beth Coyle, You Yang, Juan M. Falcón‐Perez, Rienk Nieuwland, Julie A. Saugstad, International Society for Extracellular Vesicles Cerebrospinal Fluid Task Force

**Affiliations:** ^1^ Department of Anesthesiology & Perioperative Medicine Oregon Health & Science University Portland Oregon USA; ^2^ Center for Clinical and Translational Research, Abigail Wexner Research Institute Nationwide Children's Hospital Columbus Ohio USA; ^3^ Instituto de Tecnologia Química e Biológica António Xavier Universidade Nova de Lisboa, Avenida da República Oeiras Portugal; ^4^ Scintillon Institute for Biomedical and Bioenergy Research San Diego California USA; ^5^ Department of Neuroscience Mayo Clinic Florida Jacksonville Florida USA; ^6^ Department of Surgery, The Royal Melbourne Hospital The University of Melbourne Parkville Victoria Australia; ^7^ The Florey Institute of Neuroscience and Mental Health University of Melbourne Parkville, Melbourne Victoria Australia; ^8^ Department of Pathology University of Cambridge Cambridge UK; ^9^ Exosis, Inc. Palm Beach Florida USA; ^10^ Department of Parasitology, Faculty of Microbiology University of Costa Rica San José Costa Rica, Central America; ^11^ Centro de Investigación en Enfermedades Tropicales University of Costa Rica San José Costa Rica, Central America; ^12^ Institute for Health and Sport Victoria University Melbourne Victoria Australia; ^13^ Department of Biochemistry and Chemistry, La Trobe Institute for Molecular Science La Trobe University Bundoora Victoria Australia; ^14^ Department of Neurology Oregon Health & Science University Portland Oregon USA; ^15^ Portland VA Medical Center Portland Oregon USA; ^16^ Neurogenomics Division Translational Genomics Research Institute Phoenix Arizona USA; ^17^ Department of Neurosurgery Nationwide Children's Hospital, The Ohio State University Columbus Ohio USA; ^18^ Biomedical Research Foundation Academy of Athens Athens Greece; ^19^ Children's Brain Tumour Research Centre, School of Medicine University of Nottingham Biodiscovery Institute, University of Nottingham Nottingham Nottinghamshire UK; ^20^ Exosomes Laboratory, Center for Cooperative Research in Biosciences Basque Research and Technology Alliance Derio Spain; ^21^ Metabolomics Platform, Center for Cooperative Research in Biosciences Basque Research and Technology Alliance Derio Spain; ^22^ Centro de Investigación Biomédica en Red de Enfermedades Hepáticas y Digestivas Madrid Spain; ^23^ Ikerbasque, Basque Foundation for Science Bilbao Spain; ^24^ Laboratory of Experimental Clinical Chemistry, Amsterdam University Medical Centers, Location AMC University of Amsterdam Amsterdam The Netherlands; ^25^ Amsterdam Vesicle Center, Amsterdam University Medical Centers, Location AMC University of Amsterdam Amsterdam The Netherlands

**Keywords:** biomarkers, brain, central nervous system, cerebrospinal fluid, exosome, extracellular vesicle, recommendations and reporting

## Abstract

Cerebrospinal fluid (CSF) is a clear, transparent fluid derived from blood plasma that protects the brain and spinal cord against mechanical shock, provides buoyancy, clears metabolic waste and transports extracellular components to remote sites in the brain. Given its contact with the brain and the spinal cord, CSF is the most informative biofluid for studies of the central nervous system (CNS). In addition to other components, CSF contains extracellular vesicles (EVs) that carry bioactive cargoes (e.g., lipids, nucleic acids, proteins), and that can have biological functions within and beyond the CNS. Thus, CSF EVs likely serve as both mediators of and contributors to communication in the CNS. Accordingly, their potential as biomarkers for CNS diseases has stimulated much excitement for and attention to CSF EV research. However, studies on CSF EVs present unique challenges relative to EV studies in other biofluids, including the invasive nature of CSF collection, limited CSF volumes and the low numbers of EVs in CSF as compared to plasma. Here, the objectives of the International Society for Extracellular Vesicles CSF Task Force are to promote the reproducibility of CSF EV studies by providing current reporting and best practices, and recommendations and reporting guidelines, for CSF EV studies. To accomplish this, we created and distributed a world‐wide survey to ISEV members to assess methods considered ‘best practices’ for CSF EVs, then performed a detailed literature review for CSF EV publications that was used to curate methods and resources. Based on responses to the survey and curated information from publications, the CSF Task Force herein provides recommendations and reporting guidelines to promote the reproducibility of CSF EV studies in seven domains: (i) CSF Collection, Processing, and Storage; (ii) CSF EV Separation/Concentration; (iii) CSF EV Size and Number Measurements; (iv) CSF EV Protein Studies; (v) CSF EV RNA Studies; (vi) CSF EV Omics Studies and (vii) CSF EV Functional Studies.

## 1 INTRODUCTION

### 1.1 Objectives of the International society for extracellular vesicles cerebrospinal fluid task force

The International Society for Extracellular Vesicles (ISEV) guidelines for Minimal Information for Studies of Extracellular Vesicles (MISEV) 2018 (Théry et al., 2019) provided recommendations in six areas of extracellular vesicle (EV) research in order to improve the rigour, reproducibility and reporting of EV studies. Through the current document, the ISEV Cerebrospinal Fluid (CSF) Task Force seeks to promote the reproducibility of CSF EV studies by providing reporting recommendations and best practices. To accomplish this, we first administered a survey to identify the methods most used and/or preferred for CSF EV studies. We then, searched PubMed to curate publications on CSF EV studies, and queried the EV‐TRACK (Transparent Reporting and Centralizing Knowledge in Extracellular Vesicle Research) platform (Van Deun et al., 2017) (https://evtrack.org/) for ‘cerebrospinal fluid’ entries. This information was then used to prepare the current recommendations.

### 1.2 CSF task force survey, literature review and EV‐TRACK reporting

The ISEV Rigor & Standardization Subcommittee oversees Task Forces relevant to EVs, including the CSF Task Force, with members in North America, Central America, Europe and Australia with expertise in CSF and CSF EV research (https://www.isev.org/csf‐task‐force). The CSF Task Force created and administered a 25 question survey in QualtricsXM (https://www.qualtrics.com/) that covered seven broad topics: (i) CSF Collection, Processing, and Storage; (ii) CSF EV Separation/Concentration; (iii) CSF EV Size and Number Measurements; (iv) CSF EV Protein Studies; (v) CSF EV RNA Studies; (vi) CSF EV Omics Studies and (vii) CSF EV Functional Studies. The survey was distributed to all ISEV members on 24 March 2021, and the survey was closed on 30 April 2021. Data from 45 survey respondents (see Supplemental File 1 for the survey and responses) was tabulated and interpreted by all CSF Task Force members (https://isev.memberclicks.net/csf‐task‐force), and the results are reported herein.

In order to ascertain all information reported in published CSF EV studies to date, we queried the PubMed search engine (https://pubmed.ncbi.nlm.nih.gov/) using the following terms: ‘(Cerebrospinal fluid[Title/Abstract]) AND ((exosome[Title/Abstract]) OR (exosomes[Title/Abstract]) OR (extracellular vesicle[Title/Abstract]) OR (extracellular vesicles[Title/Abstract]) OR (microvesicles[Title/Abstract]) OR (microvesicle[Title/Abstract]) OR (ectosomes[Title/Abstract]) OR (ectosome[Title/Abstract])) NOT (Review[Publication Type])’. The PubMed search returned 245 results from conception of the PubMed database to the query date of 1 September 2022 (Table S1). All 245 abstracts were read in their entirety by at least one member of the CSF Task Force. The articles were then manually curated to exclude those that (i) were not published in the English language; (ii) were not an original research article (e.g., a review that was not catalogued as such in PubMed) and (iii) did not perform original research with CSF EVs and/or total (unfractionated) CSF (e.g., a plasma EV research article that referred to CSF in background information). Based on these search and exclusion criteria, we identified 158 original research articles on CSF EVs. Limitations to the search criteria include a lack of (i) the terms ‘apoptotic bodies’, ‘microparticles’ and ‘ectosomes’, which are also EVs; and (ii) a truncation symbol at the end of a root term (e.g., exosom*). An additional query of the EV‐TRACK platform identified three articles not captured by the above search criteria. In all, 161 publications (Table S2) were read by at least one CSF Task Force member and co‐author on the paper to obtain information from each article. Results are reported in the Main and Supplemental Tables. Empty fields in the Tables indicate a lack of experimental details in the referenced publication. Supplemental Tables were then read and reviewed for accuracy by the CSF TF manuscript Section Leads (see Author Contributions).

### 1.3 CSF production and functions

**FIGURE 1:**
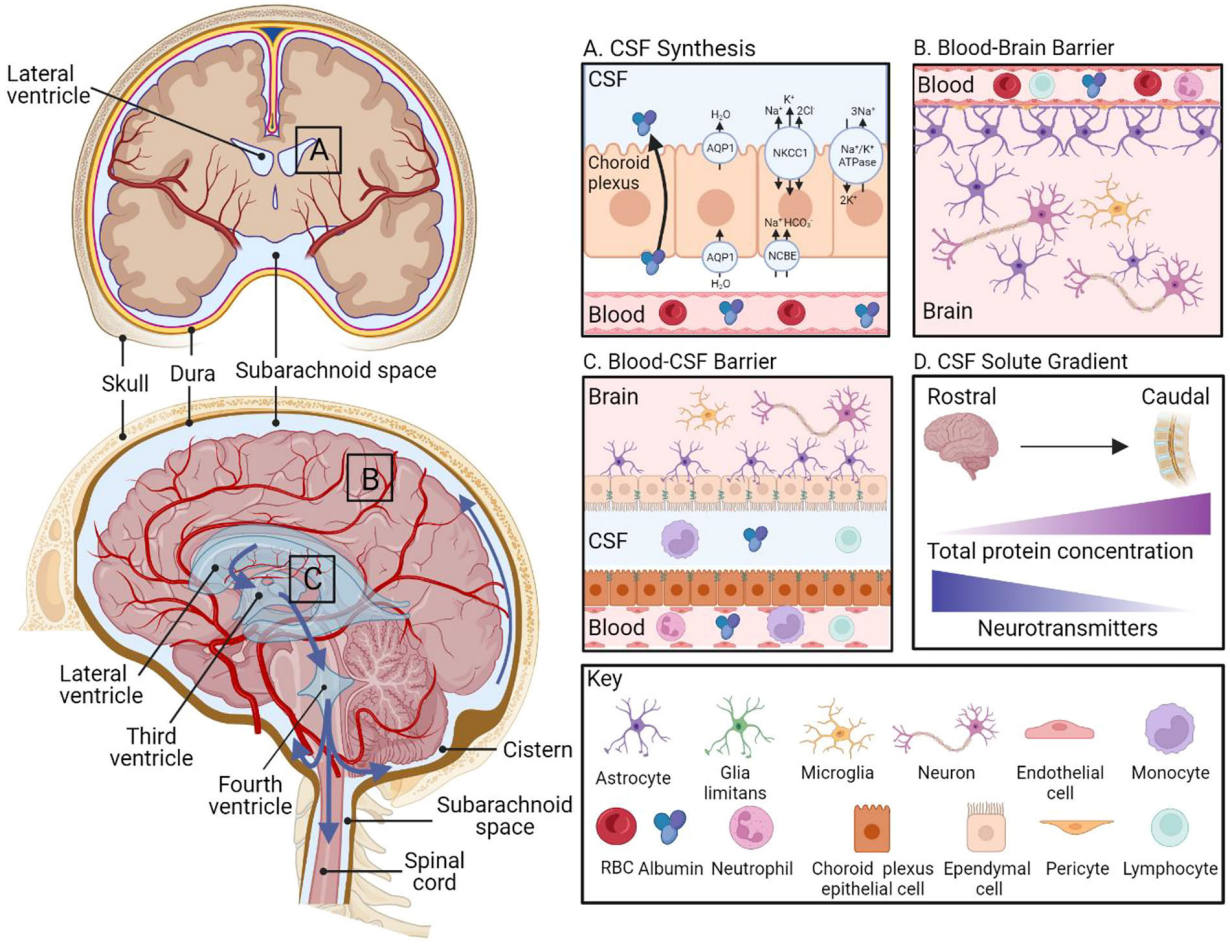
CSF physiology and function. (a, top left) CSF flows through the ventricular system and into the subarachnoid space. (a, top right) CSF is produced within the choroid plexus, a pia mater‐derived network of fenestrated blood capillaries within each of the ventricles. AQP1 = aquaporin 1; NCBE = sodium bicarbonate transporter; NKCC1 = sodium–potassium‐chloride cotransporter 1; NHE = sodium–hydrogen exchanger. (b, right) The BBB is formed by the endothelial cells and connected by tight junctions, pericytes, and end‐feet processes of specialised astrocytes. (c, right) The blood‐CSF barrier is formed by capillaries with fenestrated endothelium and epithelial cells in the choroid plexus. The apical region of the choroid plexus epithelium and ependymal cells are connected via tight junctions, both in direct contact with CSF. CSF contains lymphocytes and monocytes involved in CNS immune surveillance. (d) CSF solute gradient, from rostral structures (i.e., lateral ventricles) to caudal lumbar CSF. Total protein concentration exhibits a caudal‐rostral gradient (highest levels in the caudal CSF), while there is a rostro‐caudal gradient for some neurotransmitters like Gamma‐aminobutyric acid (greatest levels in the rostral CSF). Created with BioRender.com.

CSF is a clear, transparent fluid derived from blood plasma that provides physical support for the brain, collects waste, circulates nutrients and extracellular components to remote sites in the brain, lubricates the central nervous system (CNS) and provides mechanical protection, metabolic homeostasis and immunological surveillance (for a comprehensive review, see Tumani et al., 2018). CSF is produced in the ventricular system, which is comprised of four interconnected brain regions containing the choroid plexus, a specialised tissue involved in CSF production (Figure 1, left). The choroid plexus filters circulating plasma to form an ultrafiltrate (Wichmann et al., 2021). From this ultrafiltrate, the choroid plexus epithelial cells pump ions into the CSF (Karimy et al., 2016), creating an osmotic gradient that allows water entry via water channel proteins called aquaporins (Figure 1a) (Brinker et al., 2014; Del Bigio, 2010).

Adult humans produce approximately 500 mL of CSF per day (Brown et al., 2004), with ∼90 to 150 mL present at any one time (Sakka et al., 2011), and total CSF volume is replaced every 5–7 h. To maintain CSF circulation at a constant volume, absorption occurs at the same rate as production under physiological conditions (Khasawneh et al., 2018). However, CSF flow and composition can be affected by several conditions, such as neuroinflammatory and neurodegenerative diseases (Simon & Iliff, 2016). In adult mammals, the CNS contains two barriers that prevent the free passage of plasma components into the brain or the CSF: (i) the blood‐brain barrier (BBB), and (ii) the blood‐CSF barrier (Johanson et al., 2008) (Figure 1b,c). The BBB is formed by brain microvascular endothelial cells, pericytes and astrocyte end‐feet that regulate the flow of substances into and out of the brain (Lippmann et al., 2012). While most cells and proteins in CSF are derived from plasma, other solutes (sodium, chloride, etc.) can be actively transported by the endothelial cells into the CSF (Ueno, 2007). Under normal physiological conditions, plasma proteins enter the CSF by transcytosis across capillary endothelial cells (Broadwell, 1992). However, endothelial cell permeability can change in pathological states, leading to increased permeability and/or alterations in CSF composition. Additionally, neuronal damage (Tarawneh et al., 2015), astroglial and microglial activation in pathological states (Dotevall et al., 1999; Masvekar et al., 2019), and brain infiltrating cells (D'Asti et al., 2016; Esaulova et al., 2020) can alter the concentration of cells and proteins in the CSF.

### 1.4 Clinical use and limitations of CSF biomarkers

Lumbar puncture (LP) for CSF analysis is an integral aspect of current clinical diagnoses in a variety of neurological diseases and disorders. The diagnosis of microbial infections includes CSF cell counts, glucose and protein measurements, in combination with CSF culture and PCR testing for specific infectious agents (Shahan et al., 2021; Solomon, 2020). CSF IgG studies are used for the diagnosis of multiple sclerosis and other neuroimmune disorders (Freedman et al., 2005; Giovannoni, 2014). More specific CSF analyses have been developed for the evaluation of neurodegenerative diseases. For example, CSF measurements of amyloid beta peptide (Aβ)_1‐42_, microtubule associated protein tau, and phosphorylated forms of tau (p‐tau) are used to aid in the diagnosis of Alzheimer's disease (AD) (Blennow et al., 2015, 1995; Hansson et al., 2019; Maddalena et al., 2003). Diagnosis of Creutzfeldt‐Jakob disease utilises the presence of a normal intracellular protein (14‐3‐3 gamma) that spills into the CSF during rapid neurodegeneration (Hsich et al., 1996; Muayqil et al., 2012) or the aggregated form of prion proteins (Atarashi et al., 2011; McGuire et al., 2012). A novel real‐time quaking‐induced conversion (RT‐QuIC) assay for aggregated forms of α‐synuclein has been developed and recently commercialised for the potential diagnosis of Parkinson's disease (PD) and other synucleinopathies (Fairfoul et al., 2016; Okuzumi et al., 2021).

While CSF markers are important paraclinical parameters to aid in the diagnosis of CNS diseases, there are important limitations with current CSF biomarkers that warrant further consideration. More precisely, different biomarkers may be needed for diagnosis, prognosis, progression monitoring and checking response to therapies. Also, single molecules or biomarkers do not fully reflect all aspects of pathophysiology. The use of CSF Aβ_1‐42_ and p‐tau_181_ in AD clinical trials is an illustrative case. Although a framework for incorporating CSF Aβ_1‐42_ and p‐tau_181_ has been proposed to standardise AD clinical trial enrollment and to monitor therapeutic response (Cummings, 2019; Jack et al., 2018), studies show that CSF levels of Aβ_1‐42_ and p‐tau_181_ plateau during clinical disease progression and may not be effective at monitoring late‐stage AD (Bridel et al., 2022; Jack et al., 2013; Mattsson et al., 2018; Palmqvist et al., 2019). Specific and possibly non‐overlapping markers may be needed for diagnosis, prognosis, disease monitoring, and assessment of response to therapies.

### 1.5 CSF extracellular vesicles in healthy and disease states

**FIGURE 2:**
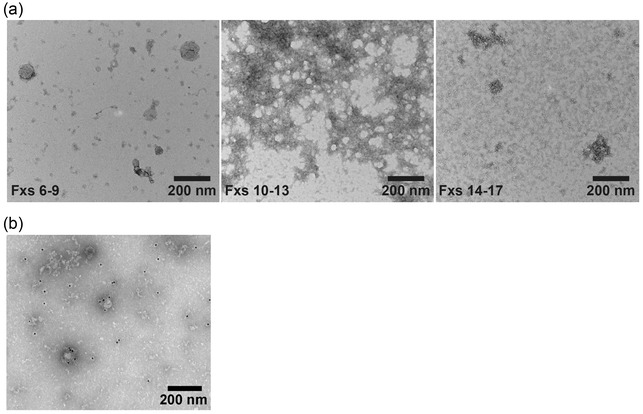
EV and Nanoparticle Composition of CSF. (a) Pooled human CSF from neurologically normal male and female living donors was fractionated by SEC and processed for TEM using a uranyl acetate negative staining protocol, according to methods in Li et al. (2017). Pooled CSF SEC fractions (Fxs) 6–9 (left) show particles that are of the expected size range of EVs (∼50–150 nm) and smaller nanoparticles (<50 nm), Fxs 10–13 (middle) and Fxs 14–17 (right) show an abundance of lipoproteins and proteins in CSF. (b). TEM showing immunogold labeling of human CSF EVs isolated with anti‐CD9 antibody conjugated with 10 nm immunogold. CSF‐derived EVs were separated with an affinity capture kit according to methods in Muraoka, Lin et al. (2020). Scale bars 200 nm.

Due to its physical contact with both the brain and the spinal cord and the clinical ability to sample it, CSF is thought to be highly informative about the state of the CNS in health and disease (Bruschi et al., 2021; Janelidze et al., 2018; Jeromin & Bowser, 2017), and CNS EVs can be found in CSF (Hjalmarsson et al., 2014; Li et al., 2021). CSF EVs were first visualised in human, mouse and sheep CSF by electron microscopy (EM) (Bachy et al., 2008; Ekelund et al., 2003; Harrington et al., 2009; Huang et al., 2009; Marzesco et al., 2005; Morel et al., 2008; Vella et al., 2008; Wetterberg et al., 2002). Healthy adult human CSF has been reported to contain ∼2e8–7e9/mL nanoparticles (e.g., EVs, lipoproteins) based on nanoparticle tracking analysis (NTA) measurements (Hong et al., 2021; Kuharić et al., 2019; Lee et al., 2020; Tietje et al., 2014) One of these studies also reported an age‐dependent decline of CSF nanoparticles, with ∼5e10/mL in children <2 years old, ∼2e10/mL in children 10–15 years old, and ∼7e9/mL in adults >70 years old (Tietje et al., 2014). Transmission EM (TEM) images of a pool of healthy human CSF fractionated by size exclusion chromatography (SEC) demonstrated a diversity of particle types. Early fractions (generally larger particles) included a heterogeneous composition of particles <50 nm and, to a lesser extent, of EVs that are 50–150 nm (Figure 2a left, Fxs 6–9), while later SEC fractions (smaller particles) contained an abundance of lipoproteins (Figure 2a middle, Fxs 10–13) and proteins (Figure 2a right, Fxs 14–17). TEM also showed immunogold labelling of CD9 on human CSF EVs (Figure 2b) separated with an affinity capture kit (Muraoka, Lin, et al., 2020). High‐resolution mass spectrometry (MS) of EVs separated by ultracentrifugation (UC) from 6 mL of CSF pooled from healthy individuals identified 1315 proteins, 30% of which were quantitatively enriched in the EVs (pelleted fraction) versus the UC supernatant (Chiasserini et al., 2014). Proteins enriched in the CSF EVs included Alix, syntenin‐1, tetraspanins (CD9, CD81), heat shock proteins A8 and 90AB1, RAB proteins, eukaryotic translation elongation factor 1A1 and 2, and major histocompatibility complex antigens (Chiasserini et al., 2014). Multiple species of RNAs, including microRNA (miRNA) and messenger RNA (mRNA), have also been identified in CSF EVs (Patz et al., 2013; Saugstad et al., 2017; Yagi et al., 2017).

Tissue expression mapping shows that CSF EVs are highly enriched for brain‐derived proteins, including microglial and neuronal markers (Chiasserini et al., 2014), and half of brain miRNAs are reported to be in CSF EVs (Yagi et al., 2017). To further elucidate the tissue and CNS origin of CSF EVs, one study compared microarray data from the Allen Brain Atlas (Hawrylycz et al., 2012) against CSF EV and total CSF datasets (Thompson et al., 2018) and found that the EV‐enriched proteome better reflects the choroid plexus and white matter proteome than total CSF (Thompson et al., 2018). Other cellular sources of CSF EVs include neurons, microglia, oligodendrocytes (Chiasserini et al., 2014; Geraci et al., 2018; Guha et al., 2019; Muraoka et al., 2019; Muraoka, Jedrychowski, et al., 2020; Verderio et al., 2012) and stem cells (Feliciano et al., 2014; Huttner et al., 2012, 2008; Lepko et al., 2019), but the contribution of each cell type to the composition of healthy CSF EVs has yet to be determined.

Many single site studies have used CSF EVs to identify candidate biomarkers for diseases, including autoimmune encephalitis (Li et al., 2022), Huntington's disease (Sanchez et al., 2021), meningeal carcinomatosis (Cheng et al., 2021) and multiple sclerosis (Pieragostino et al., 2018). One study showed that the transfer of genetic material from CSF microparticles to adult neuronal stem cells in vitro resulted in a subsequent miRNA‐specific repression of distinct genes (Patz et al., 2013). There is also evidence that microglial small EVs facilitate and contribute to alpha‐synuclein transmission in PD (Crotti et al., 2019; Guo et al., 2020), highlighting potential EV roles in disease pathophysiology. Other pathological functions of CSF EVs include inflammatory responses mediated by choroid plexus derived‐EVs, which contribute to cognitive decline in two mouse models of AD (Vandendriessche et al., 2021) and induce microgliosis and astrogliosis in the Niemann‐Pick's disease mouse model of neurodegeneration (Van Hoecke et al., 2021). In glioblastoma patients, CSF EVs are implicated in immune evasion and immunosuppressive mechanisms (Wang et al., 2020). CSF EVs also play an active role in the pathogenesis of CNS infections. For example, Human Immunodeficiency Virus‐1 (HIV‐1) uses endosomal complexes required for transport (ESCRT) for viral production, exploits proteins that regulate EV biogenesis for virion production (Rodrigues et al., 2018), and can mediate viral‐associated neurodegeneration (Kutchy et al., 2020). EVs from HIV‐1‐infected microglia carry HIV‐Negative Regulatory Factor (Nef) protein, which increases BBB permeability by reducing expression of the tight junction protein 1 (Raymond et al., 2016). Nef‐carrying EVs also induce toll like receptor 4‐mediated expression of cytokines and chemokines in microglia (Raymond et al., 2016). Moreover, CSF EVs derived from neurons, glia, and choroid plexus epithelial cells of HIV+ individuals with cognitive impairment carry synaptic, inflammation‐related, and stress response proteins, supporting their potential as biomarkers for and conveyors of HIV‐associated neurocognitive disorders (Guha et al., 2019). EVs are been implicated in fungal and parasitic infections, such as in *Naegleria fowleri* amoebic encephalitis (Retana Moreira et al., 2022), which has a high mortality rate of ≥95% and rapid progression to death within 3–7 days after symptoms appear. Given a lack of specific biomarkers for this fatal protozoan infection, CSF EVs could serve the critical unmet need for early diagnosis of this infection.

These studies underscore the role of EVs in maintaining CNS homeostasis in health, and as mediators of and biomarkers for CNS disease states. Therefore, in order to leverage CSF EVs to study CNS health and disease, reproducible CSF EV studies are needed. The general recommendations for CSF EV studies are to adhere to MISEV2018 guidelines and checklists where feasible (Théry et al., 2019), and to reposit all experimental parameters into EV‐TRACK (https://evtrack.org/, Van Deun et al., 2017). To promote the reproducibility of CSF EV studies specifically as this field advances, here, we offer recommendations and reporting guidelines that are highly relevant and/or unique to CSF EV studies. For a summary of all recommendations and reporting guidelines, see Table 1.

**TABLE 1:** Recommendations and reporting guidelines for CSF EV studies.

Basic information	Details to report
**General recommendations**	
MISEV2018	Adhere to MISEV2018 guidelines and checklist where feasible
EV‐TRACK	Reposit experimental parameters
**CSF collection, processing, and storage**	
Clinic/Hospital site	Organisation; City; State/Country
Patient information	Sex; Age; Medications; Pre/Post mortem; Disease state(s)
Collection site	Lumbar; Cranial; Intracranial
Collection type	LP; External ventricular drain; Ventricular drain; Shunt; Post‐mortem
Sedation	Yes vs. No; Anesthetic type
Collection time	AM vs. PM
Fasting	Time since last meal
Collection device	Needle type (e.g., 24 or 25 g Sprotte needle)
Collection Container	Tube type (e.g., Polypropylene tubes)
Collection details	Number of collection tubes; Volume/collection tube (mL); Total volume collected (mL); Collection temperature (e.g., ice); Collection tubes mixed or processed individually (gradient effect)
CSF additives	Type (e.g., Phosphatase inhibitors; Protease inhibitors)
Centrifugation	Speed (g force or relative centrifugal force); Time; Temperature
Ultrafiltration	Type of device; Filter size
Quality control	Blood contamination, Visual macroscopic appearance (e.g., blood); Clinical CSF biochemistry
Exclusion criteria	Blood contamination (e.g., >500 erythrocytes/μL)
Aliquots	Total number of aliquots; Aliquot volume; Labels (e.g., collection date)
Storage device	Tube type (e.g., Cryogenic tubes)
Freezing method	Liquid nitrogen; Dry ice
Storage time	Time from collection to storage
Storage details	Temperature (−20°C, −80°C); Years in storage
**CSF EV separation/Concentration**	
CSF aliquots	Syringe draw/collection tube number for gradient effects
CSF sample details	Individual donor CSF vs. pool of donors CSF
Pooled CSF	Number of individual samples; Volume per sample; Patient information
Fractionation	Report all methodological details pertaining to the fractionation of CSF according to MISEV2018
Volumes	Volume (mL) of CSF used for separation; Final CSF EV volume
Controls	Report the positive and negative controls used to assess the purity of CSF EV prep
Freeze/Thaw	Methodologies used to thaw and freeze CSF/CSF EVs; Number of times CSF/CSF EVs were thawed prior to use
CSF EV storage	Tube type; Temperature (20–25°C, 4°C, −20°C, −80°C); Buffer; Volume; Time in storage
CSF EV stability	Assays and results to determine stability in storage
**CSF EV analysis—General recommendations**	
CSF samples	Report whether individual or pooled CSF samples were assayed; If pools were used report number in individual samples, volume per sample, patient information; Report if validations were performed on individual samples
Volumes	Report the CSF and/or CSF EV volumes used in assays
Limits of detection	Report the sensitivity and detection limits of the assays. Know the sensitivity of the methods and adjust the conclusions accordingly.
**CSF EV size and number**	
MIFlowCyt	Reporting guidelines for FC based analysis of EVs
Calibrator/Standard	Calibrators/standards used for determining sensitivity and detection limits
Controls	Dilution series; Detergent treatment; Method specificity for CSF EV vs. non‐EV components
**CSF EV protein analysis**	
Antibodies	Antibody source, clone, and dilution; Validation assays for specificity
Controls	CSF EV vs. non‐EV markers; Tissue‐ and cell‐type specificity of CNS EV markers
Nomenclature	Refer to a population of EVs as positive for a maker as opposed to cell‐type derived (i.e., EVs+ for L1CAM opposed to Neuronal‐derived EVs)
**CSF EV RNA analysis**	
Blood contamination	Perform CSF hemoglobin assays and report data as blood change the RNA profile
RNA assays	Types of kits used (e.g., RNA isolation, PCR, library preparation, hybridisation)
Modified protocols	Modifications to kit protocols
RNA input	Equal volume vs. concentration of CSF EV RNA used
Normalisation	Methods used to normalise CSF EV RNA data
RNA‐seq	Report CSF EV RNA‐seq data mapping percentages and the number of genes
Hybridisation	Report additional methods and data used to validate hybridisation data
**CSF EV omic analysis**	
Glycomics controls	Validate and report lectin and carbohydrate antibody specificity with negative controls (e.g., competitive sugar, glycosidases)
Glycan standards	Inclusion of glycan standards is required to validate analytical procedures, comparison across studies, and quantification
EV separation for lipidomics	Use EV separation protocols that minimize vesicle‐free lipoprotein contamination and report all methodological details
Lipid extraction	Report all methodological details pertaining to lipid extraction (e.g., method, temperature, mixing times, additives—cooling solvents, antioxidants)
Lipidomics normalisation	We recommend to normalising lipidomics data using internal spike in lipid standards or relative to lipid category, lipid class or CSF EV/particle number
Metabolomics	For seminal CSF EV studies comparison of different extraction methodologies and chromatographic parameters as well as inclusion of complementary analysis techniques (e.g., MS‐based and nuclear magnetic resonance) is recommended

## 2 CSF COLLECTION, PROCESSING AND STORAGE

### 2.1 Current reporting of CSF collection, processing and storage

Respondents to the CSF Task Force survey indicated that the majority of studies have been done using human CSF (34 respondents, 83%), followed by rat (4 respondents, 10%), mouse (2 respondents, 5%) and (non‐human primate (1 respondent, 2%). The literature indicated that in addition to these species, CSF from chickens, horses, pigs and sheep have also been reported (Table S2. CSF Collection & EV Separation/Concentration). In addition, based on the literature, most CSF EV studies have focused on brain cancers and injuries, as well as neurodegenerative diseases. Of the 136 human CSF EV studies included in our literature review study, 29 (21%) reported details of the sample collection, such as the collection site (e.g., LP) and the collection volume. Twenty (14%) of these 136 CSF EV studies also reported the time of day of CSF collection, with morning (AM) being the most common (17 of 20, 85%); eight (6%) of the 136 studies reported that CSF was collected under fasting conditions. The needle gauge and/or type was reported in 8 (6%) of the publications. Thirteen studies (10%) performed routine clinical CSF biochemistry to assess cell counts, glucose, total protein and blood contamination. Thirteen studies (10%) outlined exclusion criteria based on blood contamination (e.g., erythrocytes/μL; total cell counts/L; abnormal cell, protein, or red blood cell counts; CSF with visible macroscopic blood). Thirty‐nine (29%) studies reported the collection tube type, and 53 (39%) studies described CSF processing details (i.e., centrifugation or ultrafiltration (UF) before CSF storage). Ninety‐two (67%) reported the freezing method for CSF, with the most common being snap freezing on dry ice or liquid nitrogen, and long‐term storage at −80°C. Furthermore, of the total 161 CSF EV articles, 25 (15%) reposited their experimental parameters into the EV‐TRACK platform (Van Deun et al., 2017). The fact that few publications comprehensively reported pre‐analytical variables regarding CSF collection, processing and storage presents a major challenge to reproducibility and comparing outcomes individual laboratories.

### 2.2 Current best practices for CSF collection, processing and storage

Differences in pre‐analytical variables affect biomarker measurements in total CSF by up to 20%–30% (Klener et al., 2014; Lewczuk et al., 2006; Mattsson et al., 2011). The CSF collection site and collection volume can affect biomarker expression levels (Brandner et al., 2014; Cameron et al., 2019; Reiber, 2003; Teunissen et al., 2009) because a CSF composition gradient exists between the ventricles, cisterns and subarachnoid space (Khan et al., 2019). Unidirectional flow establishes a rostro‐caudal gradient (Aasebø et al., 2014) that results in lower concentrations of brain proteins such as S‐100β, total Tau, and phosphorylated Tau in the lumbar region than in the cisternal region, depending on the type of neurological disorder, if present (Brandner et al., 2014; Reiber, 2003). Conversely, total protein concentration is increased in the lumbar region compared to the lateral ventricles (Weisner & Bernhardt, 1978) (Figure 1d). There are also greater amounts of RNA in CSF in cranial samples from patients with subarachnoid haemorrhage or glioblastoma, which is likely attributable to blood contamination in the total CSF versus that in lumbar CSF (Saugstad et al., 2017). Accordingly, 90% of patients with subarachnoid haemorrhage have red blood cell lysis and bilirubin in the CSF (UK National External Quality Assessment Scheme for Immunochemistry Working Group, 2003). CSF protein levels can also be impacted by neurological diseases, brain injury, acute demyelinating/inflammatory diseases such as Multiple Sclerosis and Guillain Barre Syndrome (Hrishi & Sethuraman, 2019), and traumatic LP. Other factors that can affect CSF composition include impaired CSF homeostasis, increased permeability of the BBB, and changes in cellular trafficking into and out of the CNS. Biological variables that can impact total CSF content include age (Li et al., 2017; Shah et al., 2011; Sjögren et al., 2001; Statz & Felgenhauer, 1983; Vågberg et al., 2015; Wong et al., 2000; Zhang et al., 2005), sex (Li et al., 2017; Sandau et al., 2022), genetics (Li et al., 2017; Sandau et al., 2022), ethnicity (Howell et al., 2017) and medical/social history (e.g., prescription medications, smoking status, alcohol use) (Liu et al., 2020; Riekse et al., 2006; Wang et al., 2021; Wong, 2007). CSF composition can also be affected by increasing age, which is associated with decreased CSF opening pressure (Wang et al., 2019; Whiteley et al., 2006). Low CSF opening pressure (i.e., >15–30 mmHg) can be increased by changing the patient's position during the LP, or by any process that disrupts the normal balance of CSF secretion and absorption. Thus, it is important to consider any factors that may affect CSF composition when designing experiments and interpreting data, and to report these variables along with results.

### 2.3 Recommendations and reporting guidelines for collection, processing, and storage

To improve the reproducibility of CSF EV studies, the CSF Task Force has compiled minimum reporting guidelines for CSF collection, processing, and storage (Text Box 1; Table 1), and recommend use of a standardised and detailed CSF sample collection form for records and reporting, such as the example in Supplemental Form 1. It is recommended to measure blood contamination, exclude samples that exceed pre‐established thresholds, and report data and exclusion criteria. One recommendation for CSF studies is to exclude samples that contain >500 erythrocytes/μL (Teunissen et al., 2009). Additional markers for blood contamination include haemoglobin, catalase, peroxiredoxin, carbonic anhydrase I, apolipoprotein B‐100, IgM, fibrinogen and haptoglobin (Aasebø et al., 2014; You et al., 2005). At a minimum report the visual appearance of each CSF sample (e.g., clear, turbid, reddish). In addition, report the use and type of anaesthetics, as these may be associated with altered miRNA expression and EV function (Abel et al., 2020; Buschmann et al., 2020; Dong et al., 2021). The Task Force acknowledges that previously banked CSF may not be associated with complete patient/donor demographic data and/or methodological details of collection, processing and storage of CSF. In such cases, it should be indicated which details are not available. As for which pre‐analytical variables should be reported, some may be specific to CSF. However, others may be known to impact EVs from other biofluids, such as using fresh versus frozen samples, time and holding temperature between collection, pre‐processing and storage, centrifugation prior to freezing, freeze/thaw cycles and additives (Cameron et al., 2019).

**Text Box 1. Recommendations and Reporting Guidelines for CSF Collection, Processing and Storage**

**Recommendations**

- Use a standardised and detailed sample collection form, such as Supplemental Form 1.
- Collect CSF and briefly centrifuge at 2000 × *g* to remove cells.
- To minimise any gradient effects of lumbar collection, CSF can be pooled across multiple syringes prior to aliquoting.
- If not used fresh, store pre‐processed CSF at −80°C as soon as possible (Saugstad et al., 2017; Teunissen et al., 2009).
- To avoid multiple freeze/thaw cycles, aliquot CSF in volumes appropriate for downstream assays.

**Reporting Guidelines**

- The CSF Task Force survey respondents indicated that these data must be reported:
- Pre‐collection and collection parameters, if known:
  - Institute or clinical study site where participants donated CSF
  - Institutional Review Board approval number
  - Collection site (e.g., lumbar vs. central)
  - Collection type (e.g., post‐mortem)
  - Collection time of day
  - Fasting or non‐fasting conditions
  - Use and type of anaesthetics
  - Needle type and gauge
  - Type/supplier of collection tube
  - Total volume of CSF collected
- Patient characteristics, if known:
  - Neurological disorder/infection
  - Neuropathology
  - Age
  - Sex
  - Medications
  - Smoking status
  - Vitals (blood pressure, body temperature, etc.)
  - Body mass index
- Handling and storage conditions:
  - Use of additive (e.g., protease inhibitors)
  - Centrifugation speed (in × *g*) and time
  - If CSF was pooled before aliquoting
  - Storage tube type
  - Holding temperature prior to freezing (ice, 4°C, etc.)
  - Time between collection and storing
  - If using fresh CSF, report time between collection and use (hours to overnight, etc.)
  - Freeze/thaw methods (e.g., snap‐freeze on dry ice; thaw on wet ice)
  - Freeze/thaw cycles
  - Long‐ and/or short‐term storage temperature(s)
  - Length of time in storage
- For blood contamination report the exclusion criteria and results of the measurement. At a minimum report the visual appearance of each CSF sample (e.g., clear, turbid, reddish).

## 3 CSF EV SEPARATION/CONCENTRATION

### 3.1 Prevalence of CFS EV separation/concentration methods

A number of techniques have been used to separate CSF EVs from non‐vesicular components based on size, density, and/or biochemical composition (Table 2). Each technique has trade‐offs of yield and purity of the EV preparation (Dong et al., 2020). Examples of separation techniques with high yield but low purity include precipitation and ultrafiltration (UF) with low molecular weight cutoff centrifugal filters (Furi et al., 2017; Karttunen et al., 2018; Stranska et al., 2018; Ter‐Ovanesyan et al., 2021; Théry et al., 2018). Intermediate yield and purity can be achieved with SEC or differential UC (Böing et al., 2014; Furi et al., 2017; Huang et al., 2020; Stranska et al., 2018; Takov et al., 2019; Ter‐Ovanesyan et al., 2021; Théry et al., 2018), while density gradient UC and immunoisolation can result in higher purity but generally lower yield (Furi et al., 2017; Huang et al., 2020; Jeppesen et al., 2019; Théry et al., 2018; Yuana et al., 2014). Combinations of isolation methods can increase the purity of EV preparations (Huang et al., 2020; Visan et al., 2022; Zhang et al., 2020).

**TABLE 2:** CSF EV separation and concentration summary.

Technique		CSF range (mL)	Size & number	Protein Cargo	RNA Cargo	Other assay	Papers (*n*)
**Affinity**	exoEasy Maxi, exoRNeasy Serum/Plasma, ExoIntact	0.5–15	EM, NTA	Immunoblot, ImmunoEM	RT‐qPCR, Sequencing		12
**Centrifugation**	Centrifugation ≤20k g	0.1		FC, ImmunoEM		Functional	3
	Differential Centrifugation ≤25k *g*	0.12–1	EM	FC, IF, ImmunoEM			5
	UC 100k–200k *g*	0.1–8	CryoEM, EM, NTA	FC, Immunoblot, IF, Proteomics, Ultrasensitive ELISA	Digital droplet PCR, Digital PCR, EV‐BEAMing PCR, RT‐qPCR, Sequencing	Functional	16
	dUC ≤200k *g*	0.3–50	CryoEM, DLS, EM, NTA, RPS	Acetyl‐CoA activity, Electro‐chemiluminescence immunoassay, ELISA, FACS, FC, Immunoblot, ImmunoEM, Proteomics, Ultrasensitive ELISA, SP‐IRIS, STED	RT‐qPCR, Hybridisation, Sequencing	Functional	43
	Density Gradient, dUC Sucrose/OptiPrep 70k–200k *g*	0.5–12	EM, NTA	ELISA, FACS, Immunoblot, ImmunoEM, Proteomics, Ultrasensitive ELISA, SP‐IRIS	RT‐qPCR	Functional	11
**Combinations**	SEC + UF	0.5–8	DLS, EM, NTA, RPS	ELISA, FC, Immunoblot, ImmunoEM, Proteomics, SP‐IRIS	RT‐qPCR	Functional, Glycomics, Lipidomics	7
	SEC + Precipitation		EM	Immunoblot	RT‐qPCR, Sequencing		1
	Precipitation + Immunoisolation	0.5–0.7	NTA	ELISA, Immunoblot			1
	Immunoisolation + UF	0.6–5	EM, NTA	Immunoblot, ImmunoEM, ELISA, PEA, Proteomics, Ultrasensitive ELISA		Functional	5
	Immunoisolation + Affinity	0.15		FC			1
	dUC + UF	1–3.5	EM, FACs, NTA	ELISA, FACS, Immunoblot, ImmunoEM, Proteomics	RT‐qPCR	Functional	5
	dUC + Immunoisolation		EM	FC, Immunoblot		Functional	1
	Affinity + UF	0.38		PEA			1
	Precipitation + UF	0.3–0.4	EM, NTA	Immunoblot, Proteomics			1
**Immunoisolation**	L1CAM, PS Capture Exosome FC Kit, CD63, ExoNANO (CD63), CD63 depletion, EasySep Release PE Positive Kit (CD11b)	0.3–5	DLS, EM, NTA, ExoNANO	ELISA, FACS, Immunoblot, ImmunoEM, Proteomics, Ultrasensitive ELISA	RT‐qPCR	Functional	6
**Precipitation**	miRCURY Exosome Cell/Urine/CSF, ExoQuick‐TC, Total Exosome Isolation Kits, Minute Hi‐Efficiency Exosome Isolation Reagent, Ribo Exosome Isolation Reagent, Nanotrap particles	0.1–7	CryoEM, DLS, EM, NTA, Zeta potential	Acetyl‐CoA activity, Dot blot, ELISA, FC, Immunoblot, ImmunoEM, Proteomics, Ultrasensitive ELISA, TiN‐NH‐LSPR biosensor	Digital droplet PCR, Digital PCR, Hybridisation, RT‐qPCR, Sequencing	Neopterin, Functional	44
**Size**	Sequential UF	3	EM, NTA	Proteomics	RT‐qPCR	Functional	2
	SEC	0.5–5	AFM, CryoEM, EM, NTA, RPS, Zeta Potential	ELISA, Immunoblot, PEA, Ultrasensitive ELISA, Slot blot, SOMAscan	RT‐qPCR, Sequencing		7

For Publication PMIDs see Supplemental Tables.

Abbreviations: AFM, atomic force microscopy; CryoEM, cryogenic electron microscopy; dUC, differential ultracentrifugation; DLS, dynamic light scatter; EM, electron microscopy; ELISA, enzyme‐linked immunosorbent assay; FC, flow cytometry; FACS, fluorescence‐activated cell sorting; IF, immunofluorescence; NTA, nanoparticle tracking analysis; PEA, proximity extension assay; RPS, resistive pulse sensing; RT‐qPCR, reverse transcription‐quantitative polymerase chain reaction; SP‐IRIS, single particle interferometric reflectance imaging sensing; SEC, size exclusion chromatography; STED, stimulated emission depletion microscopy; UC, ultracentrifugation; UF, ultrafiltration.

Respondents to the CSF Task Force survey indicated that the top three standard techniques they use to fractionate CSF include SEC (20 respondents, 26%), UC (16 respondents, 21%) and a combination of two or more methods (14 respondents, 18%). Less used methods include UF (8 respondents, 10%), density gradient dUC (7 respondents, 9%), polymer‐based precipitation (e.g., ExoQuick; 6 respondents, 8%) and immunoisolation/antibody capture (5 respondents, 6%). The literature review identified precipitation‐based kits (44 articles, 27%), dUC (43 articles, 26%), a combination of methods (23 articles, 14%), UC (16 articles, 10%), membrane affinity‐based kits (e.g., exoEasy, 12 articles, 7%), density gradient dUC (11 articles, 7%), a combination of SEC with UF or precipitation (8 articles, 5%) SEC (7 articles, 4%), Immunoisolation/antibody capture (6 articles, 4%) and UF (2 articles, 1%). Differential centrifugation at speeds <20,000 × *g* were also used to fractionate larger CSF EVs (8 articles, 5%). See Table S2 for individual references for to each separation/concentration method.

### 3.2 Current best practices for CSF EV separation/concentration

A major challenge to CSF fractionation is the scarcity of biological materials in this biofluid, including EVs. Protein concentrations in CSF are 200–400 times lower than in blood plasma and serum (You et al., 2005). EV concentrations are more difficult to measure, and estimates can vary widely depending on the sensitivity and specificity of methods used (Akers et al., 2016; Coumans et al., 2017; van der Pol et al., 2014). However, two studies using similar methods suggest that, by vesicle flow cytometry (FC), CSF stained with a non‐specific lipid dye results in ∼1000 fold less nanoparticles in lumbar CSF (∼1e8/mL, (Sandau et al., 2022)) compared with plasma (∼1e11/mL, Sandau et al., 2020). Further, tetraspanin+ EVs are 10,000‐fold less abundant in lumbar CSF (∼1e6/mL, Sandau et al., 2022) than in plasma (∼1e10/mL, Sandau et al., 2020). Another study used NTA to report that nanoparticles are ∼500‐fold less concentrated in ventricular CSF (1e10/mL) than serum (∼5e12/mL) (Chen et al., 2013). Consequently, greater volumes of CSF are necessary to obtain a similar total amount of EVs for downstream analysis. To that end, methods to optimise EV separation from biofluids with a low amount of biological material are needed, particularly considering that limited volumes of CSF (∼0.5–1 mL) are typically available from existing biobanks. The abundance of EVs in CSF may also depend on biological factors: while only low volumes (250–500 μL) of paediatric CSF are typically obtained for research purposes, it is reported that these samples have a higher concentration of EVs than adult CSF (Shah et al., 2011; Statz & Felgenhauer, 1983; Wong et al., 2000; Zhang et al., 2005).

A limited number of studies have systematically compared the efficacy of various separation and concentration methods for use with CSF EVs (Akers et al., 2015; Hayashi et al., 2020; Krušić Alić et al., 2022; Muraoka, Lin, et al., 2020; Sandau et al., 2022; Saugstad et al., 2017; Sjoqvist et al., 2020; Soares Martins et al., 2018; Ter‐Ovanesyan et al., 2021; Thompson et al., 2018; Wang et al., 2019). Among them, UC, SEC and affinity isolation were successfully applied to profiling CSF EVs by liquid chromatography‐mass spectrometry (LC‐MS) proteomics or proximal extension assay protein panels (Muraoka, Lin, et al., 2020; Sjoqvist et al., 2020), while use of a membrane affinity‐based kit did not meet the quality assessment (Sjoqvist et al., 2020). Another study assessed EV separation/concentration from a low (250 μL) CSF input volume using two precipitation kits and one column‐based kit, concluding that all three methods produced a low yield and purity of EVs (Soares Martins et al., 2018). Separation methods aimed to increase EV purity have largely been assessed using higher input volumes of CSF (≥500 μL). In SEC, the resin type and length/volume ratio of the column affects the yield and purity of CSF EVs (Sandau et al., 2022; Ter‐Ovanesyan et al., 2021). The sample volume, flow rate, and particle concentration also impacts separation. One study reported that EV yield is greater for home‐packed columns with smaller resins (CL‐6B, 10–4000 kDa) vs. larger resins (CL‐4B, 60–20,000 kDa; CL‐2B, 70–40,000 kDa), as well as when compared with commercially available 70 nm SEC columns (IZON), UC ± wash, and two polymer‐based precipitation methods (Ter‐Ovanesyan et al., 2021). However, fractionation with the smaller pore size resin results in increased albumin in the later CSF EV‐containing fractions. CSF EV purity is improved by either limiting the number of fractions that are pooled (e.g., not including fractions in which EVs co‐elute with lipoproteins) or increasing the column volume from 10 to 20 mL, albeit with reduced yields of EVs (Ter‐Ovanesyan et al., 2021). Sepharose CL‐6B achieved greater EV yields compared with Sephacryl S‐400 HR, Superose 6 PG and a resin with a 70 nm size pore in a commercially available column (Krušić Alić et al., 2022). One caveat to SEC is that it dilutes input material, which further dilutes low abundance CSF EVs and may limit downstream analysis. To circumvent this issue, SEC has been paired with UF or precipitation‐based methods to concentrate the CSF before and/or after EV collection (Costa et al., 2020; Crotti et al., 2019; Hayashi et al., 2020; Kurzawa‐Akanbi et al., 2021; Lee et al., 2020; Sandau et al., 2022; Thompson et al., 2018; Tietje et al., 2014).

### 3.3 Recommendations and reporting guidelines for CSF EV separation/concentration

While methods to improve CSF EV separation are available, overcoming the low concentration of EVs in CSF is still a limitation to this biofluid. Three solutions for this include: (i) increasing the input volume of CSF, (ii) improving recovery (possibly at the cost of purity) and/or (iii) increasing the sensitivity of the EV characterisation. Concentrating CSF prior to EV separation is also an option to increase input volume; albeit, adding any method reduces yield. Depending on the separation method, the attainable yields of EVs using ≤500 μL CSF input may preclude downstream analytics that require greater inputs of material (e.g., RNA for sequencing vs. qPCR, or protein for immunoblot vs. ultrasensitive ELISA/SIMOA). Studies are also needed to assess the impact of storage parameters (e.g., freeze/thaw cycles, additives, time in storage) on the stability and integrity of CSF EVs and their cargo. It is also important to note that immunoisolation of cell type‐specific EVs from CSF is currently limited, in that many CNS cell‐type EV markers still require validation for cell‐ and tissue‐specificity and assessment of utility in normal and diseased CSF (Hartjes et al., 2020; Hoo et al., 2008; Maguire et al., 2018; Manek et al., 2018; Shao et al., 2018; Sverdlov, 2012; Vacchi et al., 2021). See CSF EV Protein Analysis (Section 5) for a detailed discussion of the current CNS cell type EV markers. In Text Box 2, and Table 1, the Task Force provides recommendations and minimum reporting guidelines for CSF EV separation/concentration.

**Text Box 2. Recommendations and Reporting Guidelines for CSF EV Separation/Concentration**

**Recommendations**

- For new and established protocols, perform purity assessments based on MISEV2018.
- More studies are needed to assess the impact of post‐fractionation storage parameters (e.g., freeze/thaw cycles, additives, time in storage) on CSF EVs and their cargo.
- More studies are needed to assess the impact of biological factors (e.g., age, sex, genetics) and disease on CSF EVs.

**Reporting Guidelines**

- Report methodological details according to the MISEV2018 checklist (Théry et al., 2018), and reposit methods in the EV‐TRACK platform (Van Deun et al., 2017).
- A majority of CSF Task Force survey respondents indicated that the following be reported:

- Assays and results to determine EV stability in storage, if performed
- Storage temperature, buffer composition, and aliquot volume
- Freezing methodology (e.g., −20°C, dry ice, −80°C)
- Freeze/thaw cycles before assays
- Storage duration

• Starting volume of each CSF sample.
• If CSF is pooled, report:

- Number of individual donors used to generate the CSF pool
- Donor demographics of each individual sample
- CSF volumes used of each individual sample
- Final volume of the pooled CSF used for separation and/or EV analysis

• If available, report the CSF syringe draw/collection tube number to account for possible gradient effects of collection.
• Positive and negative controls are particularly important to aid interpretation of low‐sensitivity techniques.
• Report any post‐fractionation parameters that could impact the integrity of CSF EVs stability/integrity (e.g., freeze/thaw cycles, additives).

## 4 CSF EV SIZE AND NUMBER MEASUREMENTS

### 4.1 Current reporting of methods for CSF EV size and number measurements

The CSF Task Force survey respondents indicated the use of both bulk and single particle measurements to characterise the size and/or number of particles in CSF, with the most frequently used analytical methods being NTA (31 respondents, 40%), TEM (16 respondents, 21%) and FC (16 respondents, 21%). Respondents also indicated the use of cryogenic EM (4 respondents, 5%), RPS (4 respondents, 5%), EVQuant assay (Hartjes et al., 2020) (2 respondents, 3%), super resolution microscopy (2 respondents, 3%), atomic force microscopy (1 respondent, 1%) and scanning EM (1 respondent, 1%). The literature review generally reflected the survey results with EM (64 articles, 40%) and NTA (53 articles, 33%) being the most frequently reported methods. However, newer approaches to sizing and counting include super resolution microscopy (1 article, 1%), nanoscale FC/light scatter pulse (5 articles, 3%), and vesicle FC/fluorescence pulse (3 articles, 2%). See Table 3 and Table S2 for individual references for each method.

**TABLE 3:** EV size and number measurements.

Method		% Used	Counting principle	Size, principle	Input volume (μL)	Size range (nm)	Papers (*n*)
**Fluid**	CryoEM	3	Visualisation	Diameter, image analysis	∼50	∼30–500	5
	Nanoscale FC	3	Light scatter pulse	Diameter, from scatter intensity w/standards, Mie theory models	5–500	∼150–2000	5
	NTA	33	Light scatter	Hydrodynamic radius, diffusion	500–1000	∼50–300	53
	RPS	6	Impedance pulse	Volume, impedance pulse amplitude	10–40	∼60–300 ∼50–200 ∼100–2000	9
	Vesicle FC	2	Fluorescence pulse	Surface area, from fluorescence intensity, calibrated w/standards	5–50	∼30–2000	3
**Solid**	EM (SEM, TEM)	40	Visualisation	Diameter, image analysis	∼50	∼30–500	64
	SP‐IRIS	4	Spot counting	Diameter, from scatter intensity w/standards	∼100	∼70–300	6
	dSTORM, STED Imaging	1	Image analysis	Diameter, image analysis	∼50	∼30–500	1
	Wide Field Fluorescence	0	Spot counting	Not applicable	∼100	Not applicable	0

Abbreviations: CryoEM, cryogenic electron microscopy; dSTORM, direct stochastic optical reconstruction microscopy; EM, electron microscopy; FC, flow cytometry; NTA, nanoparticle tracking analysis; RPS, resistive pulse sensing; SEM, scanning electron microscopy; SP‐IRIS, single particle interferometric reflectance imaging sensing; STED, stimulated emission depletion microscopy; TEM, transmission electron microscopy; vFC, vesicle flow cytometry.

### 4.2 Current best practices for CSF EV size and number measurements

The size profile and number concentration of CSF EV subtypes and/or particles may relate to disease states. A relatively consistent finding reported across studies and species is a change in the median CSF EV and/or particle size and concentration with aging by NTA (Tietje et al., 2014), and with diseases including PD by NTA and bead‐based FC (Vacchi et al., 2021), multiple sclerosis by FC (Pieragostino et al., 2018; Verderio et al., 2012), Niemann‐Picks disease by SP‐IRIS (Van Hoecke et al., 2021), herpes simplex encephalitis by NTA (Li et al., 2022), post‐traumatic brain injury by NTA (Kuharić et al., 2019; Manek et al., 2018) and AD by NTA and SP‐IRIS (Vandendriessche et al., 2021). Single‐particle analysis of CSF EVs has been performed with both fractionated and unfractionated CSF (for individual references see Table S2). Single‐particle measurements of EVs captured on a surface can allow direct visualisation of EVs, but introduces uncertainties regarding the relationship of the number and size of the captured EVs relative to those in the original sample. Several recent reviews have discussed technologies for sizing and counting of EVs (Gandham et al., 2020; Rupert et al., 2017; Shao et al., 2018), also summarised in Table 3. In addition, the ISEV‐ISAC‐ISTH Flow Cytometry Intersociety Working Group (ISAC: International Society on Advancement of Cytometry, and ISTH: International Society on Thrombosis and Haemostasis) has published an educational compendium, and recommendations and reporting guidelines for single‐vesicle FC experiments, that are applicable to CSF EV studies (Welsh et al., 2021, 2020, 2023) and relevant to other single‐EV measurement approaches.

### 4.3 Recommendations and reporting guidelines for CSF EV size and number measurements

Methods for counting and sizing of individual EVs are rapidly evolving as limitations of older methods are recognised and new approaches are introduced and validated. Given the heterogeneity of particles in CSF (Akers et al., 2016; Akers et al., 2015; Sandau et al., 2022; Saugstad et al., 2017), single‐particle measurement methods are more useful than bulk measurements, which report only population total or average values. For example, dynamic light scattering measures the bulk optical properties of a sample. Applying mathematical models to dynamic light scattering data, one can estimate the average particle diameter in samples containing monodisperse particle populations. However, these models are challenged by analysis of polydisperse particles like EVs (Hoo et al., 2008; Maguire et al., 2018). In contrast, single‐particle measurements directly count and estimate the size of individual particles, enabling reporting of numbers of particles as a function of size. Single‐particle measurements performed in solution and, in some cases, without CSF fractionation, allow a direct estimation of concentration and size (for references see Table S2). While the total protein concentration of an EV‐containing sample is often used as a surrogate for EV concentration, variations in the composition of EVs in a sample, as well as the presence of soluble proteins and protein particulates make total protein concentration a poor proxy for EV concentration (Sverdlov, 2012). The literature suggests that, similar to other biofluids, CSF contains EVs in the size range of ∼40 to several hundred nm (Akers et al., 2015, 2016; Saugstad et al., 2017) as well as smaller nanoparticles (Sandau et al., 2022). Size distributions can be affected by fractionation aimed at separating EVs relative to other biofluid components, with concentrations of ∼1e6 particles/mL (Sandau et al., 2022). Importantly, measurement outcomes can be dependent on the sensitivity and specificity of the method as well as the CSF collection site and other biological factors (e.g., age) (Akers et al., 2016; Coumans et al., 2017; Saugstad et al., 2017; Tietje et al., 2014; van der Pol et al., 2014). It is also important to note that CSF contains a smaller number of nanoparticles/EVs compared with plasma and serum (Sandau et al., 2020, 2022) which presents a challenge for downstream analysis (Brennan et al., 2020; Muraoka, Lin, et al., 2020). Each of these factors should be taken into consideration when designing experiments, interpreting data, and reporting methods and results. In Text Box 3, and Table 1, the Task Force provides recommendations and the minimum reporting guidelines regarding CSF EV size and number measurements for inclusion in the methods of research publications.

**Text Box 3. Recommendations and Reporting Guidelines for CSF EV Size and Number Measurements**

**Recommendations**

- Use appropriate calibrators and standards so that the detection limits of the counting/sizing methods, which impact the quantities measured, can be reported.
- When possible, use single particle methods in addition to or as opposed to bulk measurements.
- Use of total protein concentration is a poor proxy for EV concentration.
- The specificity of the counting/sizing method for CSF EVs relative to any non‐EV components should be considered, and addressed through the use and reporting of appropriate controls (Welsh et al., 2021, 2020, 2023) including:
  i) Buffer/reagent only
  ii) Titration/dilution
  iii) Detergent treatment

**Reporting Guidelines**

- For single EV FC data, follow the reporting guidelines from the ISEV‐ISAC‐ISTH Flow Cytometry Intersociety Working Group and the MIFlowCyt‐EV framework (Welsh et al., 2021, 2020, 2023).
- Report the limits of detection for the counting/sizing methods used.

## 5 CSF EV PROTEIN STUDIES

### 5.1 Current reporting of CSF EV protein studies

The CSF Task Force survey respondents indicated the use of standard methodologies for assessing CSF EV protein cargo. Categories include total protein quantification (colorimetric (BCA), 14 respondents, 20%; fluorometric based dyes, 4 respondents, 6%), bulk protein identification and measurements (immunoblot, 18 respondents, 26%; MS, 12 respondents, 17%; ELISA, 8 respondents, 12%) and single‐vesicle protein measurements (FC, 13 respondents, 19%). The literature review identified use of these methods as well as methods for bulk protein assessment (e.g., ultrasensitive ELISA) or immuno‐based imaging (e.g., ImmunoEM) of CSF EVs (Table S3. Protein Analysis). See Table 4 for details of each method.

**TABLE 4:** Protein methods.

Method		Principle	Sensitivity and dynamic range	Strengths	Limitations	Comments
**Bulk total protein quantification**	BCA	Colorimetric dye binding	0.5–50 μg/mL	Compatible with large amounts of detergent	Incompatible with reducing reagents or chelators	Equipment commonly available, influenced by (i) # of peptide bonds; (ii) presence of cysteine, cystine, tyrosine, and tryptophan side chains (PMID: 27112398)
	Fluorescence	Fluorometric dye binding	125–5000 μg/mL	Compatible with reducing reagent	Incompatible with large amounts of detergents	Equipment commonly available
**Gel‐based protein detection**	Silver stain	Colorimetric dye binding	0.25–0.5 ng protein/band	Most sensitive for protein detection	Semi‐ quantitative	Equipment commonly available
	Coomassie brilliant blue R‐250	Colorimetric dye binding	∼100 ng protein/band	Standard method for gel‐based protein detection and MS	Semi quantitative	Equipment commonly available
	GelCode	Fluorescent dye binding	5–25 ng	Ready‐to‐stain solutions	Semi‐ quantitative	Equipment commonly available
	SYPRO stain	Negative staining	0.25–8 ng	Dynamic liner range of fluorescent intensity, no destain or timed steps required	Semi‐ quantitative	Equipment commonly available
	Stain‐free gels	Colorimetric dye binding	10–25 ng	No staining necessary, transferrable to blotting membrane	Semi‐ quantitative	Equipment commonly available
**Bulk Protein Identification and Measurement**	Immunoblot	Immuno‐detection of denatured proteins	Dependent on antibody sensitivity and specificity, lower limit ∼2e8/well for CD9 & CD81 (NanoView: Quantifying and Phenotyping EVs)	Established approach	Semi‐quantitative, reagent dependent	Equipment commonly available
	MS	Detection of labelled protein fragments from highly purified samples	∼1 μg/EV sample, 500 attomole/protein, 0.5–500 fmol/protein	Identifies bulk proteins in EV samples	Semi‐ quantitative, limited by high‐abundant protein contamination	Instrumentation and expertise often available in core facilities
	ELISA	Solid phase sandwich immunoassay	Dependent on ELISA sensitivity and specificity	Quantitative, plate‐based format	Reagent dependent	Equipment commonly available, assays commercially available, permeabilization is needed to release intra‐vesicular proteins
	Bead‐based FC	Solid phase sandwich immunoassay with FC detection	Instrument and assay‐ dependent	Sensitivity, multiplexing (antigen co‐expression)	Reagent dependent	Instruments commonly available, assays commercially available
**Single vesicle protein measurement**	ImmunoEM	EM with immunogold detection		Visual, antigen‐specific, multiplexing (antigen co‐expression)	Low throughput, semi‐quantitative	Instruments and expertise often available in core facilities
	FC	Several variants:	Instrument and assay‐dependent	Quantitative, antigen‐specific, multiplexing (antigen co‐expression)	Sensitivity and specificity are instrument and assay‐specific	Instruments and expertise often available in core facilities, assays commercially available, published reporting guidelines
		Light scatter‐based EV detection; Fluorescence‐based EV detection; IF cargo detection	1e4–1e7/μL; 50–2000 nm;>10–10,000 molecules/EV			
	fNTA	IF detection	Instrument, reagent, and assay‐dependent	Antigen‐specific, size estimated by physical measurement	Low throughput, semi‐ quantitative	Instruments and software commercially available, limited validation to date
	SP‐IRIS	Immunoisolation with interferometry and IF detection	Reagent and assay‐dependent	Antigen‐specific, size estimated by physical measurement	Target‐specific, low throughput, semi‐ quantitative	Instruments and software commercially available, limited validation to date
			1e6–1e9 EVs/mL; 50–300 nm			
			NanoView: Quantify & Phenotype EVs			
	Super resolution fluorescent imaging	Immuno‐isolation with IF detection	Instrument, reagent, assay‐dependent	Antigen‐specific, single molecule/ vesicle resolution	Target‐specific, low throughput, semi‐ quantitative	Instruments and software commercially available, limited validation to date
			∼10 EV/field of view; 30–300 nm; ∼20 molecules/EV			

Abbreviations: BCA, bicinchoninic acid; ELISA, enzyme‐linked immunosorbent assay; fNTA, Fluorescence nanoparticle tracking analysis; FC, flow cytometry; ImmunoEM, immunogenic electron microscopy, MS, mass spectrometry; SP‐IRIS, single particle interferometric reflectance imaging sensing.

### 5.2 Current best practices for CSF EV protein studies

Early CSF EV protein studies focused on identifying candidate biomarkers of neurological disease like post‐subarachnoid hemorrhage cerebral vasospasm (Przybycien‐Szymanska et al., 2016) and CNS inflammatory diseases such as multiple sclerosis (Lee et al., 2016; Verderio et al., 2012; Welton et al., 2017). These studies used various techniques to profile patient CSF EVs, including FC (Verderio et al., 2012), MS using isotopically‐tagged peptides (Lee et al., 2016; Przybycien‐Szymanska et al., 2016) and aptamer‐based proteomics arrays (Welton et al., 2017). These studies reinforced the feasibility of isolating human CSF EVs for detailed protein analysis and provided support for the utility of CSF EVs as ‘liquid biopsy’ biomarkers. Research has rapidly expanded in recent years, with studies describing the protein content of human CSF EVs in neurodegenerative disease (Costa et al., 2020; Hayashi et al., 2020; Muraoka, Jedrychowski, et al., 2020; Spitzer et al., 2019; Thompson et al., 2020; Vacchi et al., 2021), traumatic brain injury (Kuharić et al., 2019; Manek et al., 2018; Muraoka et al., 2019; Patz et al., 2013), HIV‐associated neurocognitive disorder (Guha et al., 2019; Guha et al., 2019), acute bilirubin encephalopathy (Tan et al., 2020) and in the developing brain (Coulter et al., 2018). In addition, proteins associated with immune regulation and inflammation are detected in CSF EVs from patients with neurological conditions (Pieragostino et al., 2019; Verderio et al., 2012), as they are associated with alterations in protein homeostatic mechanisms (Hayashi et al., 2020; Muraoka, Jedrychowski, et al., 2020; Thompson et al., 2020; Vacchi et al., 2021). However, there is a need to perform multi‐centre discovery and validation studies for candidate CSF EV protein biomarkers using standardised, efficient methods for CSF collection, EV separation and biomarker detection to enable the development and implementation of clinical assays. Below we provide details for the current best practices for: (i) use of CSF EV and non‐EV protein markers for characterisation; (ii) candidate EV‐associated biomarkers for neurological diseases and (iii) CNS cell type‐specific EV markers.

#### 5.2.1 CSF EV and Non‐EV protein markers

As per MISEV2018 recommendations, protein characterisation of CSF EVs should include markers for both EVs and vesicle‐free proteins/lipoproteins to assess purity (Théry et al., 2018). The CSF Task Force survey respondents reported using EV markers associated with the endolysosomal pathway and exosome biogenesis, including membrane localised tetraspanins (CD9, CD63, CD81) and cytosolic proteins (flotillin‐1, TSG101) (Jeppesen et al., 2019; Théry et al., 2018). Survey respondents also reported using albumin (19 respondents, 46%) and apolipoproteins (ApoA; 14 respondents, 34%, high‐density lipoproteins (4 respondents, 10%) or low‐density lipoproteins (4 respondents (10%) as the predominant markers for vesicle‐free proteins (Jeppesen et al., 2019; Théry et al., 2018). The literature review was in agreement with the survey results for CD9, CD63, CD81, flotillin‐1 and TSG101 as EV markers, and albumin, ApoA1 and ApoA2 as non‐EV markers. In addition, several Vesiclepedia Top 100 EV proteins have been reported as CSF EV cargo, including, for example, Alix, annexin V, and so on (see Table 5 and Table S4; references in Table S3). However, the Vesiclepedia Top 100 EV Proteins also include non‐EV proteins, which may reflect the lack of purity of EV preparations included in this database. For example, immunoEM for Galectin 3 Binding Protein (LGALS3BP) in CSF EV fractions collected by a combination of centrifugation, UF, and SEC showed localisation primarily with small nanoparticles of irregular shapes and heterogeneous size (15–60 nm), and occasionally with CSF EVs (Costa et al., 2020). LGALS3BP was also found to associate with small structures of irregular shape released from HEK 293T cells (Costa et al., 2018) and exomeres from several tumor cell lines (Zhang et al., 2018). In MISEV2018, LGALS3BP and fibronectin 1 are classified as ‘secreted proteins recovered with EVs’ (Théry et al., 2018).

**TABLE 5:** Protein markers.

Proteins in CSF EV preparations	Markers of CNS‐derived EVs	Non‐EV markers in total CSF	Proposed EV and non‐EV markers
**In Vesiclepedia Top 100 EV Proteins** ACTB, ANXA5, CD9, CD63, CD81, FLOT1, *FN1, GAPDH, *LGALS3BP, PDCD6IP, RAB7A, RALA, SDCBP, TSG101 **Biomarkers for neurological disorders** Aβ, ABCA1, APOE, APP, AQP4, BACE1, BIN1, CASP1, CCR3, CCR5, CD4, CD19, CD44, CD200, CDH5, CNTNAP2, CRP, DEFA1, EGF, ENO2, GABBR1, GABBR2, GRIA1, GRIA2, GRIN1, GRIN2A, GRIN2B, HSPA4, IL13, ITGA2B, LAMP1, LAMP2A, LGI1, LRRK2, LTF, MAP1LC3A, MAP1LC3B, MAPT, NEFL, NLRC4, NOC2L, NRIP1, PARK7, PRDX2, PRNP, PROM1, PTPRC, PYCARD, S100A7, S100A9, SNAP25, SNCA, SPTAN1, SQSTM1, SYP, TAT, UCHL1, WT1 **Other proteins in EV preparations** ARF6, ARL2, C4BPA, CD3, CD14, CD64, CHGA, CHGB, CHI3L1, CP, CST3, EEA1, FGB, FLOT2, FOLR1, HMOX1, HNRNPA2B1, HPR, HSP90, ITGB3, KLKB1, NLRP1, NPM1, NRK, PECAM1, PKM, PTEN, PTGS2, RAB11A, SCG3, SEMA4D, SRD5A2, TUBB3, WDR1	**Neurons** ENO2, L1CAM, NCAM1, THY1, SNAP23, SNAP25, STX, SYP, SYT, UCHL1, VAMP **Astrocytes** GFAP, GLUL, HEPACAM SLC1A3 **Microglia** AIF1/IBA1, CD14, CD86, ENG, HLA‐DR, ITGAM, PTPRC, TMEM119 **Oligodendrocytes** MOG, GALC **Choroid plexus** TTR	**Apolipoproteins** **Blood**: ApoA1/2, ApoB **CNS**: ApoE **Proteins and Glycoproteins** **Blood**: ALB, ORM1, SERPINA1, A2M, FG, HP, IgA, IgG, IgM, TF **CNS**: VDAC1 **Cytoplasm**: PLD1 **Endoplasmic reticulum**: CANX **Golgi**: GOLGA2 **Lysosome**: TFRC **Mitochondria**: CYCS, TFAM	**Neurons** ATP1A3 **Astrocytes** ITGA6, LRP1 **Microglia** FTH1, LCP1, KCTD12, P2RY12, TREM2 **Oligodendrocytes** CNP, FTH1, LAMP2, MBP, PLP1 **Non‐EV markers** CLU/ApoJ **Biomarkers for Neurological Disorders** BSG, IGF1R, ICAM1, IGF2R, MMP2, VIM

Proteins Listed by Gene Name (https://www.genecards.org/); *FN1 and LGALS3BP are listed in Vesiclepedia: Top 100 EV Proteins, but are categorised by MISEV2018 at “secreted proteins recovered with EVs”; See Table S3 for PMIDs for Protein Markers.

Several non‐EV markers of blood contamination (e.g., albumin) or for lipoproteins abundant in the CNS (e.g., ApoE) have been used in CSF EV studies (see Table 5 and Table S4; see references in Table S3). However, in vitro studies have shown that astrocyte‐ and neuron‐derived EVs include ApoE (Nikitidou et al., 2017; Zheng et al., 2018), which is increased in secreted EVs following Aβ_42_ treatment (Nikitidou et al., 2017). ApoE is associated with human CSF EV preparations in normal donors (Sandau et al., 2022) and in relapsing remitting multiple sclerosis patients (Welton et al., 2017). However, studies to validate the localisation of proteins in CSF EVs (cargo, corona and/or contaminant) using techniques that resolve single EVs (e.g., immunoEM, Surface‐enhanced Raman spectroscopy (Koster et al., 2021), and super‐resolution microscopy (Frigerio et al., 2022; McNamara et al., 2022)), are needed.

#### 5.2.2 Candidate EV‐associated biomarkers of neurological diseases

Several CSF EV proteins have been identified as candidate biomarkers for neurological disorders (see Table 5 for acronyms, and Table S4 for full protein names). For example, α‐synuclein, a constituent of Lewy bodies which is the pathological hallmark of PD and Lewy body dementia, is found in CSF EVs (Zarranz et al., 2004). Candidate CSF EV biomarkers for AD include Aβ, APP, ApoE, ABCA1, BACE1, BIN1 and tau (see references in Table S3). ApoE is the most important genetic risk factor for sporadic AD (Corder et al., 1993). Aβ and tau are constituents of plaques and neurofibrillary tangles, respectively, and are the pathological hallmarks of AD (Alonso et al., 1996; Dickson et al., 1988). BACE1 is the enzyme that mediates the β‐site cleavage of APP into Aβ (Vassar, 2004), and CSF EVs containing BIN1‐associated tau facilitate tau spreading in the brain in an AD mouse model (Crotti et al., 2019). BIN1 is also a risk gene of late onset AD (Naj et al., 2011). In addition, the integral cell membrane protein ABCA1 is increased in CSF EVs of patients with mild cognitive impairment and AD related dementia (Liu et al., 2021) and risk factor for AD (Holstege et al., 2022). Candidate proteins in CSF EVs may not be associated with specific diseases, but instead represent general alterations in cellular physiology including immune response and inflammation (e.g., caspase 1, de Rivero Vaccari et al., 2016; Raval et al., 2019), cancer (e.g., interleukin 13 epidermal growth factor (Madhankumar et al., 2017)), synaptic changes (e.g., neurofilament light chain (Manek et al., 2018)) and lysosomal and autophagic activities (e.g., lysosomal associated membrane protein 1 (Minakaki et al., 2018)). Table 5 and Table S4 lists all reported candidate CSF EV protein biomarkers (for references see Table S3).

#### 5.2.3 CNS cell type specific EV markers

Putative protein markers of neurons (e.g., NCAM1, Sandau et al., 2022; Soares Martins et al., 2018), astrocytes (e.g., GLAST) (Cressatti et al., 2021; Sandau et al., 2022)), microglia (e.g., CD11b (Guo et al., 2020; Herman et al., 2023; Pieragostino et al., 2019; Sandau et al., 2022; Verderio et al., 2012)) and oligodendrocytes (e.g., MOG) (Galazka et al., 2018)) have been used to identify CNS cell‐type specific EVs in pooled or individual CSF samples (see Table 5 for acronyms, and Table S4 for full protein names). Methodologies used to identify these proteins include immunoblot, ELISA, FC, immunoEM and unbiased MS. Currently, there is no definitive marker for neuron‐specific EVs, although a number of reports have used L1CAM for affinity purification of neuron‐derived EVs from human biofluids. L1CAM is a cell adhesion molecule involved in several functions such as neuron‐neuron adhesion and neurite outgrowth (Moos et al., 1988). However, L1CAM is highly expressed in kidney, urinary bladder and skin (https://www.proteinatlas.org/ENSG00000198910‐L1CAM/tissue), and it is overexpressed in many human cancers in organs outside of the CNS (Ganesh et al., 2020; Giordano & Cavallaro, 2020). L1CAM immunoaffinity‐capture has been used to separate ‘CNS‐EVs’ from AD patient plasma, which showed a significant increase in Aβ_1−42_ up to 10 years before the onset of AD (Fiandaca et al., 2015). Subsequently, a number of studies, predominately with blood and to a lesser extent CSF, have used L1CAM pull‐down strategies (Anastasi et al., 2021; Banack et al., 2020; Cha et al., 2019; Cicognola et al., 2019; Delgado‐Peraza et al., 2021; Fu et al., 2020; Goetzl et al., 2016; Guix et al., 2018; Jiang et al., 2021, 2020; Niu et al., 2020; Shi et al., 2014; Zhao et al., 2020). Concerns regarding L1CAM to separate for ‘neuronally derived’ EVs stem from inconsistent findings of L1CAM in human CSF EVs (Muraoka, Jedrychowski, et al., 2020; Muraoka, Lin, et al., 2020; Muraoka et al., 2019). Furthermore, separation and concentration of CSF EVs using a combination of UF + SEC revealed that the majority of L1CAM co‐eluted with soluble CSF albumin and was only faintly detected in early‐eluting EV fractions (Costa et al., 2020). More recently, use of ultrasensitive ELISAs with EV fractions from CSF or plasma separated by either SEC or density gradient dUC revealed that L1CAM distribution was distinct from tetraspanin EV markers, consistent with a soluble protein rather than an EV‐associated protein (Norman et al., 2021). In an effort to further assess if L1CAM is associated with neuron‐derived EVs, a proteomics study detected L1CAM in the cell lysates of human iPSC neurons, but did not detect L1CAM in EVs derived from these cells (You et al., 2022). Further studies are needed to determine the tissues‐ and cell‐ types that express L1CAM on EVs in normal and diseased states. For astrocyte‐derived EVs, GLAST is the most well documented EV marker. Of microglia‐derived EV markers, CD11b, CD45 and HLA‐DR are commonly found on myeloid cells thus not specific to microglia; CD86 is a ligand of CD28 expressed on B‐cells (Greenwald et al., 2005); and CD105 is mainly found in vascular endothelium (Duff et al., 2003). Oligodendrocyte‐derived EV markers include GALC (Galazka et al., 2018; Masvekar et al., 2019) and MOG, a major component of myelin sheets specific to mature oligodendrocytes (Peschl et al., 2017). Transthyretin has been used as a marker for choroid plexus‐derived EVs (Balusu et al., 2016; Castañeyra‐Ruiz et al., 2022; Vandendriessche et al., 2021).

New markers for CNS cell‐type specific EVs have also been proposed (see Table 5 for acronyms, and Table S4 for full protein names). A recent study used differentiated iPSCs to identify neuronal, astrocyte, microglia and oligodendrocyte EV markers (You et al., 2022). ATP1A3 was identified as a neuronal marker, ITGA6 and LRP1 were identified as astrocyte markers, and LCP1 was identified as a microglial marker. Oligodendrocyte markers included LAMP2 and FTH1. All of these markers (ATP1A3, ITGA6, LRP1, LCP1, LAMP2 and FTH1) were also shown to be expressed in human brain‐derived EVs. Additionally, the myelin‐associated proteins PLP1 and MBP were detected in human brain‐derived EVs, but not in oligodendrocyte iPSCs EVs, likely due to the lack of myelin in the cultured cells (You et al., 2022). An independent study also demonstrated that human brain CD11b‐positive microglial EVs co‐express TMEM119, P2RY12, FTH1 and TREM2 (Cohn et al., 2021). With the exception of TREM2, each of these neuronal‐, astrocyte‐, microglia‐ and oligodendrocyte‐EV markers are detected in human postmortem CSF EVs (Muraoka, Jedrychowski, et al., 2020). Additional studies are needed to assess these cell type‐specific protein markers in CSF from healthy and neurologically‐impaired living human donors, as disease states can alter the expression of some of these markers (Cohn et al., 2021; Muraoka, Jedrychowski, et al., 2020; You et al., 2022).

### 5.3 Recommendations and reporting guidelines for CSF EV protein studies

It is essential to consider the sensitivity and specificity of each protein antibody, and to determine whether the protein marker is specific to the CSF, or to brain versus peripheral tissues when designing experiments, interpreting data and stating study limitations. In Text Box 4, and Table 1, the Task Force provides recommendations and the minimum reporting guidelines for CSF EV protein studies.

**Text Box 4. Recommendations and Reporting Guidelines for CSF EV Protein Studies**

**Recommendations**

- As CSF EVs are relatively low in abundance, assays with increased sensitivity such as ultrasensitive ELISAs are recommended for protein analysis when possible.
- Use well‐validated antibodies:
  i) For commercially sourced antibodies, use vendors that provide exemplary validation data with respect to specificity
  ii) For custom antibodies, validate specificity (e.g., using cell culture lines known to express (positive for) and not to express (negative for) the target antigen)
- To ascribe a biological function to immunoisolated CSF EVs presumed to originate from a single cell type (e.g., neurons) demonstrate both the tissue‐ and cell type‐specificity of the protein markers using positive and negative tissue‐ and cell‐type controls.
- Considering that few, if any, protein markers are tissue‐ and/or cell‐type specific for neuron, astrocyte, microglia, and oligodendrocyte EVs, enhanced specificity may be achieved through a combination of markers.

**Reporting Guidelines**

- Lower limits of detection for assays must be reported and results interpreted accordingly.
- For primary and secondary antibodies report:
  i) Source/vendor
  ii) Clone
  iii) Dilutions
  iv) Immunolabelling methods (e.g., blocking solution and procedure, antibody diluent, incubation times, incubation temperature, washes)
- Report the specificity and reproducibility for antibodies used for immunoisolation and immunolabelling.
- Until immunoisolation assays are validated as both tissue and cell type specific, refer to a pool of EVs as immunopositive for a marker as opposed to cell‐type derived (i.e., L1CAM+ EVs fractionated from CSF instead of neuronal‐derived EVs).

## 6 CSF EV RNA STUDIES

### 6.1 Current reporting of CSF EV RNA studies

Respondents to the CSF Task Force survey indicated the use of qPCR (17 respondents, 36%), long and small RNA‐seq (11 respondents, 23%), spectrophotometry (e.g., NanoDrop; 10 respondents, 22%), electrophoresis (e.g., Bioanalyzer; 7 respondents, 15%) and direct digital detection (e.g., NanoString; 2 respondents, 4%) as methods to assess CSF EV RNA. The literature review showed that 64 CSF EV articles analysed RNA (38%). Of these, qPCR (53 articles, 83%) was the most prevalent method for quantifying RNA, followed by RNA‐seq (23 articles, 36%), and direct digital detection (e.g., NanoString)/hybridisation arrays (e.g., Affimetrix) (6 articles, 9%). See Tables S5–S7 for individual references on each RNA quantification method, discussed above. Orthogonal techniques to validate RNA‐seq and direct detection/hybridisation data can be used to promote reproducibility of results. For the 64 CSF EV studies that analysed RNA, eight articles (13%) used both RNA‐seq and qPCR (Chen et al., 2022; Hou et al., 2019; Lee et al., 2021; Otake et al., 2019; Prieto‐Fernández et al., 2019; Saugstad et al., 2017; Torii et al., 2022; Yagi et al., 2017), four (6%) used both direct digital detection/hybridisation arrays and qPCR (Feliciano et al., 2014; He et al., 2019; Kim et al., 2020; Wang et al., 2020), and one (2%) used both RNA‐seq and direct digital detection/hybridisation arrays (Tietje et al., 2014).

### 6.2 Current best practices for CSF EV nucleic acid studies

Early CSF EV miRNA studies predominantly reported on gliomas (Akers et al., 2015, 2013; Chen et al., 2013; Figuero et al., 2017; Shi et al., 2015), and now include studies that profiled small (miRNA, piwi‐interacting RNA) and long (mRNA and lncRNA) RNAs in many neurological diseases including meningeal carcinomatosis (Cheng et al., 2021), encephalitis (Li et al., 2022), post‐haemorrhagic hydrocephalus (Spaull et al., 2019) and neurodegenerative diseases such as amyotrophic lateral sclerosis (Otake et al., 2019), AD (Jain et al., 2019), Huntington's disease (Sanchez et al., 2021) and spinocerebellar ataxia type 3 (Hou et al., 2019). While most studies have profiled miRNA or mRNA (Akers et al., 2017; Chen et al., 2013; Cheng et al., 2021; Egyed et al., 2020; Figuero et al., 2017; Hou et al., 2019; Li et al., 2022; Otake et al., 2019; Sanchez et al., 2021; Saugstad et al., 2017; Shi et al., 2015; Spaull et al., 2019; Tietje et al., 2014), one study reported that piwi‐interacting RNAs are abundant in CSF EVs and could be candidate disease biomarkers (Jain et al., 2019). The quantity and identity of miRNAs detectable in CSF EVs varies greatly, likely reflecting biological or disease‐related changes in expression, and/or technical differences in CSF collection and processing or RNA isolation, processing, or profiling methods. High‐abundance miRNAs in CSF EVs across different datasets in health and neurologic disease include miRs‐21, ‐22, ‐148a and ‐181 (Alsop et al., 2022; Jain et al., 2019; Lee et al., 2021; Prieto‐Fernández et al., 2019; Qi et al., 2022; Tietje et al., 2014; Torii et al., 2022). Further, miR‐1298 is brain‐enriched (Alsop et al., 2022) and detected in CSF, relative to 12 other biofluids (Alsop et al., 2022; Godoy et al., 2018; Yagi et al., 2017). In healthy controls, EVs from 1 mL of CSF have been reported to contain up to ∼85 miRNAs (Saugstad et al., 2017), while mRNAs in the same CSF EVs contain ∼2800 mRNAs (Saugstad et al., 2017).

A major challenge for RNA‐seq and direct digital detection/hybridisation arrays is the low RNA yields from CSF EV preparations. More recently, the development of specialised kits that are specific to biofluid based specimens are becoming popular. Kits such as Norgen Urine miRNA Purification Kit has successfully been used with CSF (Kopkova, Sana, Fadrus, Machackova, et al., 2018; Kopkova, Sana, Fadrus, & Slaby, 2018) and thus may be applicable to CSF EVs. Considering the low levels of RNA in CSF EV preparations, electrophoresis chip assays, such as the Agilent Bioanalyzer RNA 6000 Pico and RNA 6000 Nano chip assays, are recommended for assessing RNA integrity and RNA concentration values as these are more sensitive than UV‐Vis spectrometry alone. However, there are limitations to electrophoresis chip assays. The Pico Chips have the greatest sensitivity yet are considered qualitative with a 50 pg/μL total RNA limit of detection (Agilent; (Agilent, 2020)) which may still be insufficient for CSF EV RNA measurements. Also, according to Agilent, chip data may be affected by salt and ion concentrations, as a result it is recommended to use RNA eluted with de‐ionised water for best assay performance. Below we provide details for the current best practices for CSF EV (i) quantitative PCR; (ii) RNA‐Seq and (iii) hybridisation assays.

#### 6.2.1 CSF EV quantitative PCR

A summary of CSF EV RNA qPCR studies to date is presented in Table S5. The first study on CSF EV mRNAs was published in 2013 (Chen et al., 2013). Of the >50 studies since then, 47 (89%) used human CSF, 4 (8%) used mouse CSF, 4 (8%) used rat CSF, and 1 (2%) used sheep CSF. Of these, 33 (62%) reported the CSF starting volume, which ranged from 200 μL to 15 mL. In one study, ∼100 μL aliquots of individual CSF samples were used to generate a pool of CSF. CSF EV separation/concentration was most commonly performed using commercially available resources such as membrane affinity and polymer precipitation‐based kits (35 articles, 6%). Other separation and concentration methods include UC or dUC (14 articles, 26%), a combination of dUC plus SEC or UF (2 articles, 4%), sucrose density dUC (2 articles, 4%), SEC (1 article, 2%) and sequential UF (1 article, 2%). RNA isolation and quantification methods were standard approaches. Most qPCR studies to date measured CSF EV RNAs with commercially available primers plus fluorescence double stranded DNA dyes (32 articles, 60%) or primers plus fluorescent conjugated probes (26 articles, 49%). Data were normalised using standard methods specific to each assay/experiment (global normalisation, endogenous non‐changing miRNAs or exogenous spike‐ins) to obtain relative or absolute quantification. Unfortunately, there are no specific non‐changing CSF miRNAs that we can recommend at this time for use in all CSF studies, as they are likely dependent on study participants or disease state. For example, there are endogenous non‐changing miRNAs specific to CSF in older adults with AD versus cognitively normal participants (Wiedrick et al., 2019). But these miRNAs may not be recommended for studies focused on younger participants, or those with different CNS diseases. To identify miRNAs that are highly abundant in control CSF, we compared the expression profiles from CSF EV miRNAs assayed by qPCR (Sandau et al., 2022) and by RNA‐seq (Alsop et al., 2022) and found miR‐204‐5p, 30c‐5p and 150‐5p in the top 25 for both datasets. Thus, these miRNAs may serve as positive controls for CSF miRNA studies regardless of assay parameters.

As dUC is a labour‐intensive method for isolating CSF EVs, one study compared dUC to an expedited dUC method and to a commercially available membrane‐based affinity capture method in CSF collected from glioblastoma patients (Akers et al., 2015). Similar amounts of miRNAs were recovered by dUC and affinity capture and with CSF the 120,000 × *g* pellet was enriched for miRNAs relative to the CSF 10,000 × *g* pellet (Akers et al., 2015). This was the first study to show that a viable, alternative expedited method can be used for EV isolation, and that CSF miRNAs are enriched in 120,000 × *g* pellet (Akers et al., 2015). A subsequent multi‐site study examined the EV and RNA composition of pooled CSF samples from controls, AD, PD, low‐grade glioma, glioblastoma multiform and subarachnoid haemorrhage, representing neurodegenerative disease, cancer and severe acute brain injury (Saugstad et al., 2017). Pooled CSF was shared between three sites, and all experiments done using identical reagent lots and protocols. Based on the outcomes, recommendations were made for collaborative, multi‐site studies when evaluating CSF samples. These include using stringent standard operating procedures for the collection, processing and storage of CSF samples, standardised RNA isolation kits and methods, use of an RNA reference when different instruments are used to measure RNA, use of identical analytic platforms for consistency in expression results, and rigorous data collection and management policies (Saugstad et al., 2017).

#### 6.2.2 CSF EV RNA sequencing

To date, most total CSF or CSF EV RNA studies have been performed using long or small RNA‐seq and reported changes in CSF EV miRNA levels in neurodegenerative disorders (AD, PD), brain tumours (gliomas, meningiomas) or brain injury (stroke), as well as in the levels of piwi‐interacting RNAs and Y RNAs (Table S6). Although many of the CSF EV RNAs are mRNA (Saugstad et al., 2017), to date only 5 of the 23 (22%) CSF EV RNA‐seq publications performed long RNA‐seq (Table S6). Despite the low number of long RNA‐seq publications, long RNA‐seq kits are more adaptable to low input RNA samples, and several manufacturers provide kits for RNA inputs in the picogram range. To improve sequencing data from CSF EVs with a low input of RNA, studies with access to larger volumes of CSF collected in the same manner as the study samples should test one or two sequencing kits with varying PCR cycles. Further considerations should be given to the EV separation/concentration method and RNA isolation kits used. In line with this, one study assessed vesicular (EV‐enriched) and non‐vesicular (EV‐depleted) miRNAs from CSF obtained from paediatric patients aged 0–7 years using five protocols (Prieto‐Fernández et al., 2019). Of these protocols, three used an EV separation/concentration step, and two directly isolated miRNAs from total CSF. The efficiency of each protocol was assessed by qPCR and small RNA‐seq to profile miRNAs. Starting with 200 μL of CSF, a total of 281 miRNAs were identified across all five protocols, and a commercially available PEG precipitation kit recovered the largest number of miRNAs but this method is not EV‐specific. In SEC fractions that contained CSF EVs, 79 miRNAs were detected, 12 of which were not detected in the EV‐depleted fractions (Prieto‐Fernández et al., 2019).

#### 6.2.3 CSF EV hybridisation assays

RNA hybridisation methods offer benefits compared to qPCR and RNA‐seq, such as the ability to probe for specific targets in a single experiment, a short experimental time and costs that are typically less than for RNA‐seq. One limitation is the requirement for a higher RNA input (>100 ng total RNA) relative to qPCR and low input RNA‐seq library preparation kits, with RNA input requirements in the picogram to low nanogram range. Thus, input requirements for hybridisation assays may preclude assessing CSF EV RNAs from individual donors depending on the volume available and/or yield of EV RNA. Of the six studies in the literature review, only one (16%) reported the CSF starting volume (7 mL), which impedes reproducibility and limits our understanding of the actual potential of using hybridisation assays for CSF EV RNA (for references see Table S7).

In the literature review 1 article used direct digital detection (NanoString) and 5 used hybridisation microarrays (Agilent, Arraystar, Affymetrix) for profiling CSF EVs. Of these six studies, two (33%) used dUC ± sucrose density gradient, one (16%) combined SEC and precipitation and the remaining three (50%) separated/concentrated EVs by precipitation methods (for references see Table S7). Of the survey respondents, less than 8% recommended the use of PEG or other precipitation‐based methods for EV separation/concentration, while ∼21% recommended UC and ∼26% recommended SEC. In the literature review, standard kits were used to isolate CSF EV RNA for hybridisation assays and one study enriched for circular RNA by digesting linear RNA with RNase R. After acquisition of the data, RNA normalisation was integrated into the analysis using vendor software packages or outsourced for processing, normalisation, and data analysis according to the vendor's recommendations (for references see Table S7).

While these studies demonstrate the feasibility of using hybridisation assays for human CSF EVs, the differences in EV separation/concentration, RNA isolation kits, hybridisation assays and data analysis present a challenge in comparing the results between studies. To improve hybridisation data for CSF EVs, studies with pools of larger volumes of CSF are needed to compare the different hybridisation platforms, with considerations given to the EV separation method and RNA isolation kits used. To promote reproducibility of study findings, we recommend orthogonal validation of hybridisation data. In line with this, five (83%) of the six published CSF EV RNA hybridisation studies validated the miRNA or circular RNA results by qPCR using custom oligos or miRNA primer‐probe qPCR assays, while the sixth study performed small RNA‐seq in conjunction with the miRNA microarrays (for references see Table S7).

### 6.3 Recommendations and reporting guidelines for CSF EV RNA studies

In Tables S1 and S4–S6, many of the metrics that would be useful for making recommendations for RNA analysis of CSF EV samples are missing, making it difficult to assess the quality and quantity of CSF samples typically obtained. In Text Box 5, and Table 1, the Task Force provides recommendations and minimum reporting guidelines regarding for CSF EV RNA studies.

**Text Box 5. Recommendations and Reporting Guidelines for CSF EV RNA Studies**

**Recommendations**

- If there is sufficient CSF EV RNA, use a low‐input fluorescent RNA abundance assay in triplicate to accurately quantify RNA (more sensitive than UV‐Vis spectrophotometry).
- If there is not enough RNA to quantify, load equal volumes of RNA into each PCR reaction.
- Use a spike‐in and endogenous non‐changing miRNA(s) for calibration and normalisation of the data, respectively.
- If there is sufficient CSF volume for EV RNA analysis and CSF quality control, test CSF for blood contamination, which significantly affects RNA profiles.
- To promote reproducibility, use an orthogonal method to validate data from RNA‐seq and hybridisation assays.
- To improve CSF EV RNA data, more studies with pooled CSF are needed to assess:
  - RNA sequencing kits with varying PCR cycles
  - Hybridisation array platforms
  - Impact of EV separation/concentration method and RNA isolation kits

**Reporting Guidelines**

- A majority of the CSF Task Force survey respondents indicated the following must be reported:
  - RNA isolation kits used and modifications to kit protocols
  - Input volume or concentration of CSF EVs RNA
  - Data normalisation method(s)
- The CSF Task Force recommends that, in addition to the recommendations of the survey participants, the following must be reported:
  - Reagents and kits, including vendor and catalogue numbers, used for library preparation, sequencing, qPCR and hybridisation
  - Methods used to quantify RNA and perform quality control
  - Primer and probe sequences
  - Detail the workflow/methods and any modifications to kit protocols:
    - Number of PCR cycles
    - Changes in ligation parameters from manufacture's recommendations
    - Use of post‐PCR purification and size selection of RNA‐seq libraries
- For CSF EV RNA‐seq data the following must be reported:
  - Mapping percentages to the human genome/transcriptome (or species being studied)
  - Number of genes (protein‐coding and long non‐coding RNAs)

## 7 CSF EV GLYCOMIC AND GLYCOPROTEOMIC STUDIES

### 7.1 Current reporting of CSF EV glycoproteomic studies

Respondents to the CSF Task Force survey indicated glycoproteomics as an area of interest for EV biomarker studies. EVs may include glycoconjugates such as glycoproteins, proteoglycans and glycolipids, in which glycans are covalently bound to proteins or lipids (Costa, 2017; Williams et al., 2018). Glycans on the surface of EVs may play important functional roles, including cell recognition and internalisation processes. For example, sialic acid and N‐glycans tune EV uptake (Williams et al., 2019). Despite the importance of glycans, systematic characterisation of the glycan repertoire (glycomics) or of glycans together with their glycosites within the protein (glycoproteomics), has been scarcely approached in CSF EVs. However, it constitutes an interesting topic of investigation since changes in glycosylation are observed in CNS diseases (Conroy et al., 2021), including neurodegeneration (Gaunitz et al., 2021; Moll et al., 2020), brain cancer (Dusoswa et al., 2020; Veillon et al., 2018) or most congenital disorders of glycosylation (Freeze et al., 2015). Structures and functions of glycans on EVs have been reviewed elsewhere (Costa, 2017; Gerlach & Griffin, 2016; Grzesik et al., 2023; Harada et al., 2021; Macedo‐da‐Silva et al., 2021; Martins et al., 2021; Williams et al., 2018). Thus, here we address methods used in glycoproteomic analyses of EVs from different sources, and have compiled recent studies in Table S8 whose strategies are applicable to CSF EVs.

### 7.2 Current best practices for CSF EV glycomic and glycoproteomic studies

Glycan characterisation techniques should be selected based on the type of glycoconjugate and structures investigated, the amount of material available, and the level of structural detail desired. To capture the structural complexity and diversity of glycans, complementary techniques are often needed. In glycomic studies, glycans are released from glycoproteins (e.g., with peptide N‐glycosidase F for N‐glycans), usually derivatised and in general analysed by LC and MS methods for structural elucidation being accomplished by exoglycosidase sequencing and/or MS/MS analysis; while glycoproteomics is based on LC‐MS/MS methodologies (Chau et al., 2023; Chen et al., 2021; Kobeissy et al., 2022; Lageveen‐Kammeijer et al., 2022; Riley et al., 2021; Williams et al., 2018). Lectin‐based arrays (Saito et al., 2018; Williams et al., 2019; Yokose et al., 2020), lectin blotting and immunoblotting (Escrevente et al., 2011; Freitas et al., 2019; Gomes et al., 2015; Zhang et al., 2018) are also useful for characterising the EV glycome. Lectins specifically recognise glycan motifs and require only a few micrograms of glycoconjugate input, less than is needed for structural analysis by LC and/or MS. For example, human CSF N‐glycan structures have been characterised in detail from ∼20 to 30 μg protein (Gonçalves et al., 2015; Stanta et al., 2010). However, a recent advancement with a multi‐glycomics strategy yielded structural information on several glycan types from 20 μg protein in a single CSF sample (Moh et al., 2022).

### 7.3 Recommendations for CSF EV glycomic and glycoproteomic studies

Glycome and glycoproteomic analysis of EVs from various biological sources have been reported in the literature (Table S8), and can be applied to CSF EVs. In this context, the following recommendations for CSV EV glycomic and glycoproteomic analysis are provided (Text Box 6).

**Text Box 6. Recommendations for CSF EV Glycomic and Glycoproteomic Studies**

- If the amount of CSF available is low, use EVs from CSF pools for global glycosylation characterisation.
- For detailed glycan structure analysis, protocols involving HPLC and MS are required for use with either CSF pools or individual samples.
- To search for biomarkers, different physiological conditions (e.g., disease and control subjects) may be characterised in pools. If targets are identified, screening assays in individual CSF samples should be performed.
- High‐throughput glycomics methods are recommended for biomarker studies involving large populations due to their sensitivity and ability to yield detailed structural information, the parallel processing of a high number of samples and potential for automation (Trbojević‐Akmačić et al., 2022).
- Lectins and carbohydrate‐specific antibodies are valuable and sensitive tools used in arrays, blotting or other detection techniques (e.g., microscopy, FC) and are adequate for EV glycoconjugate characterisation.
- Use competitive sugars or glycosidase negative controls to confirm specificity of carbohydrate‐specific antibody binding (Batista et al., 2011; Escrevente et al., 2011; Gomes et al., 2015).
- A key issue is the availability of glycan standards with well‐defined structures to represent the immense repertoire of existing glycans and the high structural complexity (Costa et al., 2018, 2019; Harada et al., 2019; Sastre Toraño et al., 2021; Zhang et al., 2018). Standards are required for verification of:
  - Analytical procedures
  - Comparability among different studies
  - Quantitation purposes
- Standards support the development of high‐resolution analytical techniques (e.g., ion mobility MS) (Sastre Toraño et al., 2021).
- In addition to detecting glycan structures, site identification and occupancy within the protein may be of interest (Horowitz et al., 1992), in which case a glycoproteomics approach is appropriate (e.g., (Flowers et al., 2020)).
- Use specific databases and software tools for glycoproteomics data analysis and interpretation (Aoki‐Kinoshita et al., 2022).

## 8 CSF EV LIPIDOMIC STUDIES

### 8.1 Current reporting of CSF EV lipidomic studies

Respondents to the CSF Task Force survey indicated the use of lipidomics as an area of interest for EV biomarker studies. Many neurological disorders are characterised by altered CNS lipid profiles, including multiple sclerosis, amyotrophic lateral sclerosis, neurodegenerative and neuropsychiatric diseases, epilepsy, stroke, traumatic brain injury and HIV‐associated neurocognitive disorder (Adibhatla & Hatcher, 2007; Haughey et al., 2013). A limited number of lipidomic studies have been conducted with human brain tissue EVs or CSF EVs (Cohn et al., 2021; Kurzawa‐Akanbi et al., 2021; Su et al., 2021). A summary of the relevant lipidomic studies to date is presented in Table S9.

### 8.2 Current best practices for CSF EV lipidomic studies

Three studies have been published on brain‐derived EVs using different lipid extraction methods, two for AD and the third for Lewy body dementia (Cohn et al., 2021; Kurzawa‐Akanbi et al., 2021; Su et al., 2021). One study used a monophasic lipid extraction and semi‐quantitative ultrahigh resolution and accurate mass spectrometry (UHRAMS) for lipidome analysis of EVs from the frontal cortex to demonstrate that brain‐derived EVs are (i) enriched with phosphatidylserine lipids, and (ii) in AD there are alterations in glycerophospholipid and sphingolipid levels, compared to control EVs (Su et al., 2021). Another study used Bligh‐Dyer lipid extraction and LC‐MS to analyse microglia‐derived EVs from parietal cortex and showed that EVs in AD had a proinflammatory profile based on increases in bis(monoacylglycerol)phosphate and monohexosylceramides, as well as decreases in phosphatidylethanolamine, phosphatidic acid and phosphatidylserine lipids (Cohn et al., 2021). Lastly, lipids extracted by methanol/chloroform from both brain‐derived EVs and postmortem CSF EVs showed ceramide enrichment in Lewy body dementia patients, compared to control (Kurzawa‐Akanbi et al., 2021). To date, this is the only study to have profiled lipids in CSF EVs. These studies implicate the use of CSF EV lipid profiles as potential biomarkers for neurological disorders. As with other analytic measures, one challenge with CSF lipidomics is the low yield of EVs in CSF relative to plasma/serum (Brennan et al., 2020; Chen et al., 2013; Muraoka, Lin, et al., 2020). Considering that EV separation methods and lipid extraction methods (Reichl et al., 2020) have differences in yield, systematic studies to determine the CSF volume requirements and the impact of different lipid extraction methods are needed.

### 8.3 Recommendations for CSF EV lipidomic studies

A recent publication used total CSF to test four different lipid extraction methods (i) Folch (Folch et al., 1957), Bligh & Dyer (Bligh & Dyer, 1959); (ii) MTBE (Folch et al., 1957); (iii) BUME (Löfgren et al., 2016) and (iv) MMC (Pellegrino et al., 2014) as well as processing temperatures and mixing times (Reichl et al., 2020). A modified Folch method, which uses methanol/chloroform (1/2, v/v), extracted a broad range of lipids, including glycerophospholipids, glycerolipids and sphingolipids. However, biphasic methods are more laborious and require absolute accuracy in pipetting when the colleting aqueous/organic phase. A monophasic lipid extraction (isopropanol/methanol/ chloroform (4:2:1, v:v:v)) recovers highly polar and non‐polar lipids, including low‐abundance highly‐polar lipids such as gangliosides (specifically enriched in brain/CNS and thus indicating CNS origin) and sphingolipids (signature EV lipids) (Lydic et al., 2014). Including cooling solvents and equipment has also been shown to improve lipid extraction efficiency (Reichl et al., 2020). Also, optimising the mixing time for the lipid classes of interest improves recovery, since excessive mixing can lead to lipid degradation (Reichl et al., 2020). Antioxidants such as butylated hydroxytoluene can be added to prevent oxidisation and degradation of lipids that contain polyunsaturated fatty acid moieties, oxidised lipids and eicosanoids (Burla et al., 2018; Yehye et al., 2015). Lipidomic analysis of EVs from brain and CSF (see Table S9) as well as total CSF can be applied to CSF EVs. In this context, the following recommendations for CSV EV lipidomic analysis are provided (Text Box 7).

**Text Box 7. Recommendations for CSF EV Lipidomic Studies**

- Reduce lipid droplets or lipoproteins present in the EV preparation as these could confound CSF EV lipid profiles (e.g., for SEC, select large pore sized resin and collect narrower fractions to remove lipoproteins and increase CSF EV purity).
- More studies are needed to assess the impact of lipid extraction methods on the lipid profile of CSF EVs.
- More studies are needed to optimise CSF EV lipid extraction protocols (e.g., temperature, mixing times, antioxidants).
- Improved accuracy in lipid quantification enables reproducibility and cross referencing between laboratories:
  - Report normalized data instead of the lipid peak intensity from raw MS spectra
  - For absolute quantification normalise endogenous lipids against known amounts of internal standards from each lipid class
  - Use internal standards to correct for lipid extraction efficiency and ionisation efficiency
  - Normalize against other biological measures (e.g., CSF volume, EV particle number).
- Data with known lipid internal standards can be presented as:
  - Molar concentrations (e.g., μM)
  - Mol/CSF volume
  - Mol/EV particle
- Data without known lipid internal standards can be presented as:
  - Normalised to the total lipid content
  - Per lipid category content
  - Per lipid class content (e.g., expressed as mol%)

## 9 CSF EV METABOLOMIC STUDIES

### 9.1 Current reporting of CSF EV metabolomic studies

Respondents to the CSF Task Force survey indicated the use of metabolomics as an area of interest for EV biomarker studies. While these studies are relevant to neurological disorders, to date no studies have been conducted with CSF EVs. Table S10 includes publications from total CSF and non‐CSF EV studies. Herein, we provide a summary of the current state of the literature and recommendations and reporting guidelines for CSF EV metabolomic studies.

### 9.2 Current best practices for CSF EV metabolomic studies

EV metabolomics is a nascent field of great potential. Of the published EV metabolome studies, a majority relate to cell culture medium‐derived EVs, while EVs separated from body fluids like urine and blood are less represented (Altadill et al., 2016; Du et al., 2021, 2022; Luo et al., 2018; Nielsen et al., 2021; Puhka et al., 2017; Zebrowska et al., 2019). CSF EV metabolites have not yet been reported. However, the methodologies used to analyse the metabolites of EVs from other biological sources could be adequate. Metabolomic profiling can be performed with analytical techniques including gas chromatography‐MS, high‐field nuclear magnetic resonance and LC‐MS. These methodologies offer different advantages: gas chromatography‐MS has excellent inter‐lab reproducibility; nuclear magnetic resonance is highly selective and LC‐MS is highly sensitive (Palomo et al., 2014). The development of ultra‐performance LC has made it possible to achieve higher resolutions, higher sensitivities and more rapid separations compared with conventional LC (Nováková et al., 2006). However, no single method can completely extract all metabolites or cover the entire metabolome. Thus, some forethought must be given to the types of molecules to analyse, beyond considerations in EV sample preparation (Williams et al., 2019). For analysis of CSF EV metabolomics data, use of a combination of metabolomics and specialised software devoted to visualising cellular pathways could identify the main mechanisms that trigger a specific biological process mediated by CSF EVs (Palomo et al., 2014).

### 9.3 Recommendations for CSF EV metabolomic studies

For a summary of relevant EV metabolomics studies see Table S10. Based on metabolomic profiling of EV from other fluids, Text Box 8 contains recommendations for the analysis of CSF EVs.

**Text Box 8. Recommendations for CSF EV Metabolomic Studies**

- Seminal CSF EV metabolomics studies should include comparisons of a combination of different extraction methodologies and chromatographic parameters.
- Consider the detection limit and adequate sample volumes (Gézsi et al., 2019).
- The physicochemical diversity of the CSF metabolome requires the use of multiple instrumental analytical methods and complementary data acquisition modes to maximise the metabolome coverage, facilitate the identification of metabolites and overcome bias from individual techniques (Yan et al., 2021).
- It is highly recommended to combine MS‐based metabolomics and nuclear magnetic resonance spectroscopy as complementary techniques (Wishart, 2008). MS‐based metabolomics identify low abundance metabolites and nuclear magnetic resonance identifies core metabolites in key metabolic pathways (Stringer et al., 2016; Wilkins & Trushina, 2018).
- CSF EV metabolome characterisation should include a comparison with EV‐depleted fractions, as done for culture media experiments (Palomo et al., 2014).

## 10 CSF EV FUNCTIONAL STUDIES

Recommendations for evaluating the functional activity of EVs are comprehensively outlined in the MISEV2018 guidelines (Théry et al., 2018). Where possible, functional assays for CSF EVs should follow these guidelines. Studies describing functional activities of CSF EVs are listed in Table S11. Limitations and challenges of functional studies of CSF EVs involve pre‐analytical variables (e.g., CSF collection, processing and storage) and EV separation methods. The collection site, collection time and method used to collect CSF may lead to considerably different levels of EVs in the CSF samples (Saugstad et al., 2017). Of the 24 functional activity CSF studies published thus far, all (100%) reported the methodological details of EV separation. The most common separation/concentration methods to assess the functional activity of CSF EVs were dUC (Herman et al., 2023; Madhankumar et al., 2017; Qiu et al., 2021; Ragonese et al., 2020; Stuendl et al., 2016; Wang et al., 2017) and UC (Bachy et al., 2008; Kong et al., 2018; Liu et al., 2014). Just over 50% of the publications specified details of CSF collection, such as the site and the collection volume (Table S11), which presents a challenge in replicating results in subsequent studies. Only two studies (8%) reported exclusion criteria based on blood contamination (Herman et al., 2023; Stuendl et al., 2016), which can greatly confound CSF EV functional studies.

## 11 CONCLUSIONS

**FIGURE 3:**
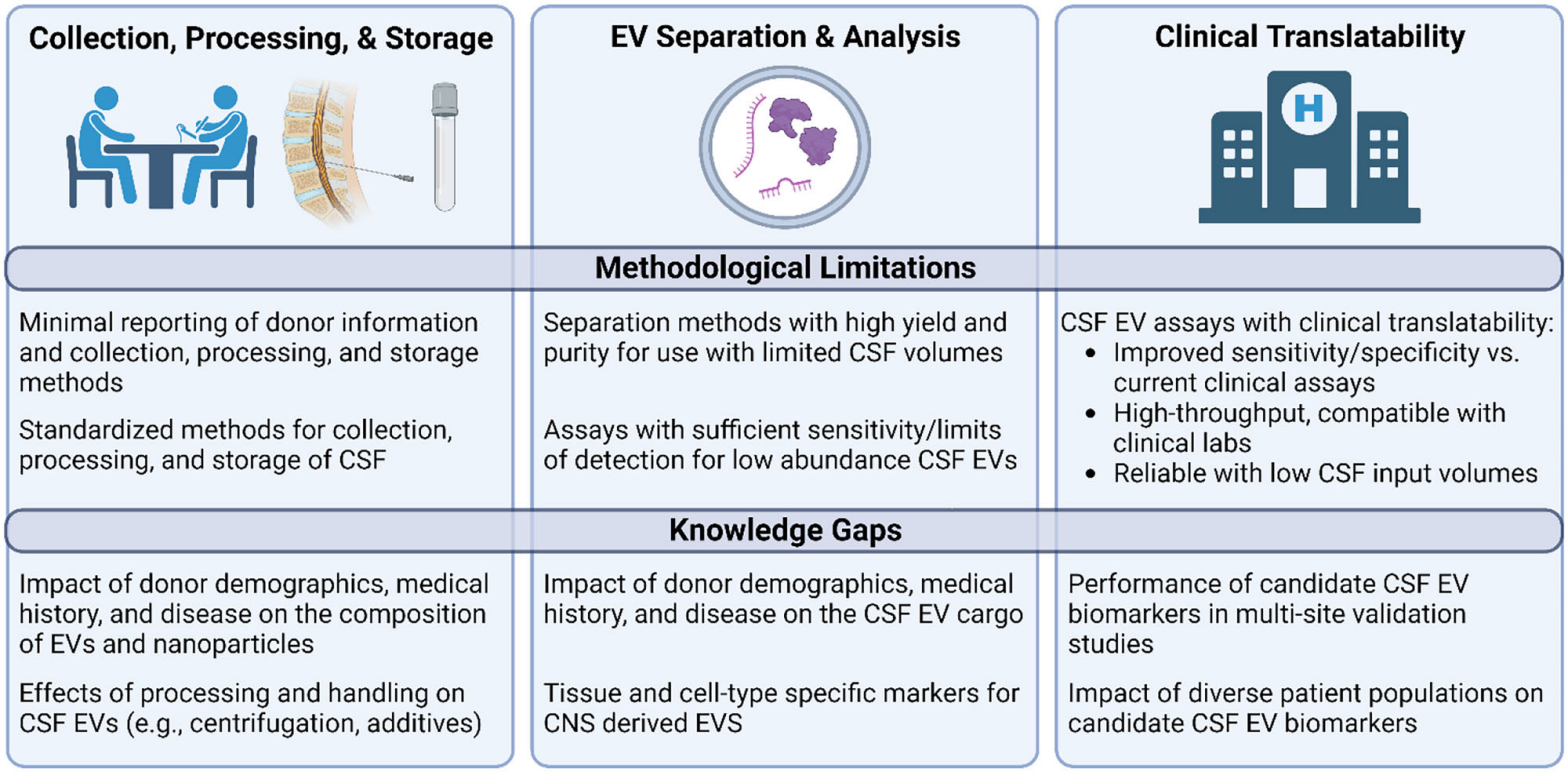
Methodological limitations and knowledge gaps for CSF EV studies. The CSF EV field is challenged by methodological limitations and knowledge gaps pertaining to (i) CSF collection, processing, and storage; (ii) EV separation and analysis and (iii) clinical translatability. Created with BioRender.com.

The main objective of the CSF Task Force is to provide recommendations and minimum reporting guidelines to increase and improve the reproducibility of CSF EV studies. A second objective is to identify the methodological limitations and knowledge gaps that are pertinent to CSF EV studies (Figure 3). For example, it is largely unknown how CSF collection, processing and storage effect EV composition and stability. Also, given the low abundance of EVs in CSF obtained by LP, there is a need to improve methods for EV separation/concentration that increase yield, without sacrificing purity. Likewise, there is a need to develop highly sensitive analytical methods to measure CSF EV size, number, and cargo, to discover and validate biomarkers for CSF EVs under normal and disease conditions, and to identify neuronal and glial and other brain protein markers on CSF EVs that are not expressed in peripheral tissues. This final point is especially important for the development of biomarkers that can be used to isolate brain/CSF EVs from peripheral biofluids such as blood and urine, to ensure that isolated EVs do not represent those from, for example, kidney or skin. In addition, as specificity is essential when attributing a biological function to an EV subtype, there is need for multiple marker assays and/or to identify and validate new proteins that are tissue‐ and cell‐type specific. And, relevant to the development of clinical biomarkers based on CSF EVs, there is a need to ensure that candidate biomarkers are validated in multiple cohorts across sites. For example, proteomic and transcriptomic profiles of EVs isolated directly from healthy and diseased brain tissue are currently being used to identify novel neuronal‐ and glial‐EV markers, and disease associated biomarkers (Huang et al., 2022; Perez‐Gonzalez et al., 2012; Su et al., 2021; You et al., 2022).

Of importance for re‐analysing data in the future, we encourage the use of EV‐TRACK (https://evtrack.org/) to reposit CSF EV data and to ‘facilitate standardization of EV research through increased systematic reporting on EV biology and methodology’. In addition, studies funded by the US National Institutes of Health are required to reposit raw data into Gene Expression Omnibus (GEO), a public functional genomics data repository supporting MIAME‐compliant data submissions (https://www.ncbi.nlm.nih.gov/geo/). GEO accepts array‐ and sequence‐based data, and provides tools to help users query and download experiments and curated gene expression profiles. Thus, investigators can use the metadata for any preferred analytic approach, including identifying candidate biomarkers based on studies with similar methods for CSF collection and EV separation/concentration. Such data would also be valuable to identify specific endogenous non‐changing CSF RNAs.

In conclusion, the CSF Task Force recommendations and guidelines for reporting are intended to enable researchers to compare and/or replicate results from individual studies. We have a unique opportunity and obligation as scientists and clinicians to positively impact CSF EV studies, particularly in the early days, of this exciting field of study, by reporting details that may ultimately give rise to new discoveries pertinent to the understanding of CNS functions, and to the development of novel therapeutics for CNS diseases.

## AUTHOR CONTRIBUTIONS

Ursula S. Sandau and Julie A. Saugstad enlisted interested members to the CSF Task Force team, created the survey with input from the Task Force, distributed the online survey, shared the results from survey with the Task Force team, managed meetings and communication with the team/authors, and incorporated all of the sections from authors into the manuscript. All authors provided intellectual contributions to the survey and manuscript, and reviewed and approved the final draft of the manuscript. Authors contributed to the manuscript as follows: Ursula S. Sandau: Conception, Abstract, Introduction, CSF Collection and Storage, EV Separation/Concentration (Lead), Size and Number, Protein, RNA, Lipidomics, Functional Assays, Conclusion, Figures, Tables, Supplemental Tables, Form page, Final Review and Editing; Setty M. Magaña: Abstract, Introduction, CSF Collection and Storage, EV Separation/Concentration, Protein, Figure, Tables, Supplemental Tables, Form page; Júlia Costa: Omics (Lead), Protein, Glycoproteomics, Tables, Supplemental Tables; John P. Nolan: Size and Number (Lead), Protein, Tables, Supplemental Tables; Tsuneya Ikezu: EV Separation/Concentration, Protein (Lead), Tables; Laura J. Vella: Introduction (Lead), Conclusion; Hannah K. Jackson: Functional Assays (Lead); Lissette Retana Moreira: Introduction, Metabolomics, Conclusion, Supplemental Tables; Paola Loreto Palacio: Introduction, Figure, Tables, Supplemental Tables; Andrew F. Hill: Introduction, Tables; Joseph F. Quinn: Introduction, CSF Collection and Storage; Kendall R. Van Keuren‐Jensen: RNA, Supplemental Table; Trevor J. McFarland: RNA, Supplemental Tables; Joanna Palade: RNA, Supplemental Table; Eric A. Sribnick: CSF Collection and Storage; Huaqi Su: Lipidomics; Kostas Vekrellis: Conclusion; Beth Coyle: Protein, Tables; You Yang: Figure 2b; Juan M. Falcón‐Perez: Conception, Advising, Glycomics; Rienk Nieuwland: Conception, Advising, Final Reviews, Editing; Julie A. Saugstad: Conception, Abstract, CSF Collection and Storage (Lead), RNA (Lead), Conclusion (Lead), Figures, Tables, Supplemental Tables, Form page, Final Review and Editing.

## CONFLICT OF INTEREST STATEMENT

LJV is on the Scientific Advisory Board of Exopharm Ltd (ASX:EX1); all other authors report no conflicts of interest.

## Supporting information

Supporting Information

Supporting Information

Supporting Information
