# Supplementary material for: Recommendations for reproducibility of cerebrospinal fluid extracellular vesicle studies
Source: J Extracell Vesicles. 2023 Dec 29;13(1):12397. doi: 10.1002/jev2.12397 (PMC10756860; doi:10.1002/jev2.12397)
Supplement: Supplementary file 1 — Supporting Information [file JEV2-13-12397-s003.pdf]

CSF EV Survey
May 6, 2021 12:53 PM MDT

Q1 - Q1. What is the source of your CSF? Please select all that apply.

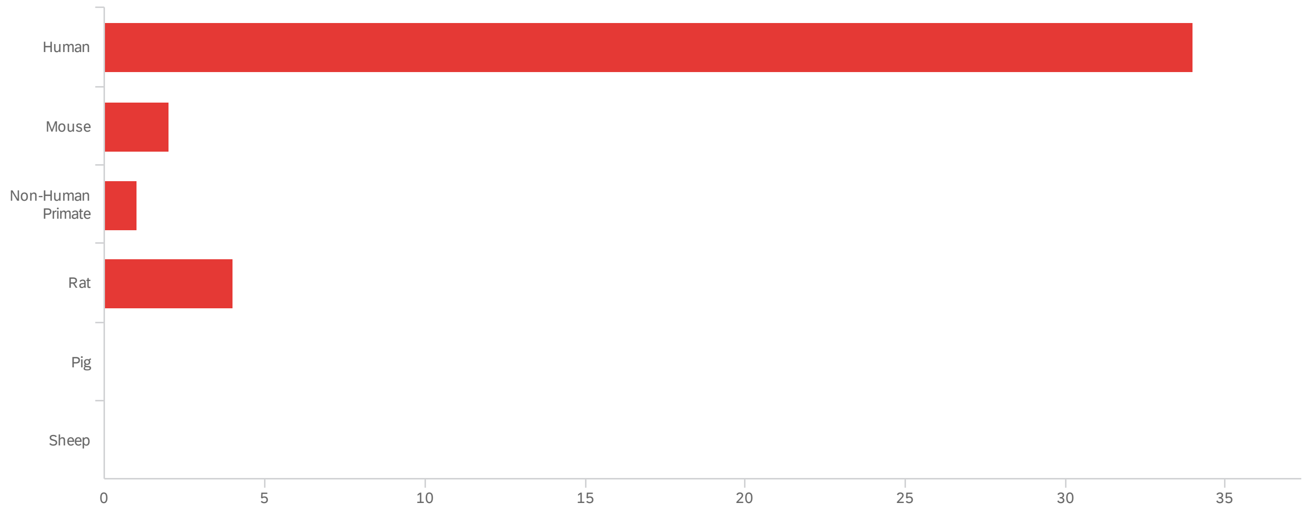

#	Field	Choice Count	
8	Human	82.93%	34
9	Mouse	4.88%	2
10	Non-Human Primate	2.44%	1
11	Rat	9.76%	4
12	Pig	0.00%	0
13	Sheep	0.00%	0
			41

Showing rows 1 - 7 of 7

Q1.1 - Q1.1 Other:

Q1.1 Other:

Q2#1 - Q2. What parameters might form reporting guidelines for CSF donor information?

Please rate the im... - Click to write Column 1

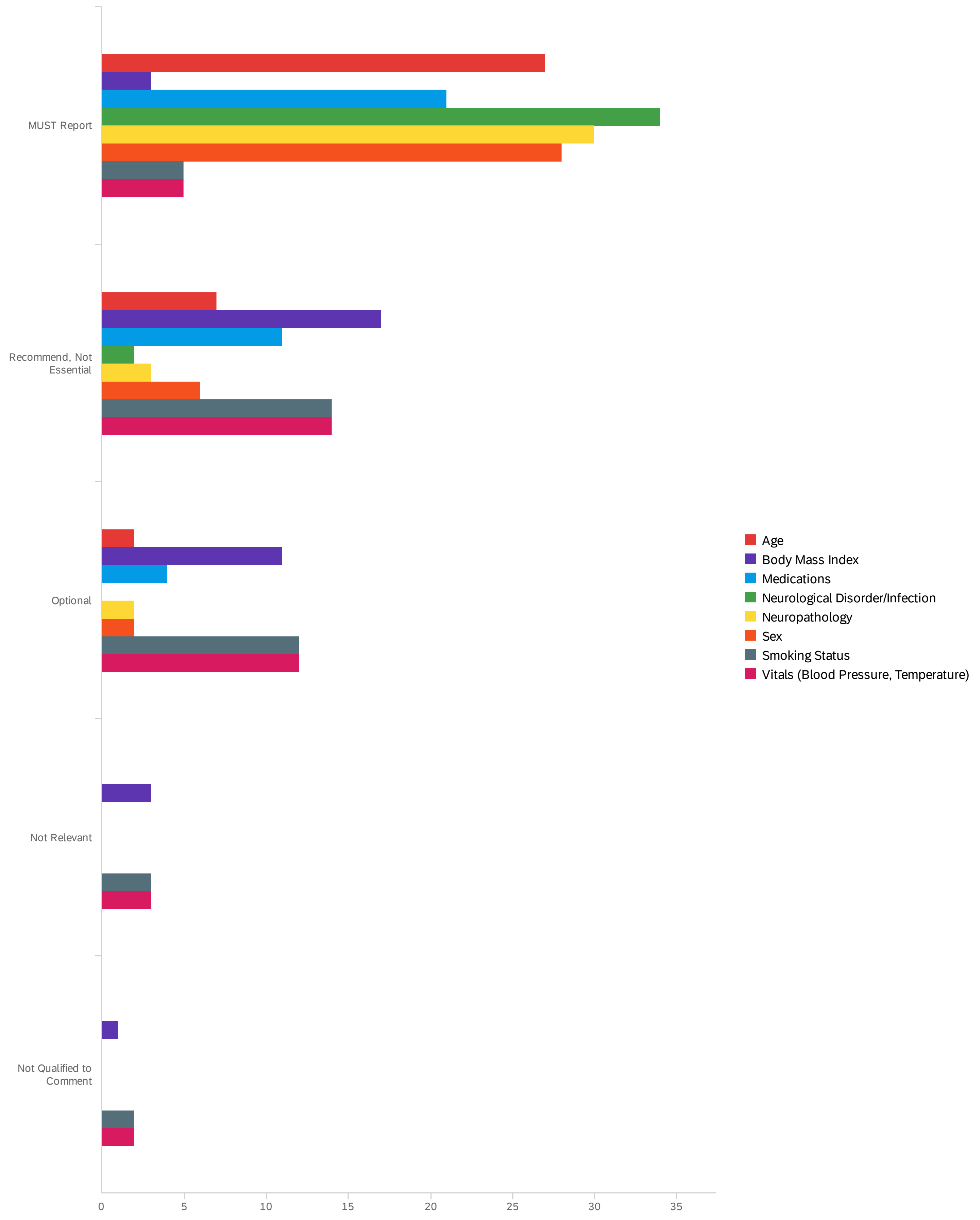

#	Field	Minimum	Maximum	Mean	Std Deviation	Variance	Count
1	Age	1.00	3.00	1.31	0.57	0.32	36

#	Field	Minimum	Maximum	Mean	Std Deviation	Variance	Count
2	Body Mass Index	1.00	5.00	2.49	0.87	0.76	35
3	Medications	1.00	3.00	1.53	0.69	0.47	36
4	Neurological Disorder/Infection	1.00	2.00	1.06	0.23	0.05	36
5	Neuropathology	1.00	3.00	1.20	0.52	0.27	35
6	Sex	1.00	3.00	1.28	0.56	0.31	36
7	Smoking Status	1.00	5.00	2.53	1.01	1.03	36
8	Vitals (Blood Pressure, Temperature)	1.00	5.00	2.53	1.01	1.03	36

#	Field	MUST Report		Recommend, Not Essential		Optional		Not Relevant		Not Qualified to Comment		Total
1	Age	75.00%	27	19.44%	7	5.56%	2	0.00%	0	0.00%	0	36
2	Body Mass Index	8.57%	3	48.57%	17	31.43%	11	8.57%	3	2.86%	1	35
3	Medications	58.33%	21	30.56%	11	11.11%	4	0.00%	0	0.00%	0	36
4	Neurological Disorder/Infection	94.44%	34	5.56%	2	0.00%	0	0.00%	0	0.00%	0	36
5	Neuropathology	85.71%	30	8.57%	3	5.71%	2	0.00%	0	0.00%	0	35
6	Sex	77.78%	28	16.67%	6	5.56%	2	0.00%	0	0.00%	0	36
7	Smoking Status	13.89%	5	38.89%	14	33.33%	12	8.33%	3	5.56%	2	36
8	Vitals (Blood Pressure, Temperature)	13.89%	5	38.89%	14	33.33%	12	8.33%	3	5.56%	2	36

Showing rows 1 - 8 of 8

Q2.1 - Q2.1 Other:

Q2.1 Other:
Unfortunately, rankings on your list require context. For many of them, ranking will depend on the targets and the pathological state of interest.
Cardiovascular disease, Inflammatory disorder

Q3 - Q3. What is the setting for your CSF collection? Please select all that apply.

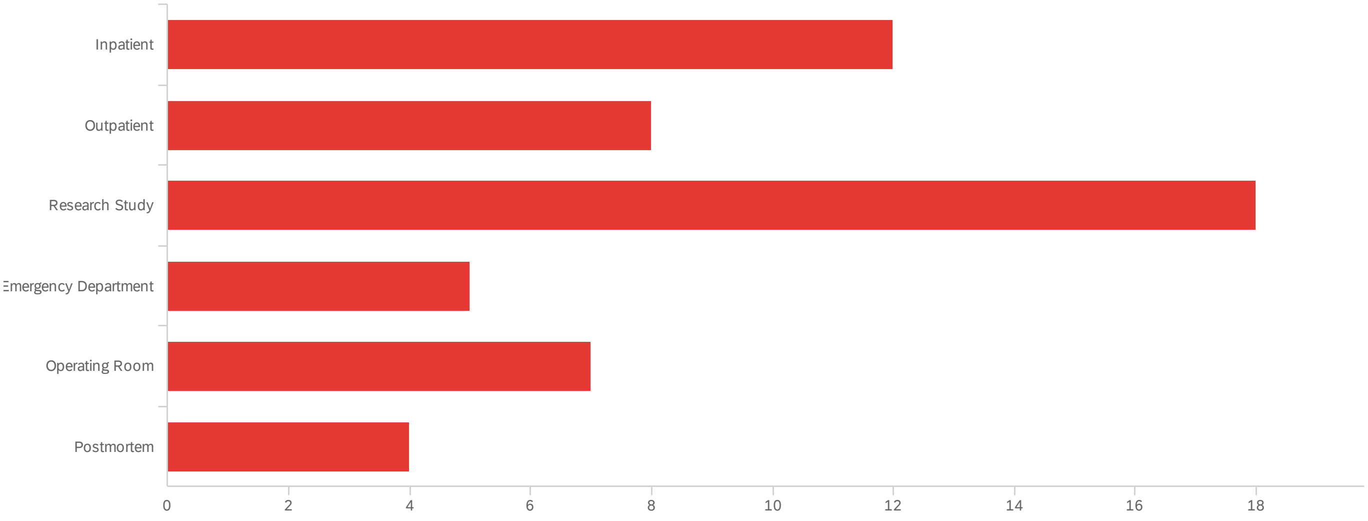

#	Field	Choice Count	
1	Inpatient	22.22%	12
2	Outpatient	14.81%	8
3	Research Study	33.33%	18
4	Emergency Department	9.26%	5
6	Operating Room	12.96%	7
7	Postmortem	7.41%	4
			54

Showing rows 1 - 7 of 7

Q3.1 - Q3.1 Other:

Q3.1 Other:
in vivo
Postmortem CSF is a strange fluid and should not be used in my opinion.

Q4 - Q4. What is the CSF sampling site? Please select all that apply.

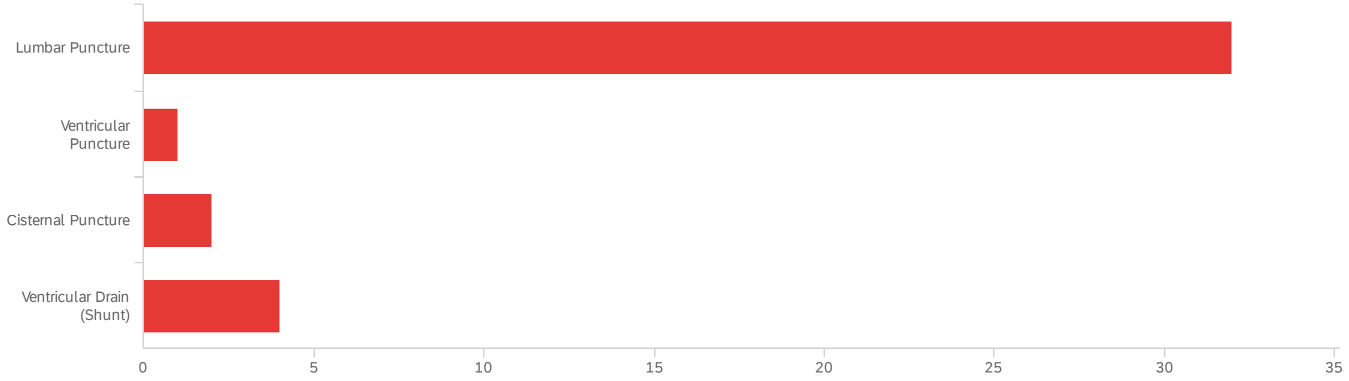

#	Field		Choice Count
1	Lumbar Puncture	82.05%	32
2	Ventricular Puncture	2.56%	1
3	Cisternal Puncture	5.13%	2
6	Ventricular Drain (Shunt)	10.26%	4
			39

Showing rows 1 - 5 of 5

Q4.1 - Q4.1 Other:

Q4.1 Other:
brain -pre-operative sampling after dura opened

Q5#1 - Q5. What parameters might form reporting guidelines for CSF collection? Please rate the importanc... - Click to write Column 1

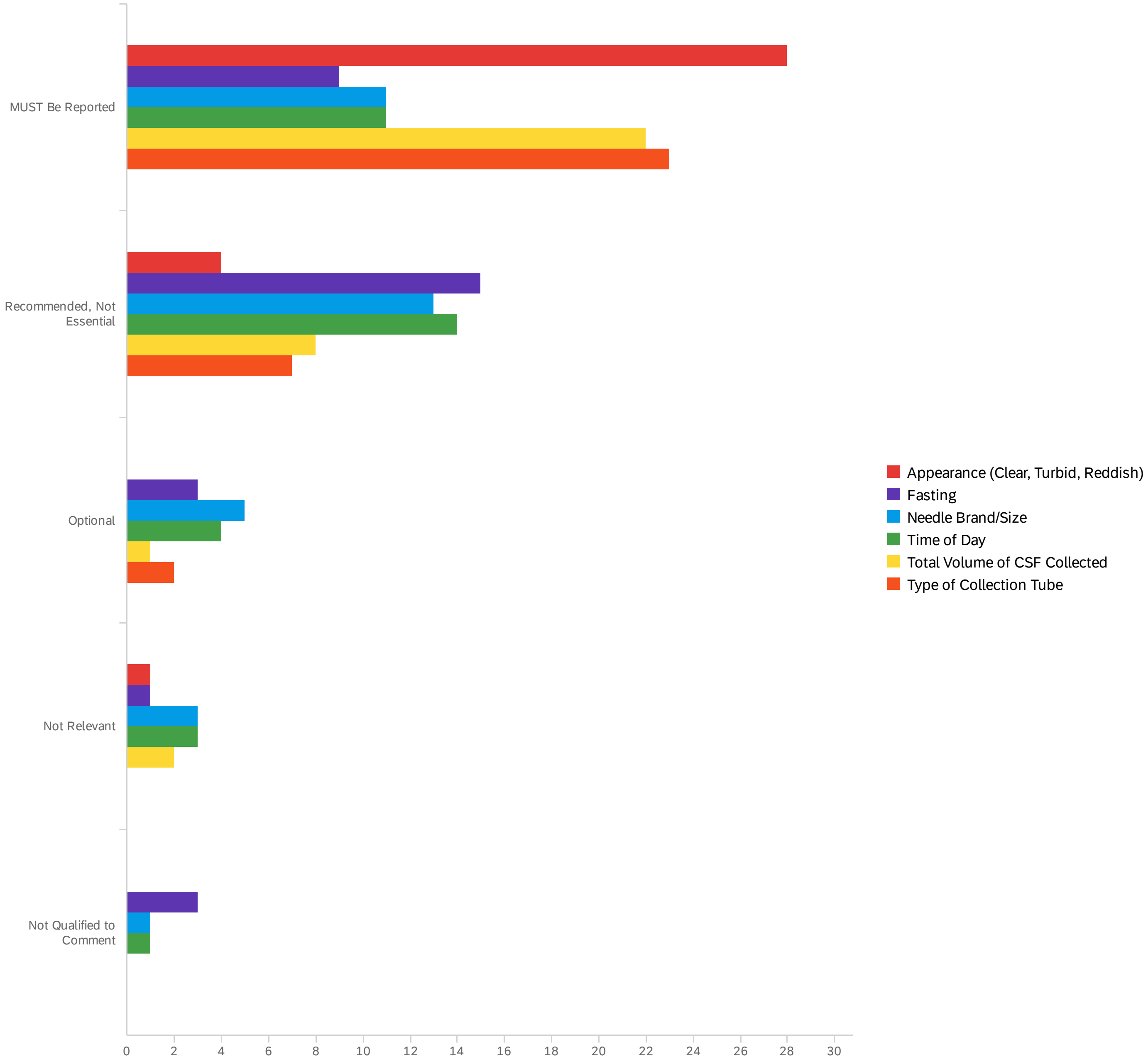

#	Field	Minimum	Maximum	Mean	Std Deviation	Variance	Count
1	Appearance (Clear, Turbid, Reddish)	1.00	4.00	1.21	0.59	0.35	33
2	Fasting	1.00	5.00	2.16	1.17	1.36	31
3	Needle Brand/Size	1.00	5.00	2.09	1.05	1.11	33

#	Field	Minimum	Maximum	Mean	Std Deviation	Variance	Count
4	Time of Day	1.00	5.00	2.06	1.04	1.09	33
5	Total Volume of CSF Collected	1.00	4.00	1.48	0.82	0.67	33
6	Type of Collection Tube	1.00	3.00	1.34	0.59	0.35	32

#	Field	MUST Be Reported		Recommended, Not Essential		Optional		Not Relevant		Not Qualified to Comment		Total
1	Appearance (Clear, Turbid, Reddish)	84.85%	28	12.12%	4	0.00%	0	3.03%	1	0.00%	0	33
2	Fasting	29.03%	9	48.39%	15	9.68%	3	3.23%	1	9.68%	3	31
3	Needle Brand/Size	33.33%	11	39.39%	13	15.15%	5	9.09%	3	3.03%	1	33
4	Time of Day	33.33%	11	42.42%	14	12.12%	4	9.09%	3	3.03%	1	33
5	Total Volume of CSF Collected	66.67%	22	24.24%	8	3.03%	1	6.06%	2	0.00%	0	33
6	Type of Collection Tube	71.88%	23	21.88%	7	6.25%	2	0.00%	0	0.00%	0	32

Showing rows 1 - 6 of 6

Q5.1 - Q5.1 Other:

Q5.1 Other:
It would be ideal to assess parameters by some means beyond visual examination. Like spectroscopy for presence of hemoglobin or measuring specific gravity or protein content.
Anesthesia
When was the CSF sample collected (i.e. first sample after punction, last sample after punction) when sample is taken via Lumbar punction

Q6#1 - Q6. What parameters might form reporting guidelines for CSF post-collection processing and storag... - Click to write Column 1

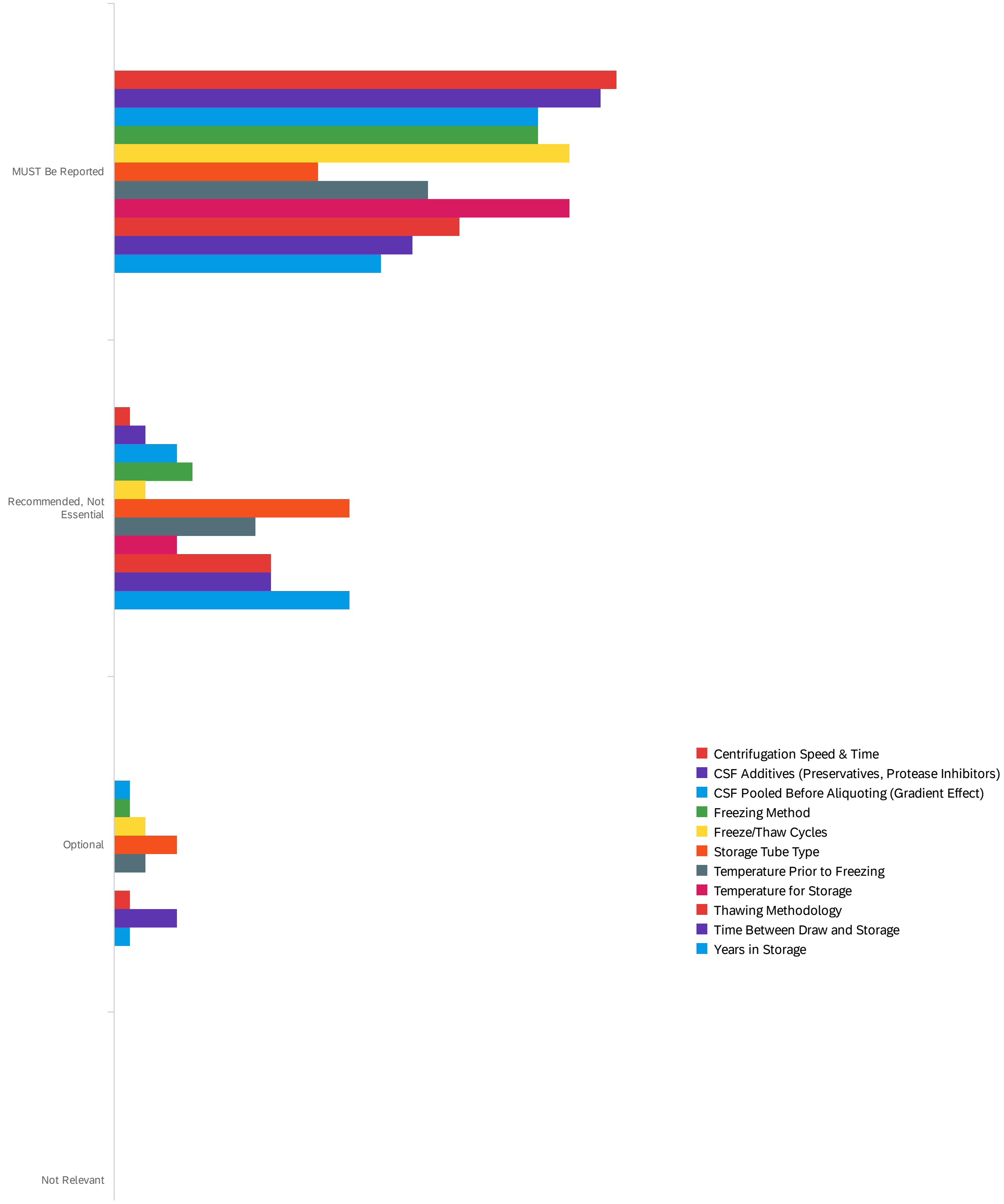

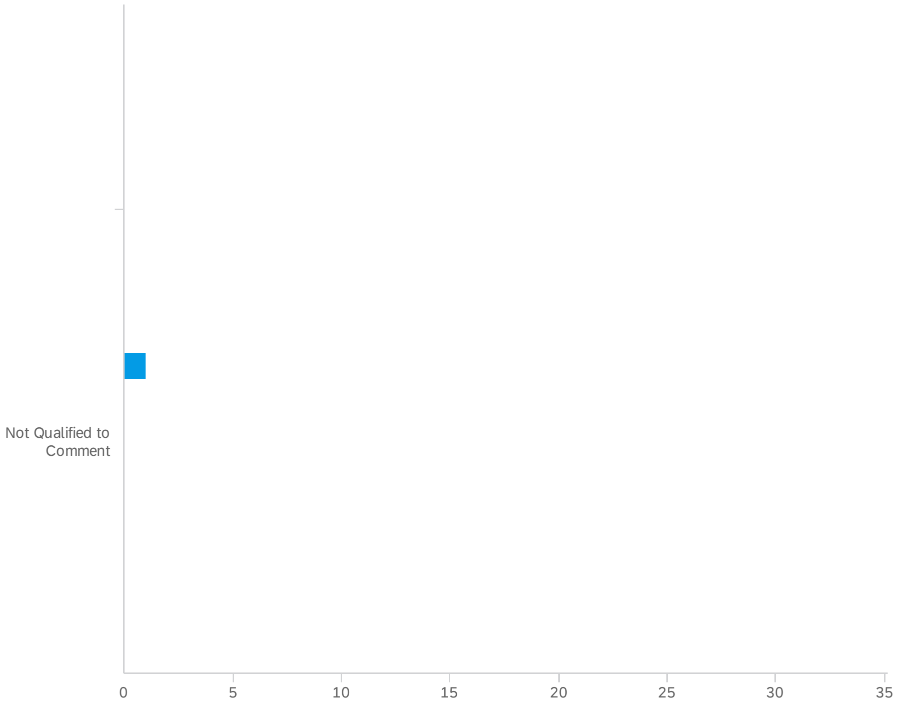

#	Field	Minimum	Maximum	Mean	Std Deviation	Variance	Count
1	Centrifugation Speed & Time	1.00	2.00	1.03	0.17	0.03	33
2	CSF Additives (Preservatives, Protease Inhibitors)	1.00	2.00	1.06	0.24	0.06	33
3	CSF Pooled Before Aliquoting (Gradient Effect)	1.00	5.00	1.30	0.80	0.64	33
4	Freezing Method	1.00	3.00	1.21	0.48	0.23	33
5	Freeze/Thaw Cycles	1.00	3.00	1.18	0.52	0.27	33
6	Storage Tube Type	1.00	3.00	1.72	0.67	0.45	32
7	Temperature Prior to Freezing	1.00	3.00	1.42	0.61	0.37	31
8	Temperature for Storage	1.00	2.00	1.12	0.33	0.11	33
9	Thawing Methodology	1.00	3.00	1.36	0.54	0.29	33
10	Time Between Draw and Storage	1.00	3.00	1.55	0.70	0.49	33
11	Years in Storage	1.00	3.00	1.52	0.56	0.31	33

#	Field	MUST Be Reported	Recommended, Not Essential	Optional	Not Relevant	Not Qualified to Comment	Total
1	Centrifugation Speed & Time	96.97% 32	3.03% 1	0.00% 0	0.00% 0	0.00% 0	33

#	Field	MUST Be Reported		Recommended, Not Essential		Optional		Not Relevant		Not Qualified to Comment		Total
2	CSF Additives (Preservatives, Protease Inhibitors)	93.94%	31	6.06%	2	0.00%	0	0.00%	0	0.00%	0	33
3	CSF Pooled Before Aliquoting (Gradient Effect)	81.82%	27	12.12%	4	3.03%	1	0.00%	0	3.03%	1	33
4	Freezing Method	81.82%	27	15.15%	5	3.03%	1	0.00%	0	0.00%	0	33
5	Freeze/Thaw Cycles	87.88%	29	6.06%	2	6.06%	2	0.00%	0	0.00%	0	33
6	Storage Tube Type	40.63%	13	46.88%	15	12.50%	4	0.00%	0	0.00%	0	32
7	Temperature Prior to Freezing	64.52%	20	29.03%	9	6.45%	2	0.00%	0	0.00%	0	31
8	Temperature for Storage	87.88%	29	12.12%	4	0.00%	0	0.00%	0	0.00%	0	33
9	Thawing Methodology	66.67%	22	30.30%	10	3.03%	1	0.00%	0	0.00%	0	33
10	Time Between Draw and Storage	57.58%	19	30.30%	10	12.12%	4	0.00%	0	0.00%	0	33
11	Years in Storage	51.52%	17	45.45%	15	3.03%	1	0.00%	0	0.00%	0	33

Showing rows 1 - 11 of 11

Q6.1 - Q6.1 Other:

Q6.1 Other:
more information is always better

Q7 - Q7. What markers should be considered as CSF contaminants? Please select all that apply.

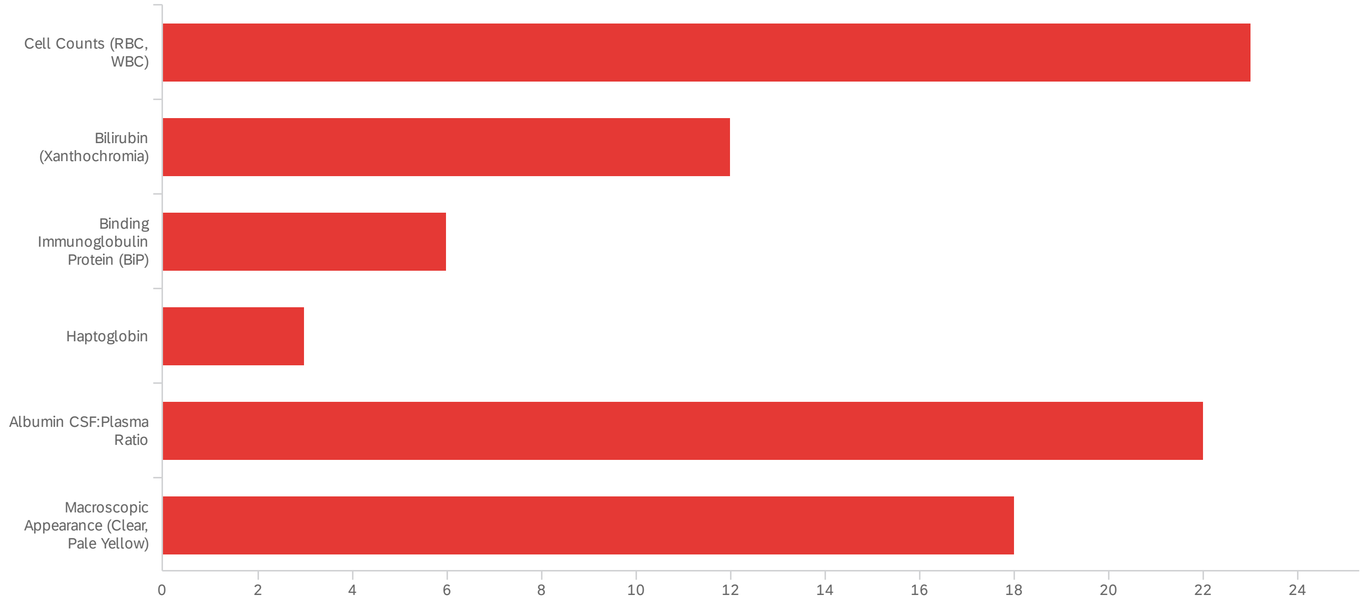

#	Field	Choice Count	
1	Cell Counts (RBC, WBC)	27.38%	23
2	Bilirubin (Xanthochromia)	14.29%	12
3	Binding Immunoglobulin Protein (BiP)	7.14%	6
4	Haptoglobin	3.57%	3
5	Albumin CSF:Plasma Ratio	26.19%	22
7	Macroscopic Appearance (Clear, Pale Yellow)	21.43%	18
			84

Showing rows 1 - 7 of 7

Q7.1 - Q7.1 Other:

Q7.1 Other:
I am not sure how to answer this question, since I was unsure if this meant contaminants of CSF (stuff that isn't supposed to be there) or contaminants of EV preps.
Hemoglobin

Q8 - Q8. What method(s) do you use to fractionate/concentrate your CSF EVs? Please select all that apply.

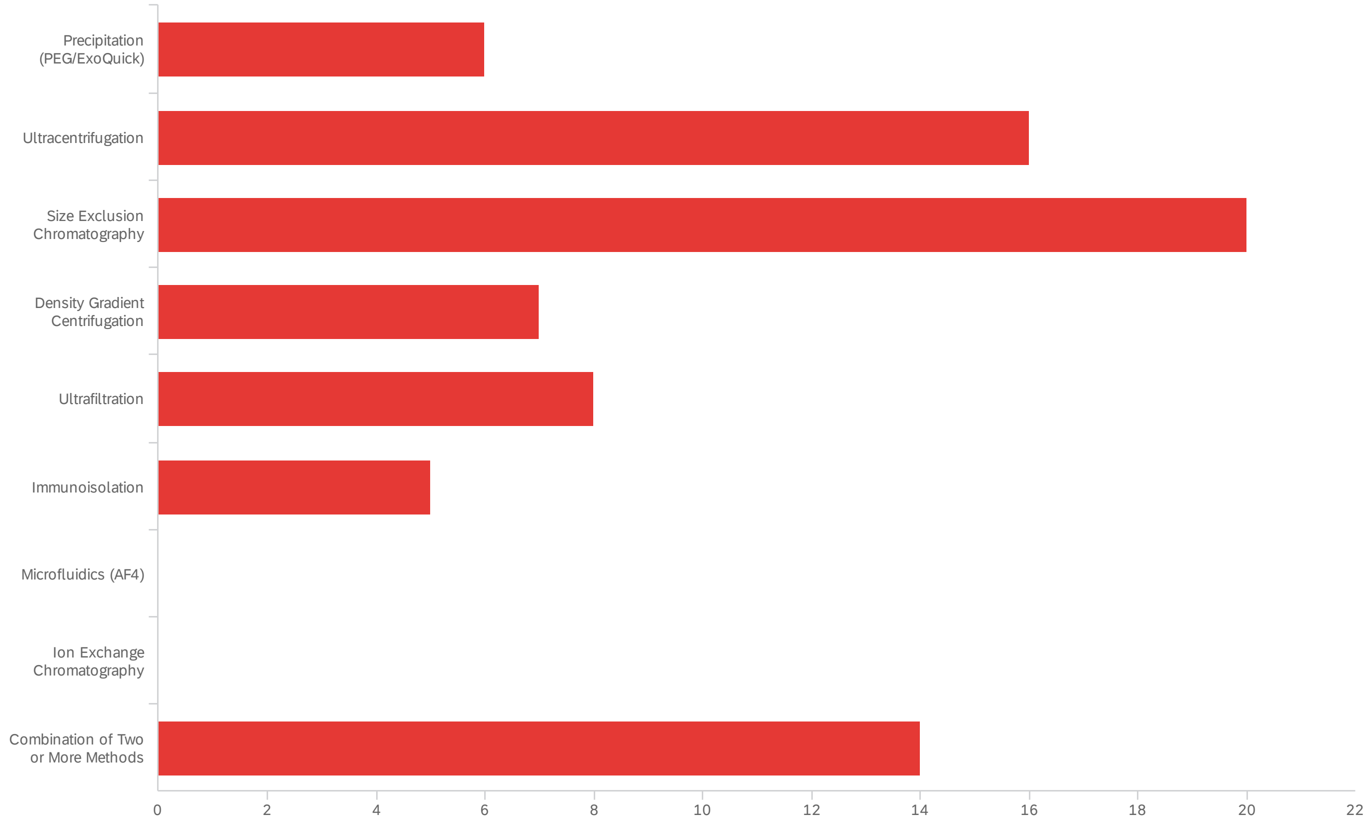

#	Field		Choice Count
1	Precipitation (PEG/ExoQuick)	7.89%	6
2	Ultracentrifugation	21.05%	16
3	Size Exclusion Chromatography	26.32%	20
4	Density Gradient Centrifugation	9.21%	7
5	Ultrafiltration	10.53%	8
6	Immunoisolation	6.58%	5
7	Microfluidics (AF4)	0.00%	0
8	Ion Exchange Chromatography	0.00%	0
9	Combination of Two or More Methods	18.42%	14
			76

Showing rows 1 - 10 of 10

Q8.1 - Q8.1 Other:

Q8.1 Other:
size exclusion chromatography followed by ultrafiltration to concentrate EV fractions
rotovap

Q9 - Q9. Please rate the importance of reporting each of the following parameters.

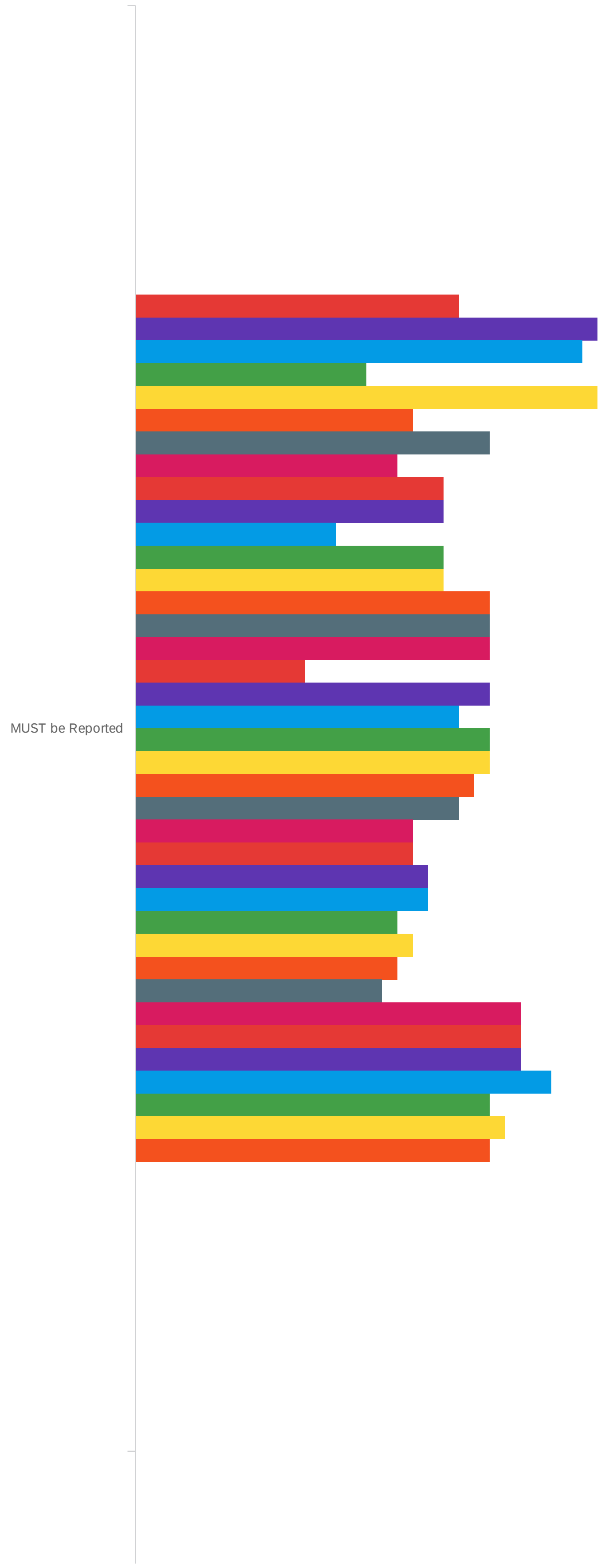

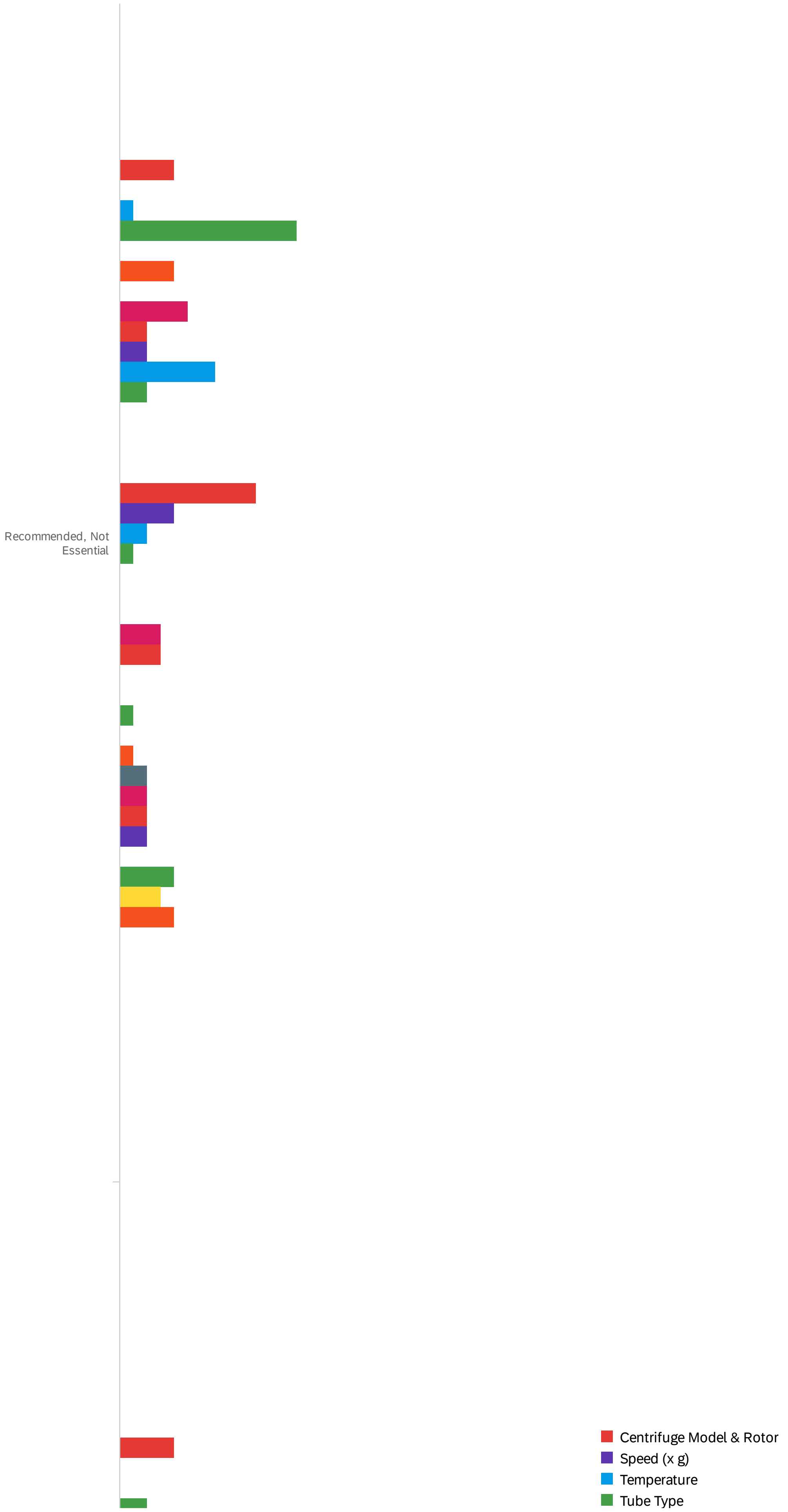

Recommended, Not Essential
Centrifuge Model & Rotor
Speed (x g)
Temperature
Tube Type

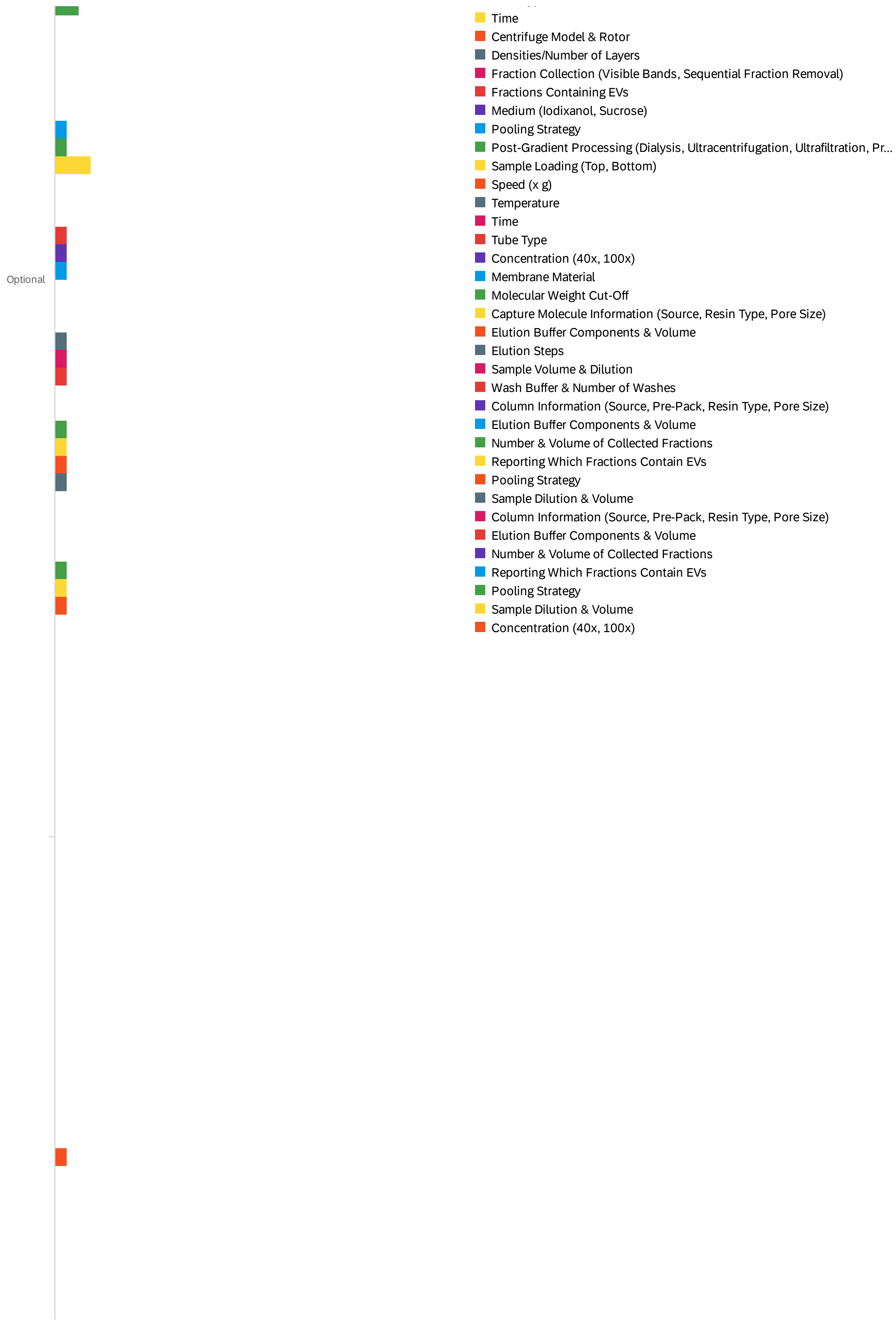
Optional
Time
Centrifuge Model & Rotor
Densities/Number of Layers
Fraction Collection (Visible Bands, Sequential Fraction Removal)
Fractions Containing EVs
Medium (Iodixanol, Sucrose)
Pooling Strategy
Post-Gradient Processing (Dialysis, Ultracentrifugation, Ultrafiltration, Pr...
Sample Loading (Top, Bottom)
Speed (x g)
Temperature
Time
Tube Type
Concentration (40x, 100x)
Membrane Material
Molecular Weight Cut-Off
Capture Molecule Information (Source, Resin Type, Pore Size)
Elution Buffer Components & Volume
Elution Steps
Sample Volume & Dilution
Wash Buffer & Number of Washes
Column Information (Source, Pre-Pack, Resin Type, Pore Size)
Elution Buffer Components & Volume
Number & Volume of Collected Fractions
Reporting Which Fractions Contain EVs
Pooling Strategy
Sample Dilution & Volume
Column Information (Source, Pre-Pack, Resin Type, Pore Size)
Elution Buffer Components & Volume
Number & Volume of Collected Fractions
Reporting Which Fractions Contain EVs
Pooling Strategy
Sample Dilution & Volume
Concentration (40x, 100x)

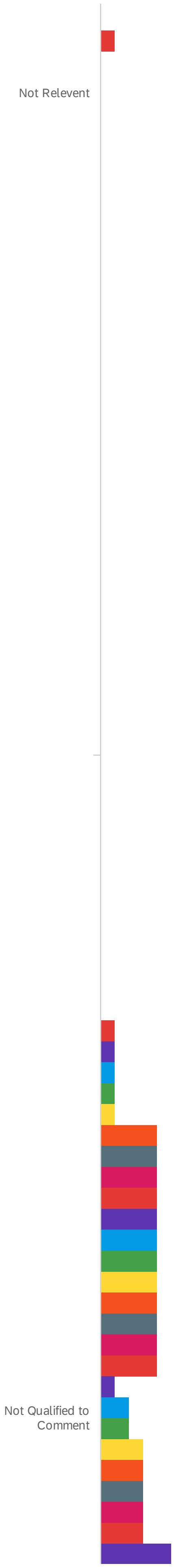

Not Relevent
Not Qualified to Comment

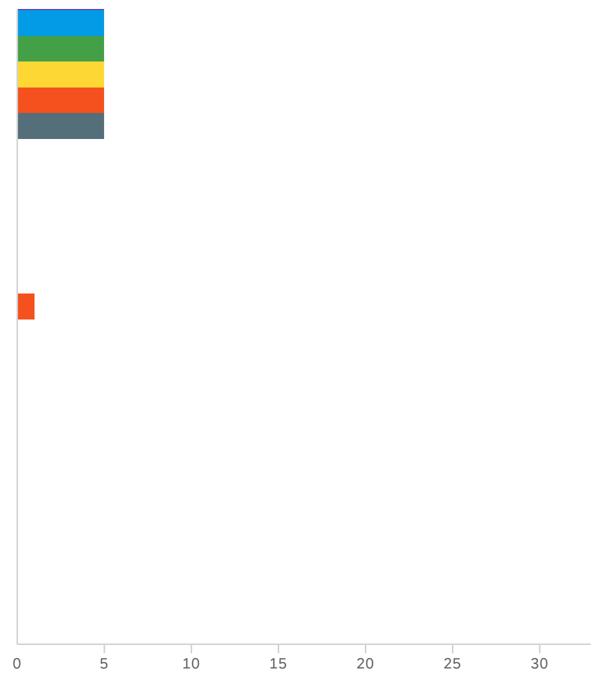

#	Field	Minimum	Maximum	Mean	Std Deviation	Variance	Count
1	Centrifuge Model & Rotor	3.00	7.00	3.53	0.96	0.92	30
2	Speed (x g)	3.00	7.00	3.13	0.71	0.50	31
3	Temperature	3.00	7.00	3.16	0.72	0.52	31
4	Tube Type	3.00	7.00	3.68	0.86	0.73	31
5	Time	3.00	7.00	3.13	0.71	0.50	31
6	Centrifuge Model & Rotor	3.00	7.00	3.85	1.46	2.13	27
7	Densities/Number of Layers	3.00	7.00	3.59	1.42	2.02	27
8	Fraction Collection (Visible Bands, Sequential Fraction Removal)	3.00	7.00	3.81	1.41	2.00	26
9	Fractions Containing EVs	3.00	7.00	3.69	1.43	2.06	26
10	Medium (Iodixanol, Sucrose)	3.00	7.00	3.69	1.43	2.06	26
11	Pooling Strategy	3.00	7.00	4.00	1.41	2.00	25
12	Post-Gradient Processing (Dialysis, Ultracentrifugation, Ultrafiltration, Precipitation)	3.00	7.00	3.74	1.43	2.04	27
13	Sample Loading (Top, Bottom)	3.00	7.00	3.81	1.47	2.15	27
14	Speed (x g)	3.00	7.00	3.59	1.42	2.02	27

#	Field	Minimum	Maximum	Mean	Std Deviation	Variance	Count
15	Temperature	3.00	7.00	3.59	1.42	2.02	27
16	Time	3.00	7.00	3.59	1.42	2.02	27
17	Tube Type	3.00	7.00	4.15	1.38	1.90	27
38	Concentration (40x, 100x)	3.00	7.00	3.34	0.84	0.71	29
19	Membrane Material	3.00	7.00	3.46	1.12	1.25	26
20	Molecular Weight Cut-Off	3.00	7.00	3.35	1.07	1.15	26
21	Capture Molecule Information (Source, Resin Type, Pore Size)	3.00	7.00	3.46	1.28	1.63	26
22	Elution Buffer Components & Volume	3.00	7.00	3.48	1.30	1.69	25
23	Elution Steps	3.00	7.00	3.56	1.33	1.77	25
24	Sample Volume & Dilution	3.00	7.00	3.68	1.32	1.74	25
25	Wash Buffer & Number of Washes	3.00	7.00	3.68	1.32	1.74	25
26	Column Information (Source, Pre-Pack, Resin Type, Pore Size)	3.00	7.00	3.83	1.62	2.64	24
27	Elution Buffer Components & Volume	3.00	7.00	3.83	1.62	2.64	24
28	Number & Volume of Collected Fractions	3.00	7.00	3.96	1.62	2.62	24
29	Reporting Which Fractions Contain EVs	3.00	7.00	3.92	1.63	2.66	24
30	Pooling Strategy	3.00	7.00	3.96	1.62	2.62	24
31	Sample Dilution & Volume	3.00	7.00	4.00	1.61	2.58	24
32	Column Information (Source, Pre-Pack, Resin Type, Pore Size)	3.00	4.00	3.07	0.26	0.07	27
33	Elution Buffer Components & Volume	3.00	4.00	3.07	0.26	0.07	27
34	Number & Volume of Collected Fractions	3.00	4.00	3.07	0.26	0.07	27
35	Reporting Which Fractions Contain EVs	3.00	3.00	3.00	0.00	0.00	27
36	Pooling Strategy	3.00	5.00	3.21	0.49	0.24	28
37	Sample Dilution & Volume	3.00	5.00	3.18	0.47	0.22	28
38	Concentration (40x, 100x)	3.00	7.00	3.34	0.84	0.71	29

#	Field	MUST be Reported		Recommended, Not Essential		Optional		Not Relevent		Not Qualified to Comment		Total
1	Centrifuge Model & Rotor	70.00%	21	13.33%	4	13.33%	4	0.00%	0	3.33%	1	30
2	Speed (x g)	96.77%	30	0.00%	0	0.00%	0	0.00%	0	3.23%	1	31
3	Temperature	93.55%	29	3.23%	1	0.00%	0	0.00%	0	3.23%	1	31
4	Tube Type	48.39%	15	41.94%	13	6.45%	2	0.00%	0	3.23%	1	31
5	Time	96.77%	30	0.00%	0	0.00%	0	0.00%	0	3.23%	1	31
6	Centrifuge Model & Rotor	66.67%	18	14.81%	4	0.00%	0	3.70%	1	14.81%	4	27
7	Densities/Number of Layers	85.19%	23	0.00%	0	0.00%	0	0.00%	0	14.81%	4	27
8	Fraction Collection (Visible Bands, Sequential Fraction Removal)	65.38%	17	19.23%	5	0.00%	0	0.00%	0	15.38%	4	26
9	Fractions Containing EVs	76.92%	20	7.69%	2	0.00%	0	0.00%	0	15.38%	4	26
10	Medium (Iodixanol, Sucrose)	76.92%	20	7.69%	2	0.00%	0	0.00%	0	15.38%	4	26
11	Pooling Strategy	52.00%	13	28.00%	7	4.00%	1	0.00%	0	16.00%	4	25
12	Post-Gradient Processing (Dialysis, Ultracentrifugation, Ultrafiltration, Precipitation)	74.07%	20	7.41%	2	3.70%	1	0.00%	0	14.81%	4	27
13	Sample Loading (Top, Bottom)	74.07%	20	0.00%	0	11.11%	3	0.00%	0	14.81%	4	27
14	Speed (x g)	85.19%	23	0.00%	0	0.00%	0	0.00%	0	14.81%	4	27
15	Temperature	85.19%	23	0.00%	0	0.00%	0	0.00%	0	14.81%	4	27
16	Time	85.19%	23	0.00%	0	0.00%	0	0.00%	0	14.81%	4	27
17	Tube Type	40.74%	11	37.04%	10	3.70%	1	3.70%	1	14.81%	4	27
18	Concentration (40x, 100x)	79.31%	23	13.79%	4	3.45%	1	0.00%	0	3.45%	1	29
19	Membrane Material	80.77%	21	7.69%	2	3.85%	1	0.00%	0	7.69%	2	26
20	Molecular Weight Cut-Off	88.46%	23	3.85%	1	0.00%	0	0.00%	0	7.69%	2	26
21	Capture Molecule Information (Source, Resin Type, Pore Size)	88.46%	23	0.00%	0	0.00%	0	0.00%	0	11.54%	3	26
22	Elution Buffer Components & Volume	88.00%	22	0.00%	0	0.00%	0	0.00%	0	12.00%	3	25
23	Elution Steps	84.00%	21	0.00%	0	4.00%	1	0.00%	0	12.00%	3	25
24	Sample Volume & Dilution	72.00%	18	12.00%	3	4.00%	1	0.00%	0	12.00%	3	25
25	Wash Buffer & Number of Washes	72.00%	18	12.00%	3	4.00%	1	0.00%	0	12.00%	3	25

#	Field	MUST be Reported		Recommended, Not Essential		Optional		Not Relevent		Not Qualified to Comment		Total
26	Column Information (Source, Pre-Pack, Resin Type, Pore Size)	79.17%	19	0.00%	0	0.00%	0	0.00%	0	20.83%	5	24
27	Elution Buffer Components & Volume	79.17%	19	0.00%	0	0.00%	0	0.00%	0	20.83%	5	24
28	Number & Volume of Collected Fractions	70.83%	17	4.17%	1	4.17%	1	0.00%	0	20.83%	5	24
29	Reporting Which Fractions Contain EVs	75.00%	18	0.00%	0	4.17%	1	0.00%	0	20.83%	5	24
30	Pooling Strategy	70.83%	17	4.17%	1	4.17%	1	0.00%	0	20.83%	5	24
31	Sample Dilution & Volume	66.67%	16	8.33%	2	4.17%	1	0.00%	0	20.83%	5	24
32	Column Information (Source, Pre-Pack, Resin Type, Pore Size)	92.59%	25	7.41%	2	0.00%	0	0.00%	0	0.00%	0	27
33	Elution Buffer Components & Volume	92.59%	25	7.41%	2	0.00%	0	0.00%	0	0.00%	0	27
34	Number & Volume of Collected Fractions	92.59%	25	7.41%	2	0.00%	0	0.00%	0	0.00%	0	27
35	Reporting Which Fractions Contain EVs	100.00%	27	0.00%	0	0.00%	0	0.00%	0	0.00%	0	27
36	Pooling Strategy	82.14%	23	14.29%	4	3.57%	1	0.00%	0	0.00%	0	28
37	Sample Dilution & Volume	85.71%	24	10.71%	3	3.57%	1	0.00%	0	0.00%	0	28
38	Concentration (40x, 100x)	79.31%	23	13.79%	4	3.45%	1	0.00%	0	3.45%	1	29

Showing rows 1 - 38 of 38

Q9.1 - Q9.1 Other:

Q9.1 Other:
All!

Q10 - Q10. What is the minimal starting volume of CSF needed for your fractionation method(s)?

Q10. What is the minimal starting volume of CSF needed for your fractionati...
500 ul
100-500uL
100ul
need to be found out
200 -500 micro liter
There is no single answer. For a high-abundance RNA target, we might need only 50 or 100 ul. For some downstream applications, several mL are needed or even pooling of multiple samples.
1 mL
100ul is the absolute minimum, we prefer 250ul
1 ml
0,5 ml
500µl
500µL
1 mL
3 ml
500ul
200 µL
0.2 ml
i recall we had useable material from 2-3 ml csf
1ml
50 ul

Q10. What is the minimal starting volume of CSF needed for your fractionati...

3 ml
0.5 mL
500 microliters
500 uL
500 ul for SEC if downstream application is qPCR. 5mLs CSF for SEC for WB, TEM.

Q11#1 - Q11. What parameters might form reporting guidelines for post-fractionation storage of CSF EV pre... - Click to write Column 1

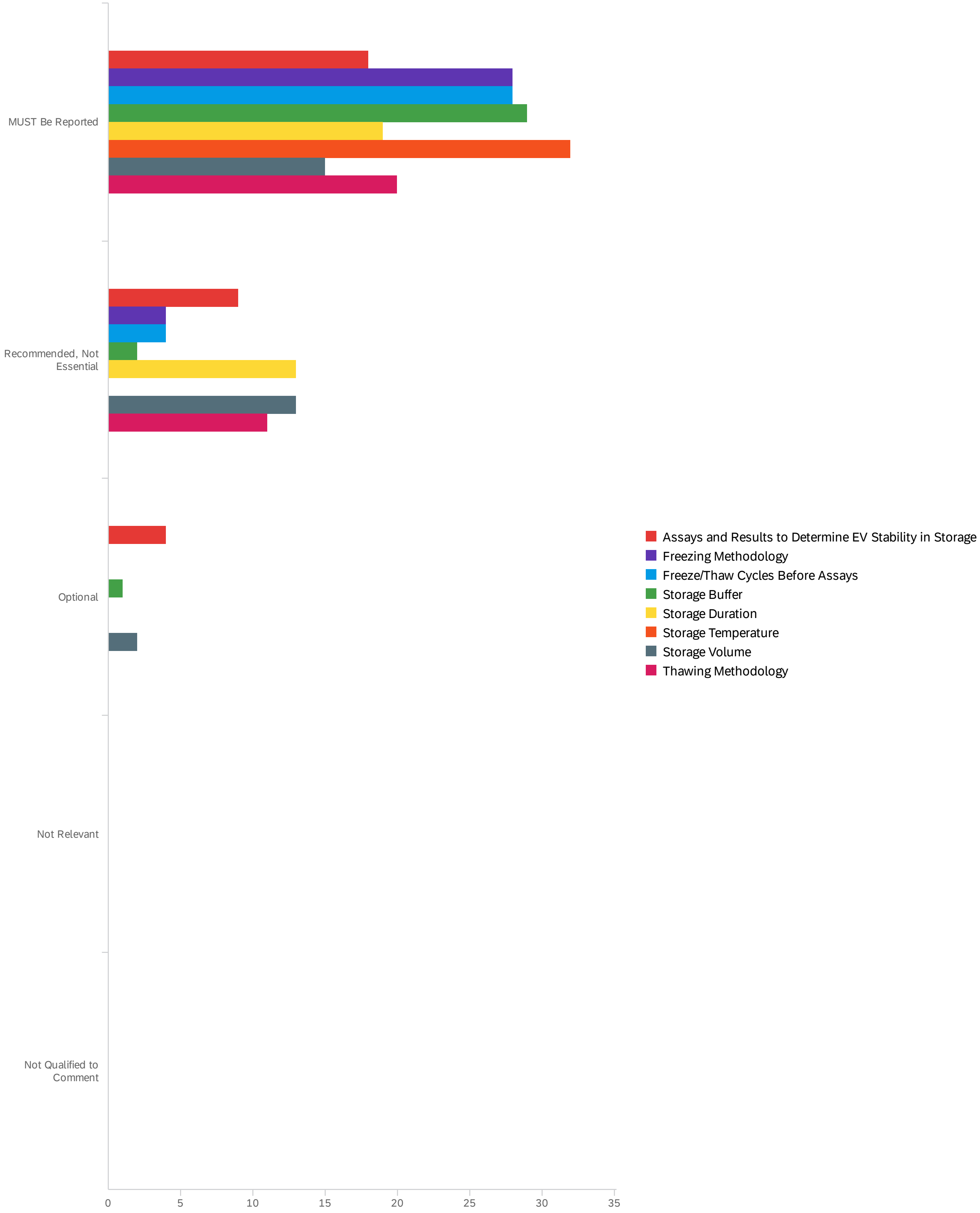

#	Field	Minimum	Maximum	Mean	Std Deviation	Variance	Count
1	Assays and Results to Determine EV Stability in Storage	1.00	3.00	1.55	0.71	0.51	31

#	Field	Minimum	Maximum	Mean	Std Deviation	Variance	Count
2	Freezing Methodology	1.00	2.00	1.13	0.33	0.11	32
3	Freeze/Thaw Cycles Before Assays	1.00	2.00	1.13	0.33	0.11	32
4	Storage Buffer	1.00	3.00	1.13	0.41	0.17	32
5	Storage Duration	1.00	2.00	1.41	0.49	0.24	32
6	Storage Temperature	1.00	1.00	1.00	0.00	0.00	32
7	Storage Volume	1.00	3.00	1.57	0.62	0.38	30
8	Thawing Methodology	1.00	2.00	1.35	0.48	0.23	31

#	Field	MUST Be Reported		Recommended, Not Essential		Optional		Not Relevant		Not Qualified to Comment		Total
1	Assays and Results to Determine EV Stability in Storage	58.06%	18	29.03%	9	12.90%	4	0.00%	0	0.00%	0	31
2	Freezing Methodology	87.50%	28	12.50%	4	0.00%	0	0.00%	0	0.00%	0	32
3	Freeze/Thaw Cycles Before Assays	87.50%	28	12.50%	4	0.00%	0	0.00%	0	0.00%	0	32
4	Storage Buffer	90.63%	29	6.25%	2	3.13%	1	0.00%	0	0.00%	0	32
5	Storage Duration	59.38%	19	40.63%	13	0.00%	0	0.00%	0	0.00%	0	32
6	Storage Temperature	100.00%	32	0.00%	0	0.00%	0	0.00%	0	0.00%	0	32
7	Storage Volume	50.00%	15	43.33%	13	6.67%	2	0.00%	0	0.00%	0	30
8	Thawing Methodology	64.52%	20	35.48%	11	0.00%	0	0.00%	0	0.00%	0	31

Showing rows 1 - 8 of 8

Q11.1 - Q11.1 Other:

Q11.1 Other:
I think that EVs are fairly stable during storage, although many would disagree with that.

Q12 - Q12. What methods do you use for analyzing CSF EV size and/or concentration?

Please select all that apply.

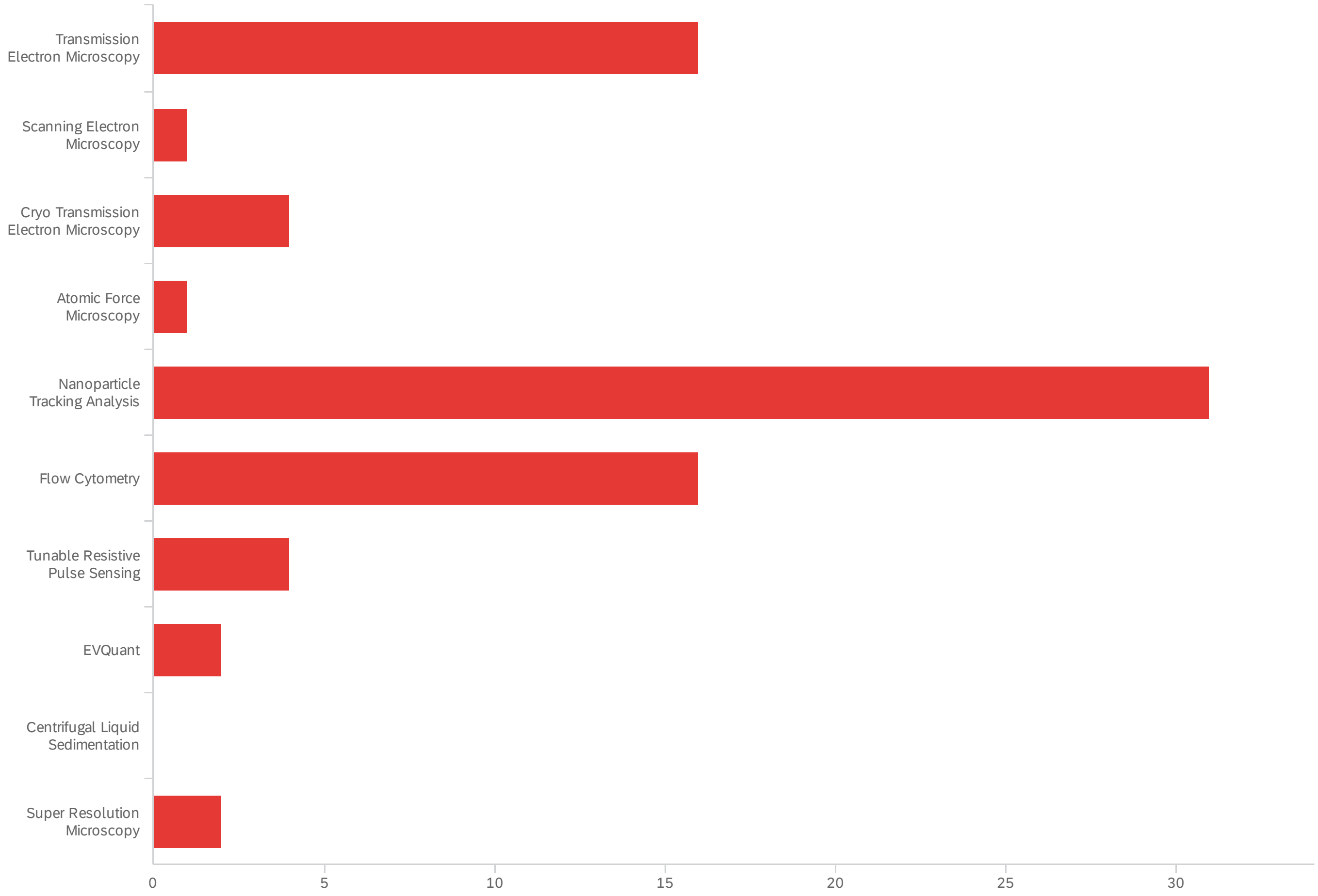

#	Field	Choice Count	
1	Transmission Electron Microscopy	20.78%	16
2	Scanning Electron Microscopy	1.30%	1
3	Cryo Transmission Electron Microscopy	5.19%	4
4	Atomic Force Microscopy	1.30%	1
6	Nanoparticle Tracking Analysis	40.26%	31
9	Flow Cytometry	20.78%	16
10	Tunable Resistive Pulse Sensing	5.19%	4
12	EVQuant	2.60%	2
17	Centrifugal Liquid Sedimentation	0.00%	0

#	Field		Choice Count
18	Super Resolution Microscopy	2.60%	2
			77

Showing rows 1 - 11 of 11

Q12.1 - Q12.1 Other:

Q12.1 Other:
Zetaview particle counts
AF4,
DLS
immuno-phenotyping assays
MRPS, multiplex

Q13 - Q13. What is the minimal starting volume of CSF needed for your EV size and/or concentration methods?

Q13. What is the minimal starting volume of CSF needed for your EV size and...
0.8 ml
5-10uL
5-10ul
10ul
not yet determined
200-500
Up to several mL
200ul
Depending on the concentration, volume of EV fraction needed: NTA: 10-100 µL Flow cytometry: 25-100 µL
5-8 ML
10 microliters
500µl
500µL
500UL
1 mL
3 ml
500ul
if measuring direct on CSF 200ul is plenty
1ml
50 ul
3 ml

Q13. What is the minimal starting volume of CSF needed for your EV size and...

5 mL
0.5 mL
500
5 mL
For fNTA and TEM 5mL CSF prior to SEC, 500 ul for flow cytometry.

Q14 - Q14. What method do you use for EV RNA cargo analysis? Please select all that apply.

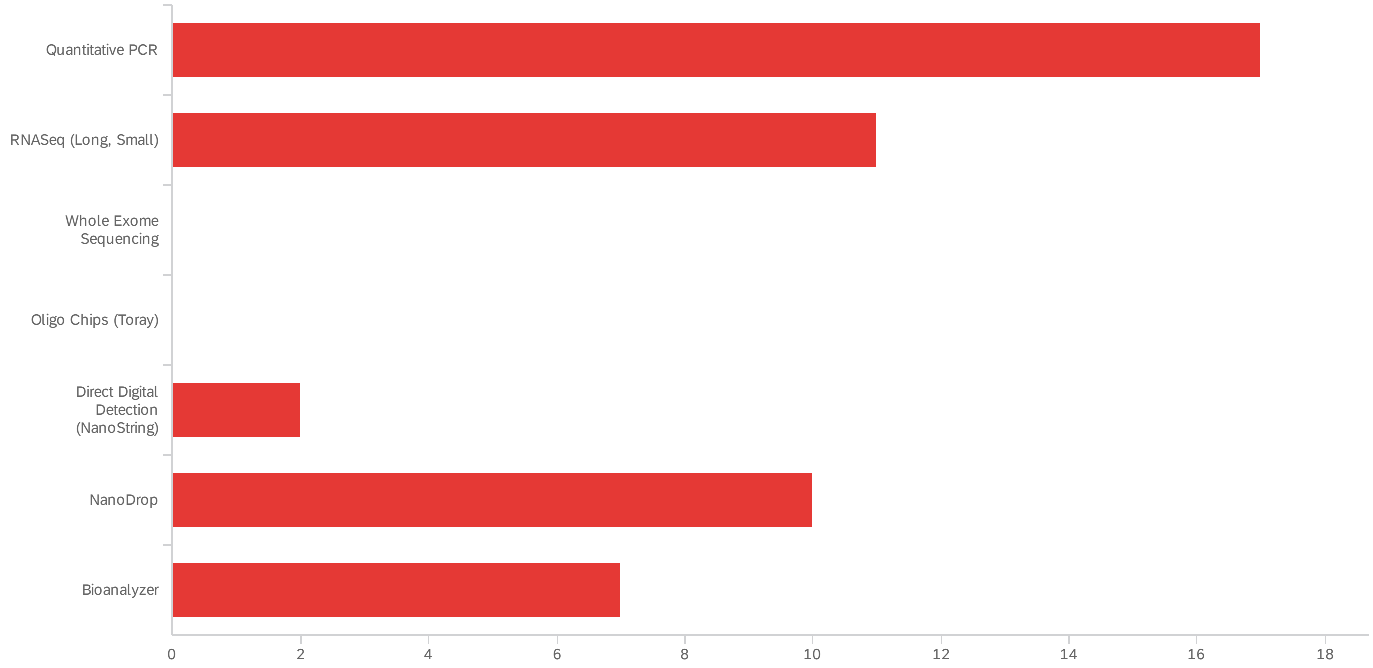

#	Field		Choice Count
1	Quantitative PCR	36.17%	17
2	RNASeq (Long, Small)	23.40%	11
4	Whole Exome Sequencing	0.00%	0
5	Oligo Chips (Toray)	0.00%	0
7	Direct Digital Detection (NanoString)	4.26%	2
10	NanoDrop	21.28%	10
11	Bioanalyzer	14.89%	7
			47

Showing rows 1 - 8 of 8

Q14.1 - Q14.1 Other:

Q14.1 Other:
surface cargo - flow cytometry
to be determined
Qubit
I do proteomics

Q15 - Q15. What is the minimal starting volume for your EV RNA analysis method?

Q15. What is the minimal starting volume for your EV RNA analysis method?
0.5 ml
5-10uL
10-20ul
250ul
to be determined
200-500 micro liter
Ranges from tens of microliters (single qPCR) to many mL (sequencing).
200ul
2-5 µL
5ml
200 microliters
500µL
500ul
we have not yet managed this with CSF
1ml
3 ml
5mL
Subjective
50
500 uL
500ul prior to qPCR.

Q16#1 - Q16. What parameters might form reporting guidelines for CSF EV RNA Cargo analysis? Please rate t... - Click to write Column 1

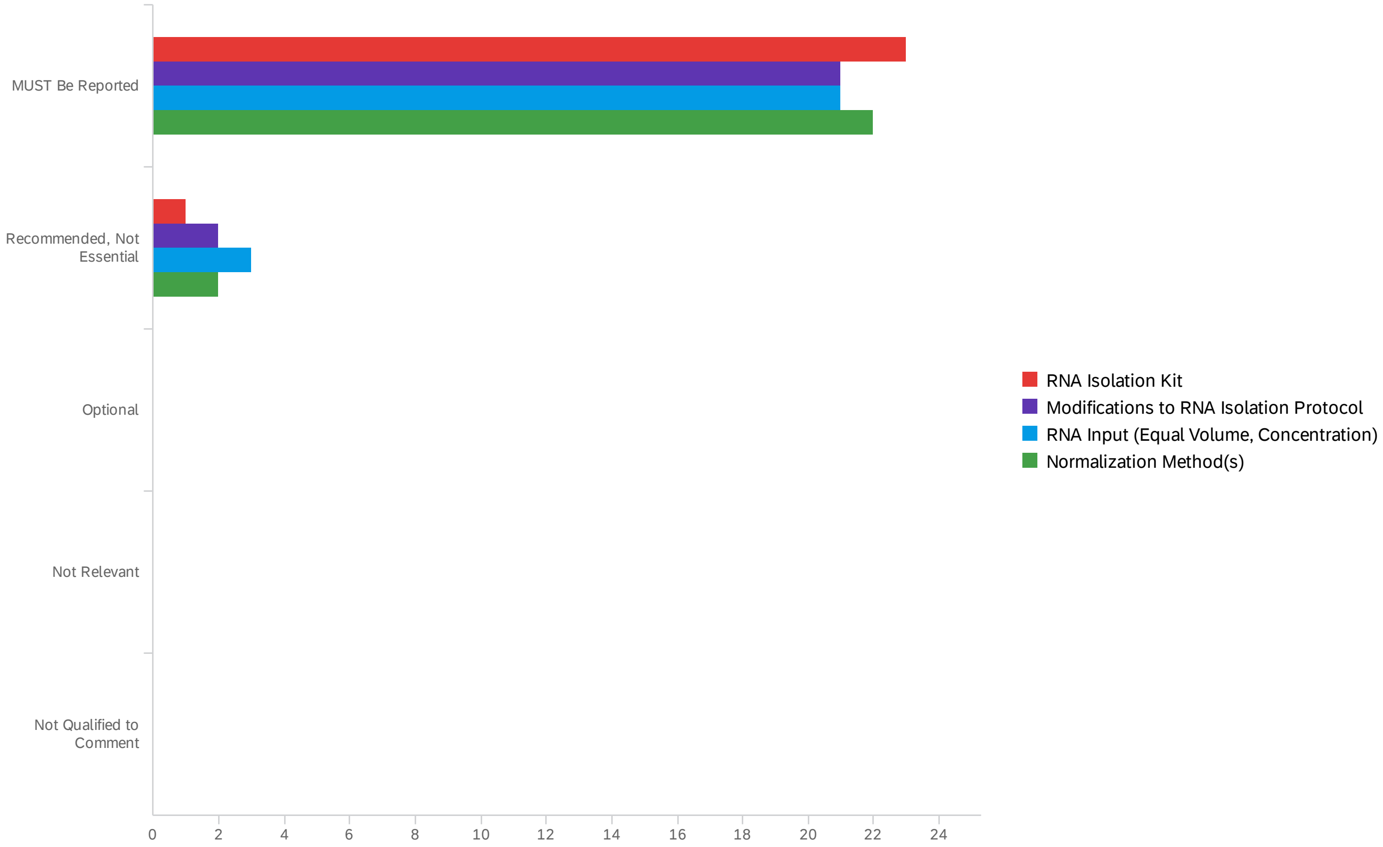

#	Field	Minimum	Maximum	Mean	Std Deviation	Variance	Count
1	RNA Isolation Kit	1.00	2.00	1.04	0.20	0.04	24
2	Modifications to RNA Isolation Protocol	1.00	2.00	1.09	0.28	0.08	23
3	RNA Input (Equal Volume, Concentration)	1.00	2.00	1.13	0.33	0.11	24
4	Normalization Method(s)	1.00	2.00	1.08	0.28	0.08	24

#	Field	MUST Be Reported		Recommended, Not Essential		Optional		Not Relevant		Not Qualified to Comment		Total
1	RNA Isolation Kit	95.83%	23	4.17%	1	0.00%	0	0.00%	0	0.00%	0	24
2	Modifications to RNA Isolation Protocol	91.30%	21	8.70%	2	0.00%	0	0.00%	0	0.00%	0	23

#	Field	MUST Be Reported		Recommended, Not Essential		Optional		Not Relevant		Not Qualified to Comment		Total
3	RNA Input (Equal Volume, Concentration)	87.50%	21	12.50%	3	0.00%	0	0.00%	0	0.00%	0	24
4	Normalization Method(s)	91.67%	22	8.33%	2	0.00%	0	0.00%	0	0.00%	0	24

Showing rows 1 - 4 of 4

Q16.1 - Q16.1 Other:

Q16.1 Other:
If the goal is to measure EV RNA, it must be shown that the RNA is in or attached to the EVs. In many studies, it cannot be excluded that most RNA is not associated with EVs and is simply a co-isolate. I often worry that even a tiny influence of cells (including those coming from the puncture) might contribute more RNA to CSF "EV" measurements than the actual CSF EVs.
lysis procedure, buffers used, detection dye/kit, cDNA synthesis step, amplification method, primer/probe sequences used, etc

Q17 - Q17. What method do you use for CSF EV protein analysis? Please check all that apply.

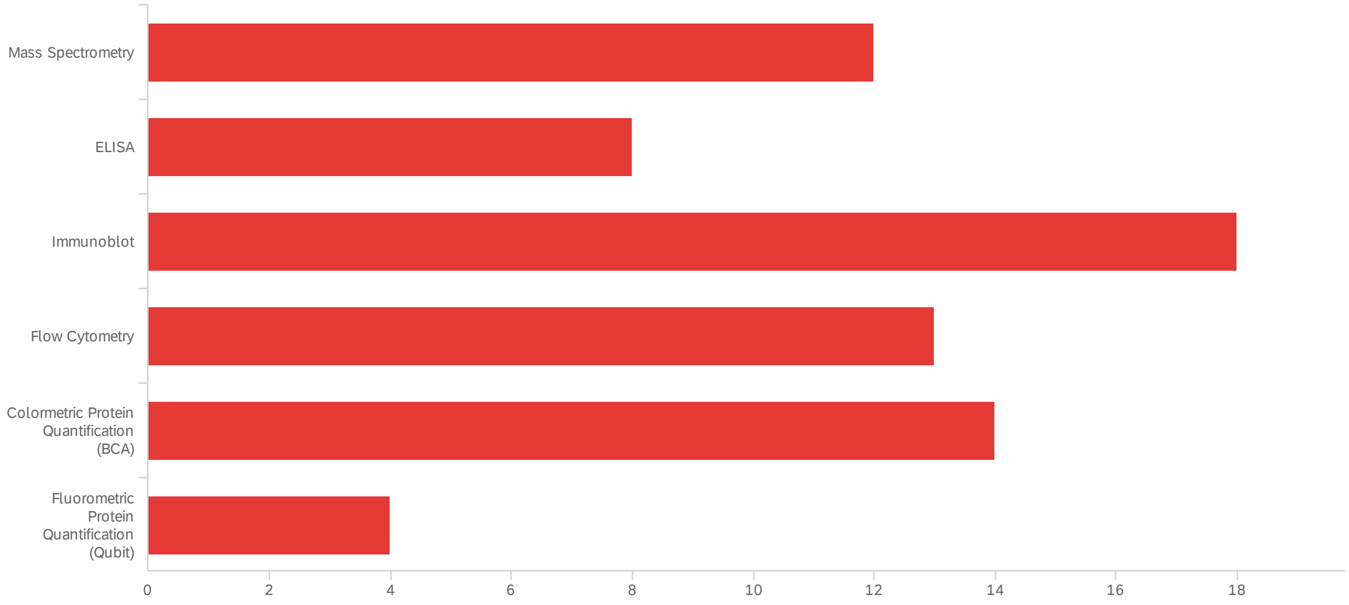

#	Field		Choice Count
1	Mass Spectrometry	17.39%	12
3	ELISA	11.59%	8
4	Immunoblot	26.09%	18
6	Flow Cytometry	18.84%	13
8	Colormetric Protein Quantification (BCA)	20.29%	14
9	Fluorometric Protein Quantification (Qubit)	5.80%	4
			69

Showing rows 1 - 7 of 7

Q17.1 - Q17.1 Other:

Q17.1 Other:
to be determined
fluoNTA, Nanoview
Nanoview ExoView
Multiplex

Q18 - Q18. What non-EV markers do you use for CSF EV protein analysis and/or purity?

Please select all that apply.

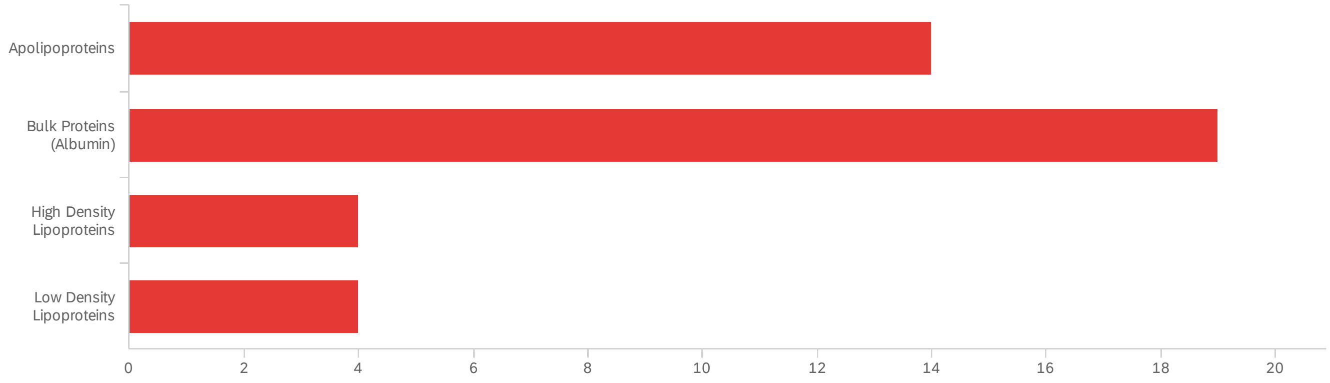

#	Field	Choice Count	
1	Apolipoproteins	34.15%	14
2	Bulk Proteins (Albumin)	46.34%	19
3	High Density Lipoproteins	9.76%	4
4	Low Density Lipoproteins	9.76%	4
			41

Showing rows 1 - 5 of 5

Q18.1 - Q18.1 Other:

Q18.1 Other:
histone 4
IgG

Q19 - Q19. What is the minimal starting volume of CSF for your EV protein analysis?

Q19. What is the minimal starting volume of CSF for your EV protein analysi...
10-20ul
250ul
TBD
200-500 micro liter
1 mL
0.5 ml CSF
2ML
400 microliters
500µl
500µL
1 mL
3 ml
500ul
200µL
0.5 ml
as for our purificaion 1-2mls
1ml
100
3 ml
5mL
0.5 mL
500

Q19. What is the minimal starting volume of CSF for your EV protein analysi...

5 mL
5mL for Immunoblot, 500ul for flow cytometry.

Q20 - Q20. What neuronal and/or glial EV markers do you use in CSF studies?

Q20. What neuronal and/or glial EV markers do you use in CSF studies?
Anti Mog
under investigation
syn
MMP2 and EMMPRIN but these are specific to our disease of interest
L1CAM, NCAM, CD11b
Neurofilament light protein and GFAP
L1CAM, NCAM
NCAM, L1CAM, GLAST
ICAM
L1CAM CD63 CD9
L1CAM (neurons)
NCAM, GFAP, Vimentin
proprietary astrocytoma antigen markers and astrocytoma-associated miRs
L1CAM, TMEM119
L1CAM
NSE, Synaptophysin
Yet to find a neuro-specifc marker
Glast, NCAM
Glast, NCAM

Q21 - Q21. What antibodies (and source) do you recommend for CSF EV positive & negative protein markers?

Q21. What antibodies (and source) do you recommend for CSF EV positive & ne...
TBD
cell signalling
positive: CD81, CD9, CD63 - Santa Cruz (western blot), Biolegend (flow cytometry) negative: ApoA-I - Santa Cruz
ongoing research
Positive: CD9, CD63, CD81 Negative: Albumin
positive: tetraspanins (abcam), NCAM (abcam) , negative: calnexin (abcam)
ITGB1, IGF-1R, IGF-2R for positive, source variable
CD9, CD63, CD81, TSG101, Flotillin, APOA1, Albumin Novus antibodies
ApoA1, Santa Cruz, (12C8) 80551; Albumin, Cell Signaling, #4929; Flotillin, Abcam, AB133497; CD81, Santa Cruz, (B-11) sc-166029; GLAST, Novus, NB100-1869

Q22 - Q22. What other methods do you use to analyze EV components? Please select all that apply.

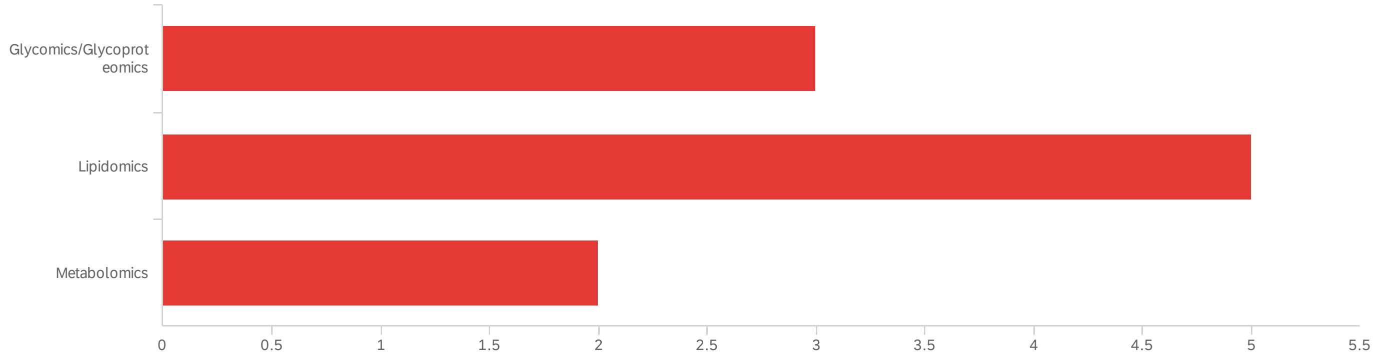

#	Field		Choice Count
1	Glycomics/Glycoproteomics	30.00%	3
2	Lipidomics	50.00%	5
3	Metabolomics	20.00%	2
			10

Showing rows 1 - 4 of 4

Q22.1 - Q22.1 Other:

Q22.1 Other:
we aim for lipidomics and metabolimics
Immunomodulatory assays
proteomics !
proteomics MS/MS

Q23 - Q23. What is the minimal starting volume of CSF for other EV analysis methods?

Q23. What is the minimal starting volume of CSF for other EV analysis metho...
10-20ul
TBD
200-500 micro liter
500µL
1 mL
3 ml
500ul
0.5 ml
1ml
3 ml
5mL
subjective
500
5 mL

Q24 - Q24. Should MISEV2018 guidelines be followed for CSF EV measures in publications (e.g. quantification of protein, lipid, nucleic acids, surface markers), with compliance stated?

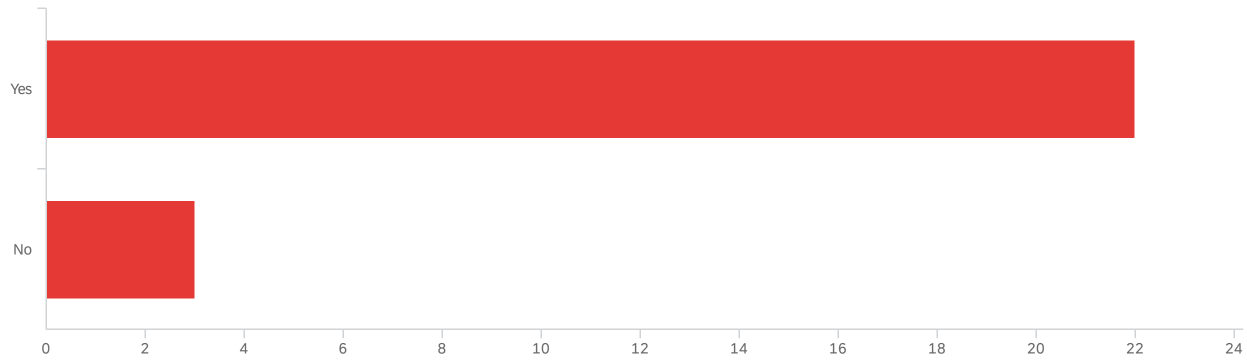

#	Field	Minimum	Maximum	Mean	Std Deviation	Variance	Count
1	Q24. Should MISEV2018 guidelines be followed for CSF EV measures in publications (e.g. quantification of protein, lipid, nucleic acids, surface markers), with compliance stated?	1.00	2.00	1.12	0.32	0.11	25

#	Field	Choice Count	
1	Yes	88.00%	22
2	No	12.00%	3
			25

Showing rows 1 - 3 of 3

Q25 - Please cite key papers on CSF EVs (PMID or DOI), or add additional comments here.

Please cite key papers on CSF EVs (PMID or DOI), or add additional comments...
NA
doi: 10.7150/thno.31502; doi: 10.1002/pmic.201800257; doi: 10.1080/20013078.2017.1369805; doi: 10.1080/20013078.2017.1317577; doi: 10.1093/brain/awv346; doi: 10.1080/20013078.2017.1348885; doi: 10.1016/j.celrep.2019.08.036; 10.1186/s12014-020-09294-7
https://doi.org/10.1186/s12974-019-1617-y
doi: 10.1080/20013078.2017.1369805. this is our only experience/concerted effort with CSF and we havent been able to progress it in MS

End of Report
