# Supplementary material for: Recommendations for reproducibility of cerebrospinal fluid extracellular vesicle studies
Source: J Extracell Vesicles. 2023 Dec 29;13(1):12397. doi: 10.1002/jev2.12397 (PMC10756860; doi:10.1002/jev2.12397)
Supplement: Supplementary file 3 — Supporting Information [file JEV2-13-12397-s002.docx]

| **Supplemental Form 1. Standardized and Detailed CSF Sample Collection Form** | | | |
| --- | --- | --- | --- |
| **CSF SAMPLE SOURCE:**  Lumbar puncture  EVD  Ventricular shunt  Drain  Other: | | | |
| Collection Date _____________  DD/MM/YY | Collection Time _________  HH/MM | | Fasting  Yes  No  Time of Last Meal ______AM/PM |
| Sedated Procedure  Yes  No  If Yes, which Anesthetic __________________  Needle Gauge ______________  Number of Tubes Collected  1  2  3  4 ☐ Other _________ | | Volume of CSF Collected  ☐ 1 ☐ 2 ☐ 3 ☐ 4 ☐ Other _________ mL  Total Volume Collected _____________ mL  Collection Temperature _____________°C  Transport Temperature _____________°C | |
| Type of Collection Tube _____________________  Were Individual Collection Tubes Pooled/Mixed Prior to Processing/Storage?  Yes  No  Additives to CSF  Yes  No Type ___________________ | | | |
| Centrifugation  Yes  No  Processing Time ____________  HH:MM  Centrifugation Speed: __________ ☐ g or ☐ rpm | | Centrifugation Duration _________  MM/SEC  Centrifugation Temperature ___________ °C  Ultrafiltration  Yes  No Device ______________ | |
| Number of Aliquots ________  Aliquot Volume ________ mL  Type of Storage Tube ____________________ | | CSF Sent for Clinical Biochemistries?  Yes  No  Visual Macroscopic Blood?  Yes  No | |
| Date of Transfer to Long Term Storage  _____________  DD/MM/YY  Storage Freezer Temperature ____________°C | | Time of Transfer to Long Term Storage  _________  HH/MM  Initials of Study Staff Completing Transfer _________ | |
| **Notes** | | **Staff Initials** | |
